# Supplementary figures and images for: A glycan receptor kinase facilitates intracellular accommodation of arbuscular mycorrhiza and symbiotic rhizobia in the legume Lotus japonicus
Source: PLoS Biol. 2023 May 18;21(5):e3002127. doi: 10.1371/journal.pbio.3002127 (PMC10231839; doi:10.1371/journal.pbio.3002127)

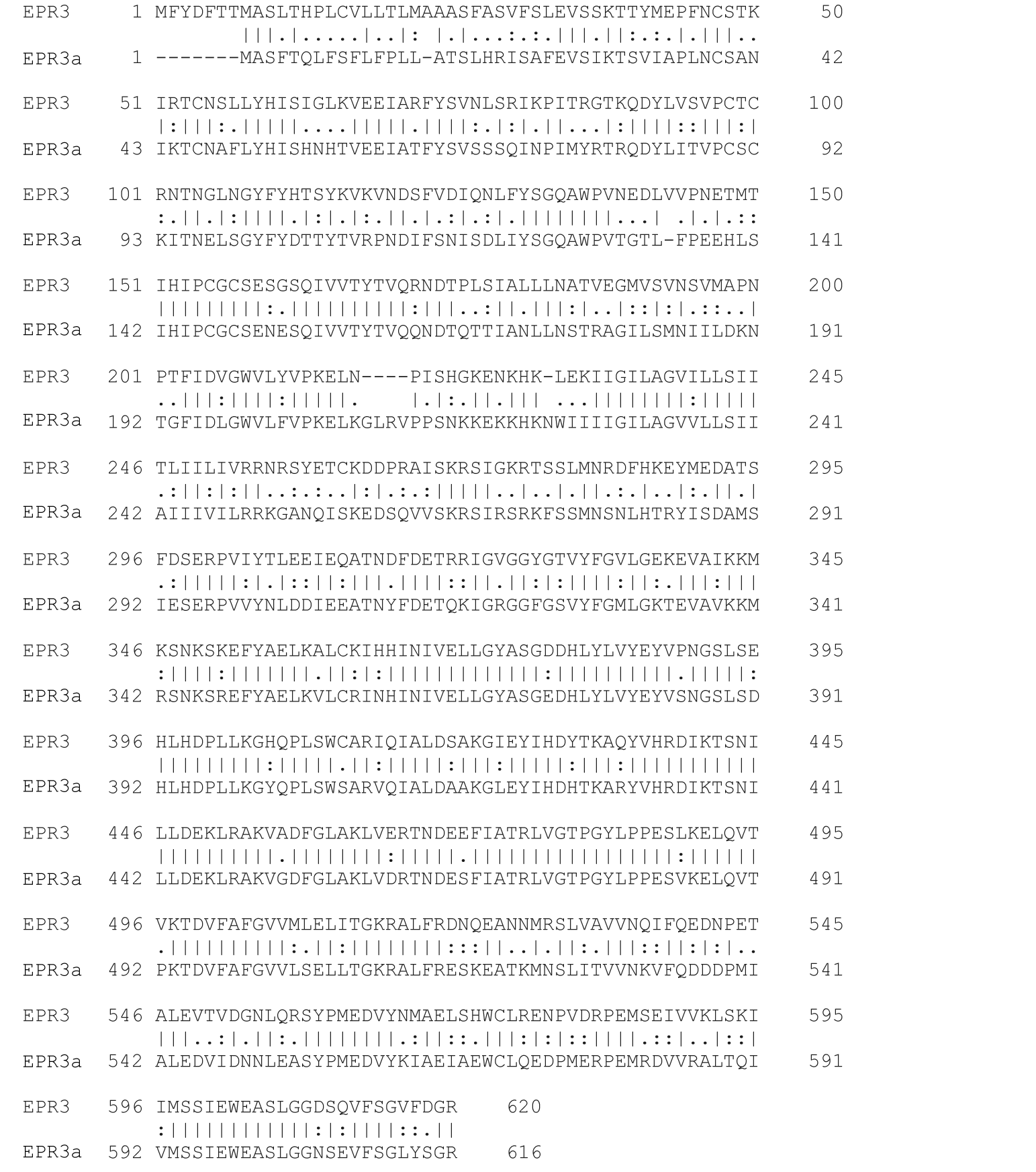

Supplement: S1 Fig — The “|” indicates perfect alignment, “:” indicates residues of similar properties, “.” indicates residues of dissimilar properties, and “-” indicates no alignment. (TIF) [file pbio.3002127.s001.tif]

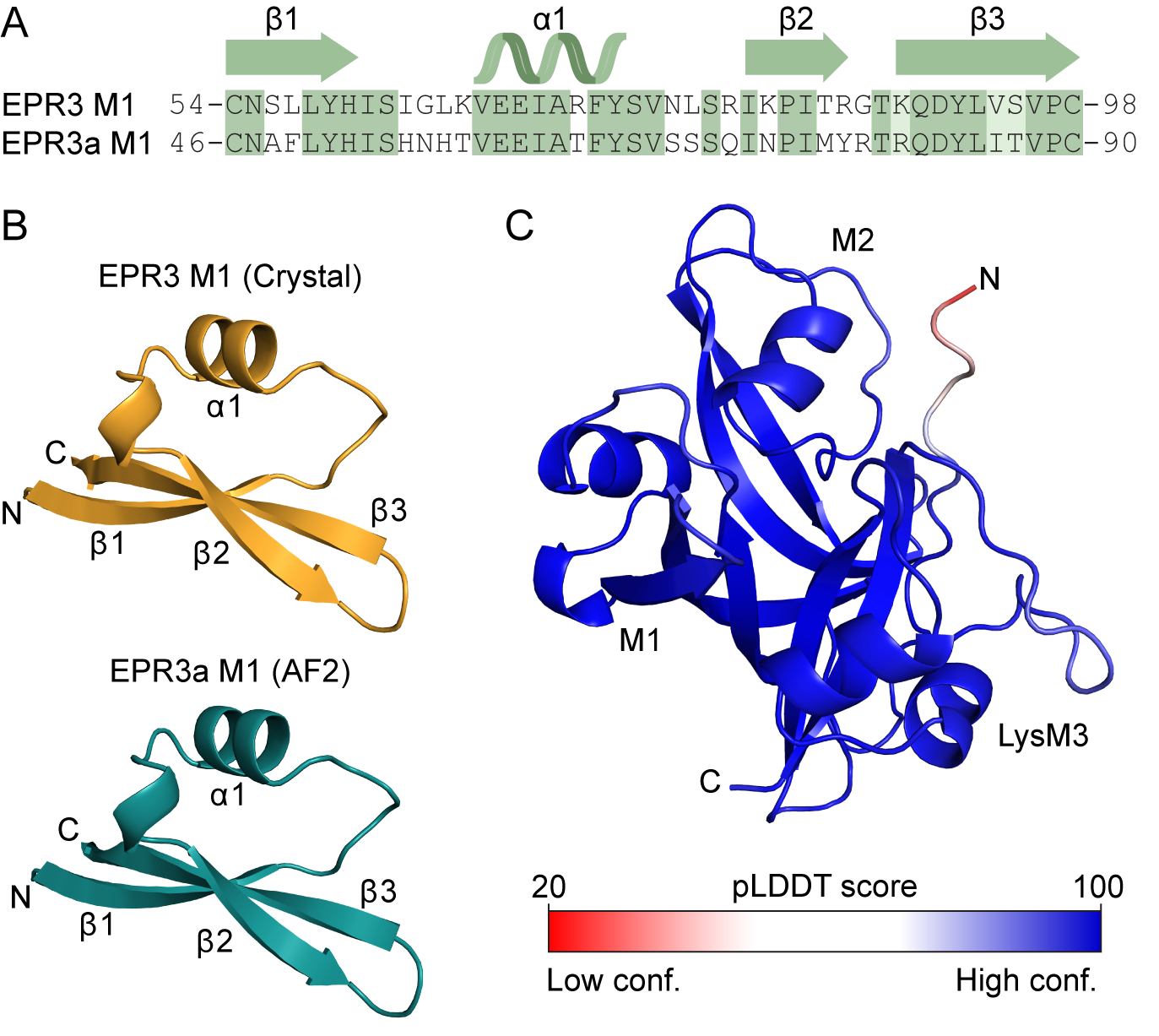

Supplement: S2 Fig — (A) Sequence alignment of the EPR3 and EPR3a M1 domain. Conserved and semi-conserved residues are highlighted in green and light green, respectively. The βαββ secondary structure signature of the EPR3 M1 crystal structure is indicated above the alignment. (B) Zoom of the EPR3 M1 crystal structure as compared to the Alphafold generated model of the EPR3a M1. EPR3a shows a strikingly high similarity to the EPR3 M1 with an identical βαββ structure. (C) The EPR3a ectodomain Alphafold model shown as a spectrum of the predicted local distance difference test (pLDDT) score. Blue colouring indicates a high pLDDT score and a high confidence in the modelled structure, whereas red colouring indicates a low pLDDT score and low confidence. See S1 Information for underlying data. (TIF) [file pbio.3002127.s002.tif]

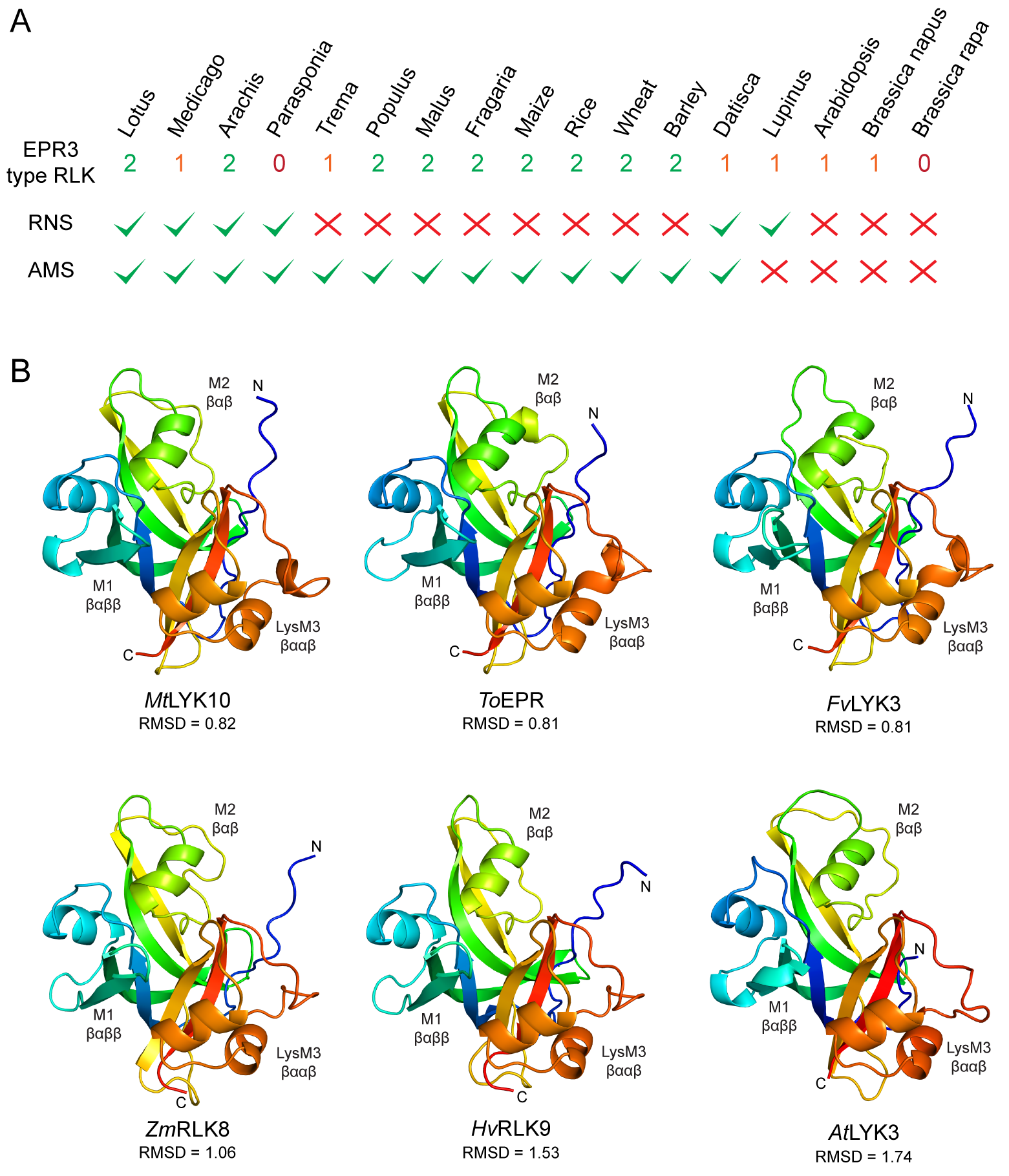

Supplement: S3 Fig — (A) One or more EPR3-type RLKs are present throughout plant species. Pseudogenization or lack of gene identification coincides in some cases with the inability of plant species to establish root nodule symbiosis (RNS) or arbuscular mycorrhizal symbiosis (AMS). (B) A representative selection of EPR3-type ectodomains throughout plants shows a conserved protein architecture as determined by Alphafold modelling. The EPR3-type M1 (βαββ), M2 (βαβ), and LysM3 (βααβ) are highlighted. Ectodomains are shown in a cartoon representation and are spectrum coloured from blue at the N-terminus to red at the C-terminus. The RMSD (in Å) of Cα superpositioning to the EPR3 crystal structure is reported and indicates the degree of structural resemblance. See S1 Information for underlying data. (TIF) [file pbio.3002127.s003.tif]

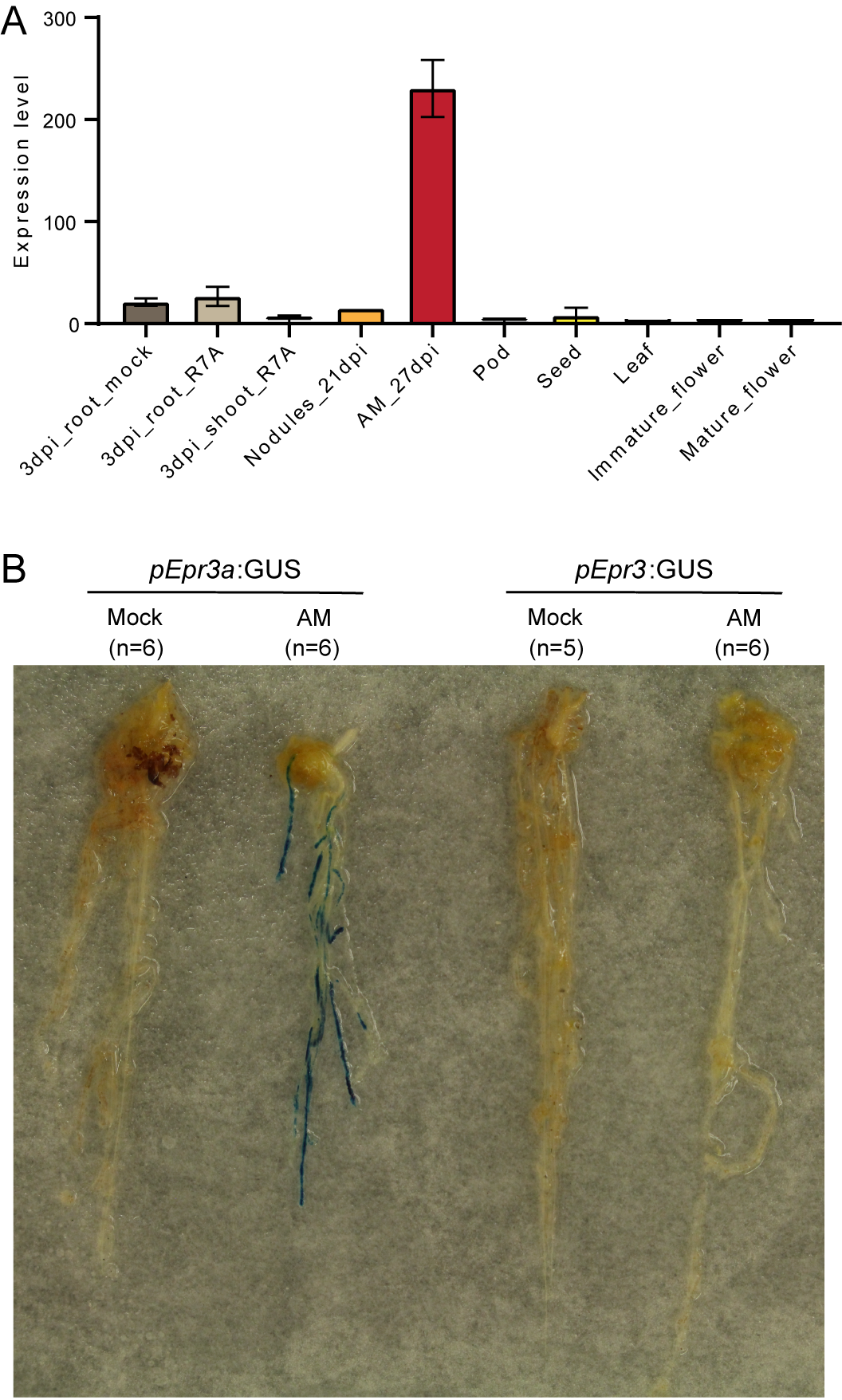

Supplement: S4 Fig — (A) Expression data obtained from the Lotus expression atlas (Lotus Base http://lotus.au.dk). Epr3a expression is restricted to root tissues with increased expression in response to arbuscular mycorrhiza (AM_27dpi). (B) Transgenic roots expressing pEpr3a:GUS or pEpr3:GUS were inoculated with AM spores. GUS staining was performed on whole root systems 6 wpi. See S1 Data for underlying data. (TIF) [file pbio.3002127.s004.tif]

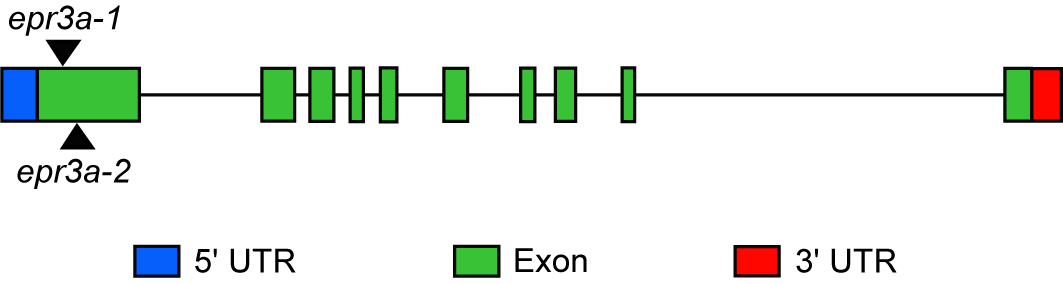

Supplement: S5 Fig — Epr3a gene model with the position of LORE1 insertions in isolated mutant alleles indicated. (TIF) [file pbio.3002127.s005.tif]

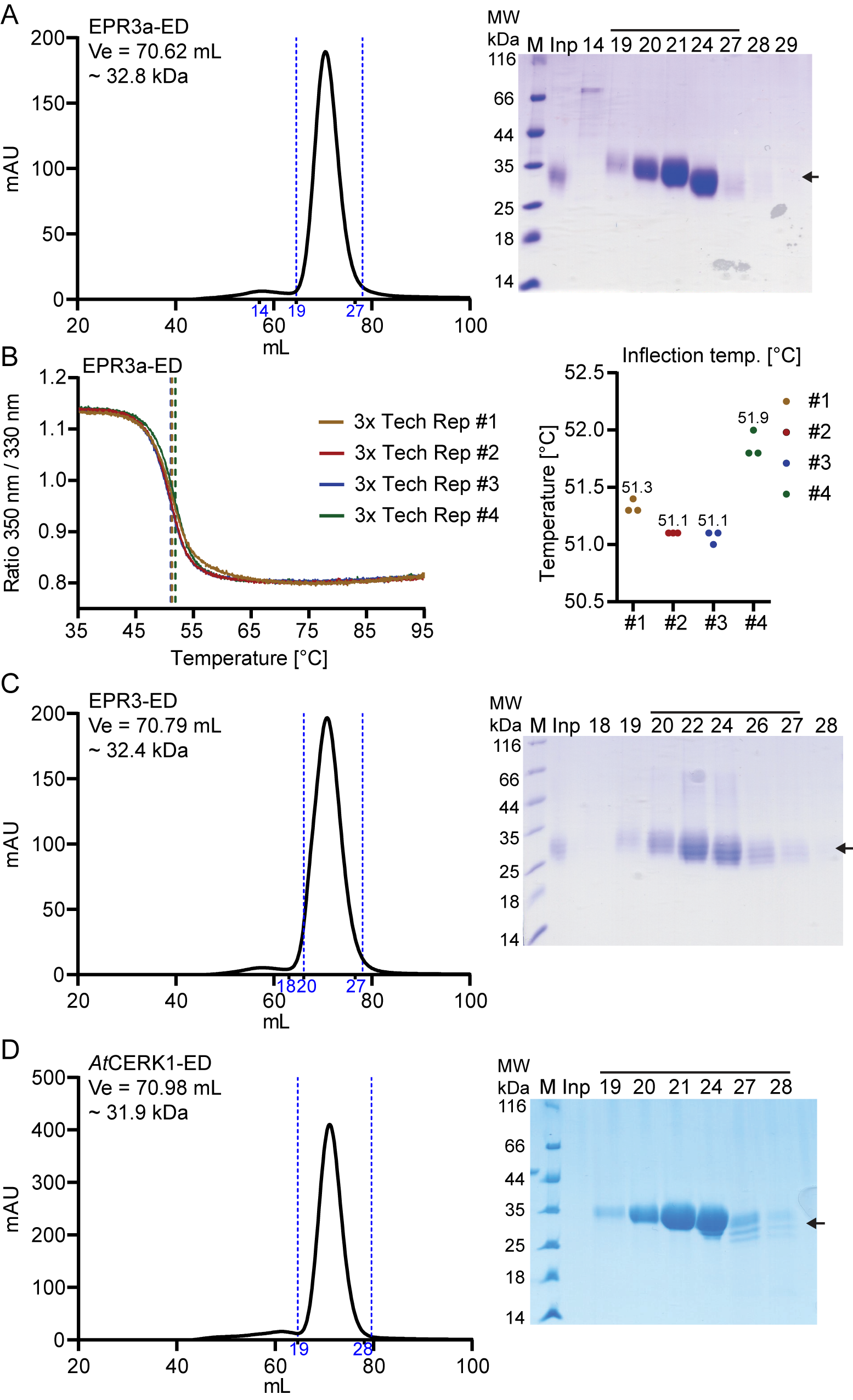

Supplement: S6 Fig — (A) SEC profile and corresponding SDS-PAGE of the final purification step for the EPR3a ectodomain. Protein elutes as a single peak with an elution volume corresponding to a molecular weight of 32.8 kDa. SDS-PAGE analysis reveals a smeared band between the 25 and 35 kDa marker bands, fitting the weight estimate from SEC and an expected heterogenous N-glycosylated protein preparation. Weight estimates from SEC and SDS-PAGE fit well the theoretical molecular weight of 23.2 kDa for the monomeric protein, plus an additional average of approximately 10 kDa N-glycans. (B) A NanoDSF thermal stability assay was used as a quality control for protein preparations and to determine confidence in comparability between replicates in downstream binding assays. The thermal stability of 4 EPR3a ectodomain biological replicates was assayed with NanoDSF, showing that all 4 preparations had similar Ti. Each biological preparation was assayed in technical triplicates. (C, D) SEC chromatogram and corresponding SDS-PAGE for EPR3 and AtCERK1 ectodomain purifications. Both preparations, like EPR3a, elute as single peaks fitting monomeric N-glycosylated proteins and migrate as smeared bands between marker bands 25 and 35 kDa in SDS-PAGE. (A, C, D) M = molecular weight marker, Inp = input sample of SEC purification. Blue numbering in chromatograms corresponds to different fractions, which are also indicated in the corresponding SDS-PAGE. Blue dashed lines in chromatograms and the horizontal black lines above fraction numbering in SDS-PAGEs indicates the pooled fractions used in biochemical assays. All protein preparations were purified to a high >95% purity as estimated by SDS-PAGE. See S1 Data and S1 Raw Images for underlying data. (TIF) [file pbio.3002127.s006.tif]

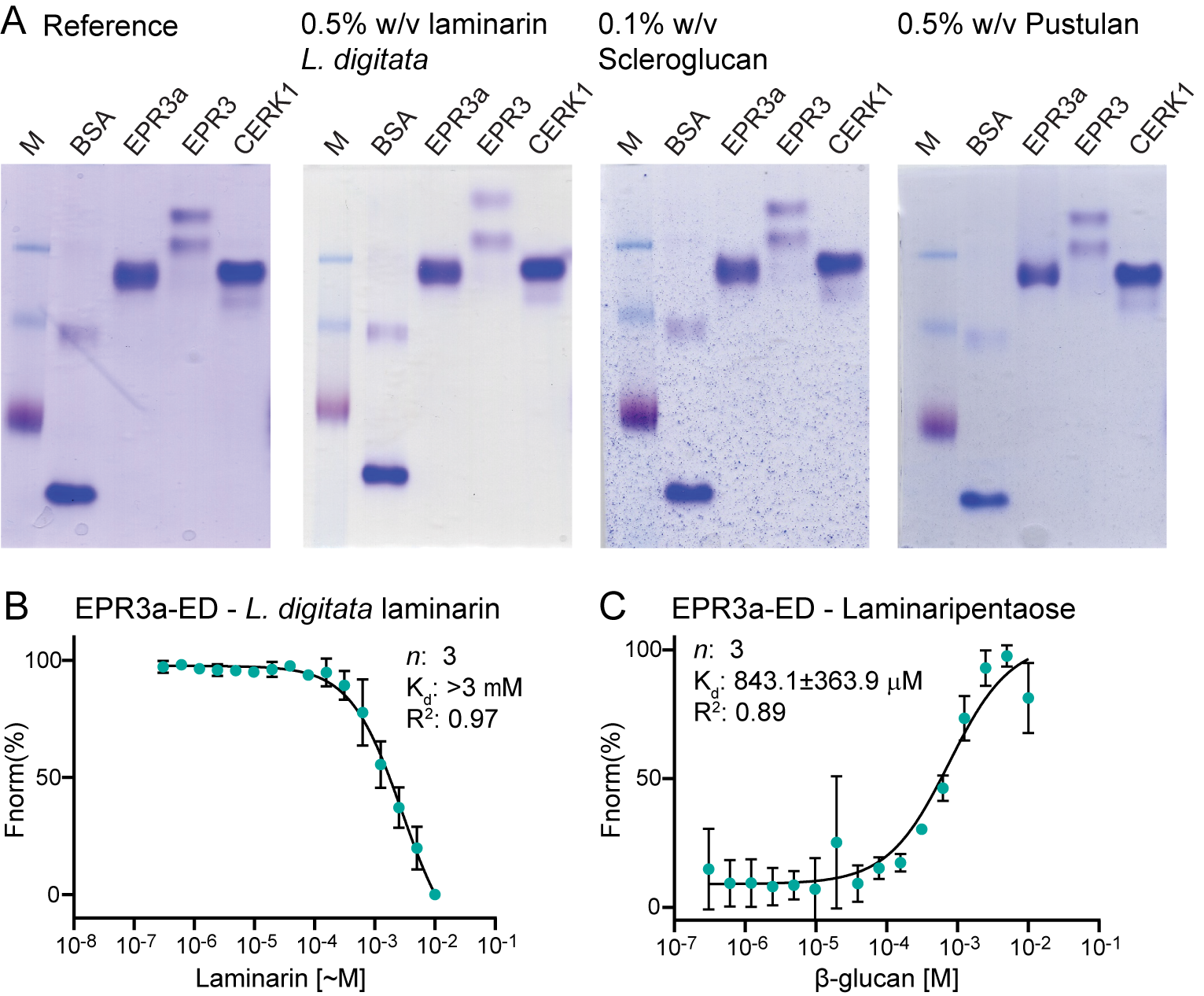

Supplement: S7 Fig — (A) Affinity gel electrophoresis assays using β-glucans L. digitata laminarin (β-1,3/β-1,6), Scleroglucan (β-1,3/β-1,6), or Pustulan (β-1,6) did not show retention of EPR3a, EPR3, or AtCERK1 ectodomains. M indicates PageRuler Prestained Protein Ladder, 10 to 180 kDa (Thermo Fisher), and BSA indicates bovine serum albumin, both of which were included as markers to gauge retention of LysM ectodomains. (B, C) MST binding data showing EPR3a binds L. digitata laminarin and laminaripentaose with low affininty (Kd >3 mM and ≈ 850 μM, respectively). Fnorm(%) is the measured normalised fluorescence of ectodomains assayed over a ligand concentration series, n denotes the number of biological replicates, Kd is the calculated dissociation constant, and the goodness of fit is given by R2. See S1 Data and S1 Raw Images for underlying data. (TIF) [file pbio.3002127.s007.tif]

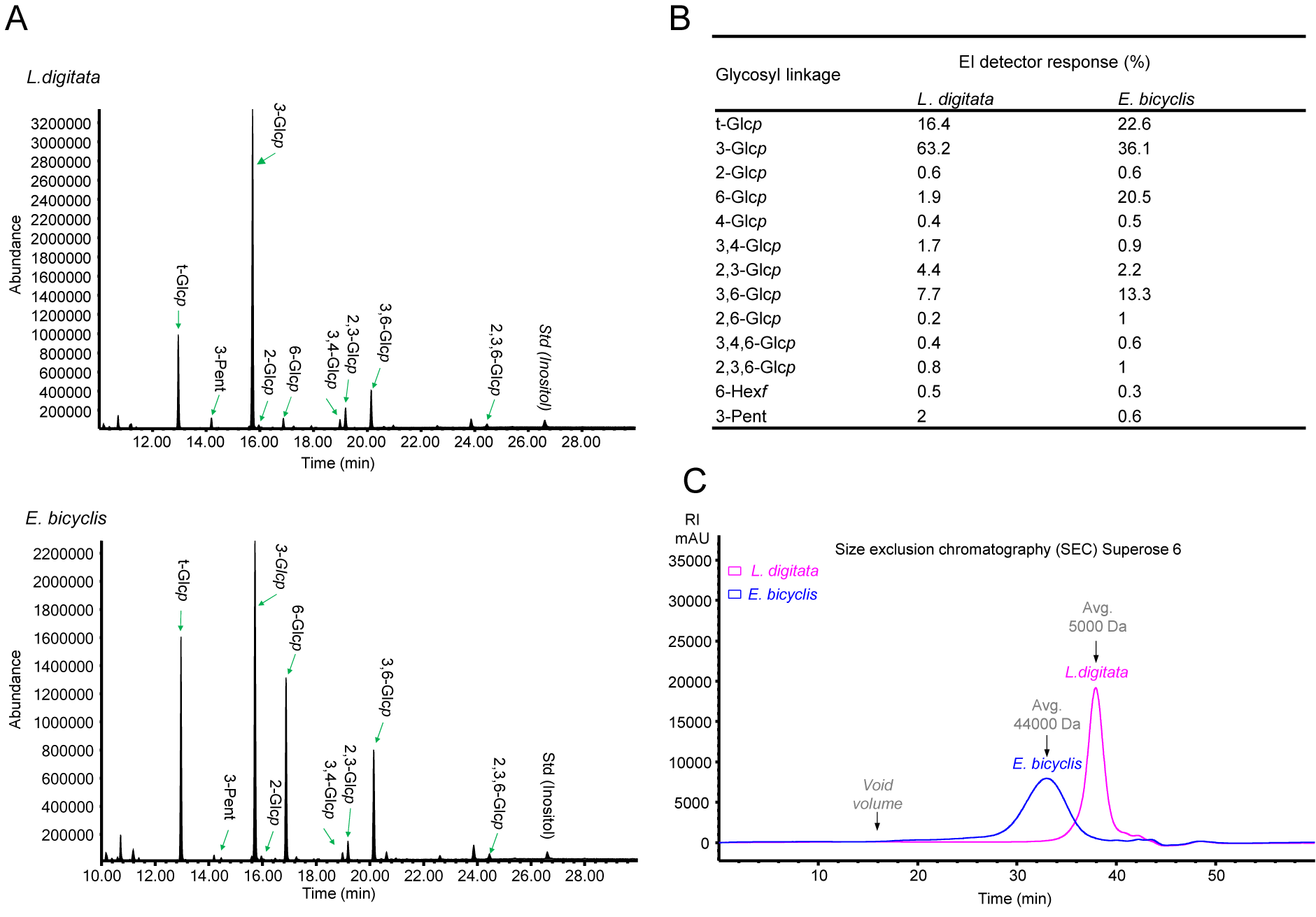

Supplement: S8 Fig — (A) Determination of glycosyl linkages in L. digitata laminarin (top) and E.bicyclis laminarin (bottom). (B) The relative distribution (in %) of glycosyl linkages in L. digitata and E.bicyclis laminarins. (C) Determination of the molecular weight of the soluble fraction by size exclusion chromatography on a Superose 6 column. The average MW of L. digitata (magenta) is 5,000 Da, and E. bicyclis (blue) is approx. 44,000 Da. See S4 Information for underlying data. (TIF) [file pbio.3002127.s008.tif]

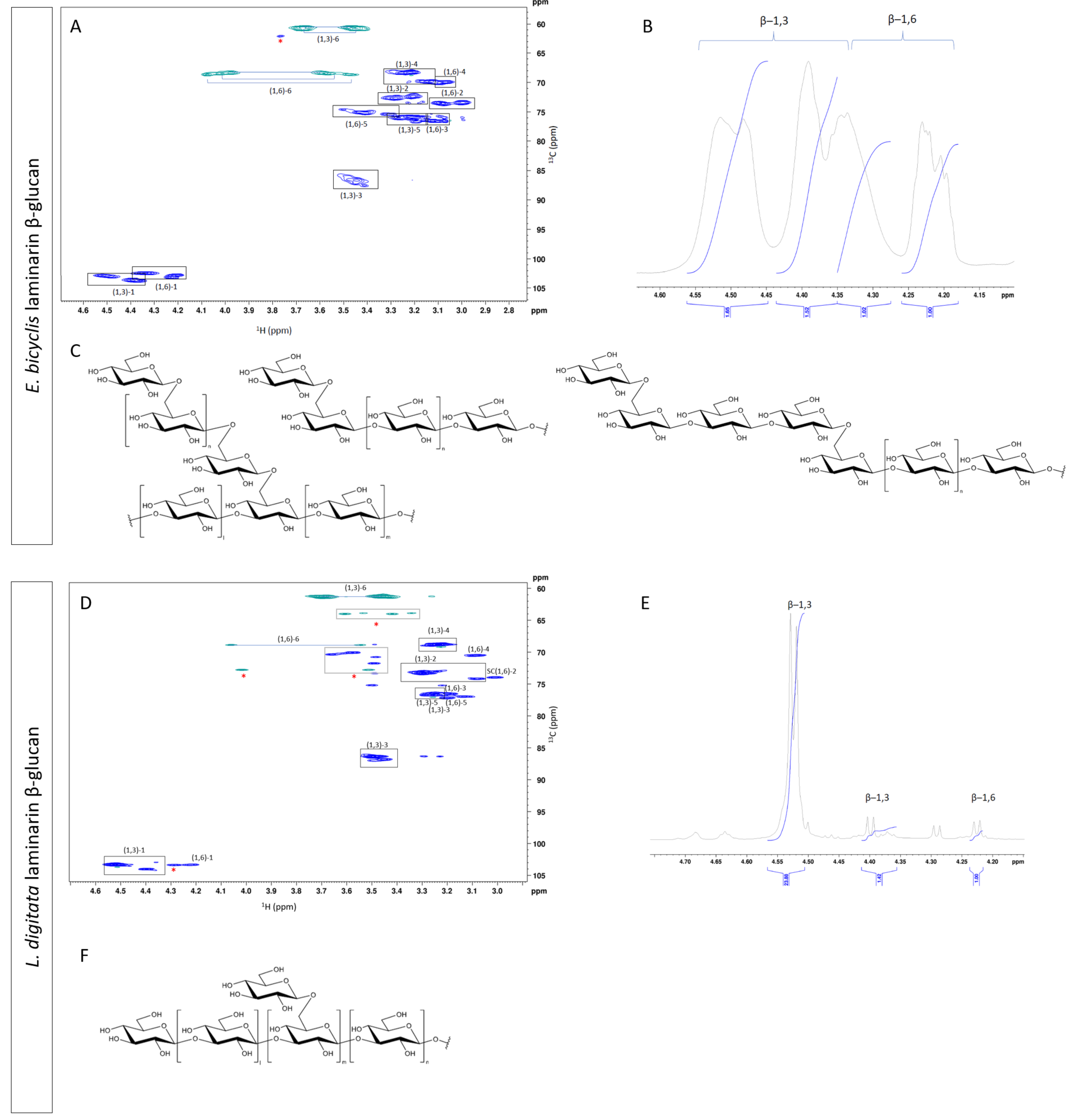

Supplement: S9 Fig — (A–C) E. bicyclis laminarin contains a 1,3 linked backbone with a complex mix of at least 3 different types of 1,6 branches. (D–F) L. digitata laminarin is a 1,3 linked main chain with 1,6 branched terminal glucose. (A) 1H-13C-HSQC of E. bicyclis laminarin (11.4 mg) in 90% DMSO-d6 and 10% D2O at 50 °C. The signal labelled with a red asterisk belongs to an unknown impurity. (B) Based on 1H peak integration, the ratio between 1,3 and 1,6 linkages is 3:2. (C) Chemical structures found in E. bicyclis laminarin. The degree of branching and the spacing of branches cannot be determined. (D) 1H-13C-HSQC of L. digitata laminarin (11.3 mg) in 90% DMSO-d6 and 10% D2O at 60 °C. Signals labelled with a red asterisk belong to mannitol located on the reducing end of approximately half of the oligomers, which is consistent with previous studies [32,83]. (E) Based on 1H peak integration, 1,3:1,6 linkage ratio is 25:1. (F) Chemical structure found in L. digitata laminarin. The degree and spacing of branching are too ambiguous to determine. See S5 Information for underlying data. (TIF) [file pbio.3002127.s009.tif]

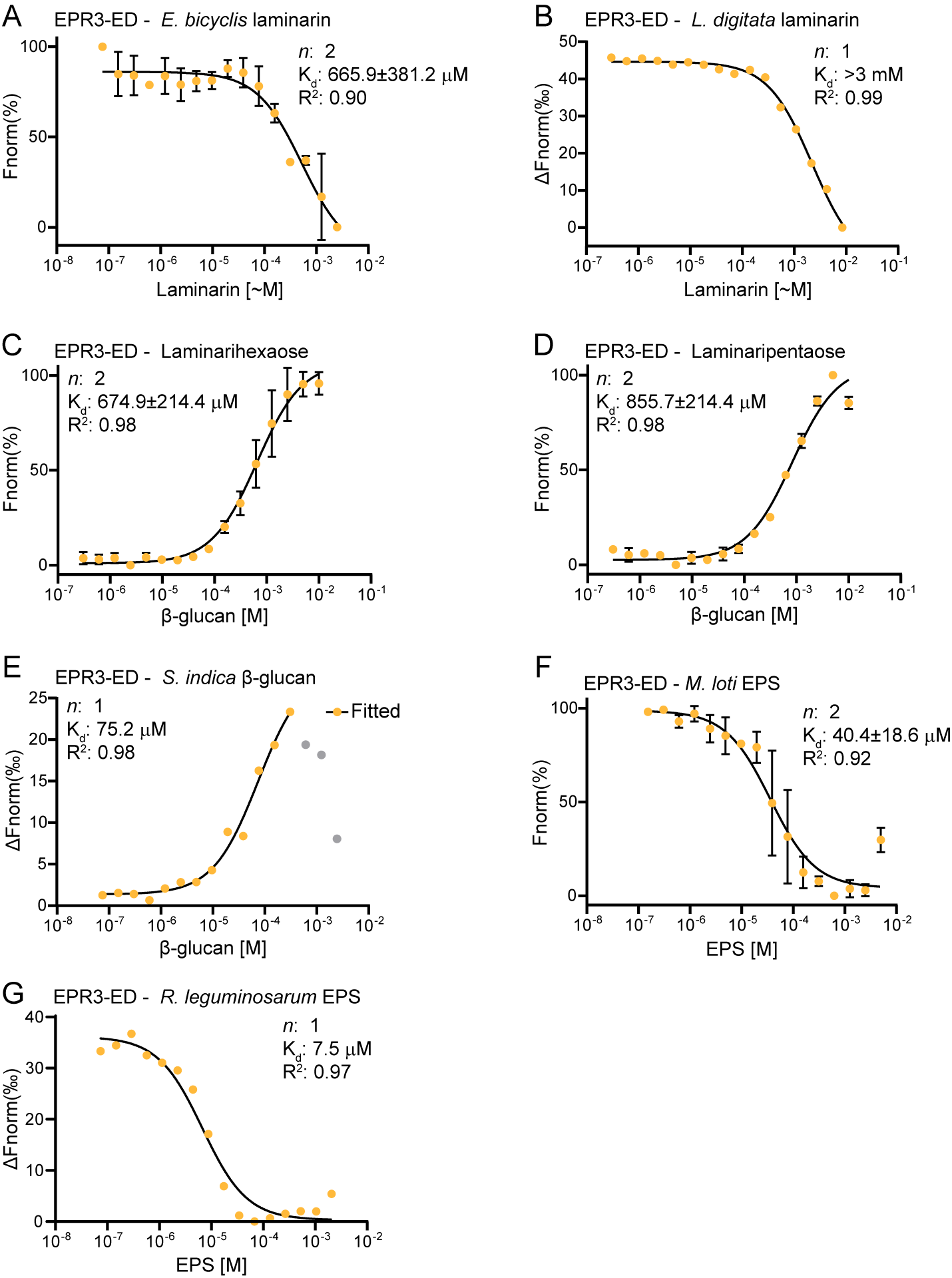

Supplement: S10 Fig — (A) The EPR3 ectodomain binds E. bicyclis laminarin with atleast 2-fold lower affinity ≈ 670 μM compared to EPR3a (Kd ≈ 250 μM) when assayed with MST. (B–G) EPR3 had similar affinities for other ligands measured as that observed for EPR3a. Binding affinities for (F) M. loti and (G) R. leguminosarum EPS was similar as previously reported [22]. (A–G) Fnorm(%) is the measured normalised fluorescence of ectodomains assayed over a ligand concentration series, and ΔFnorm(‰) is the normalised difference in fluorescence of experiments with a single biological replicate. n denotes the number of biological replicates, Kd is the calculated dissociation constant, and the goodness of fit is given by R2. See S1 Data for underlying data. (TIF) [file pbio.3002127.s010.tif]

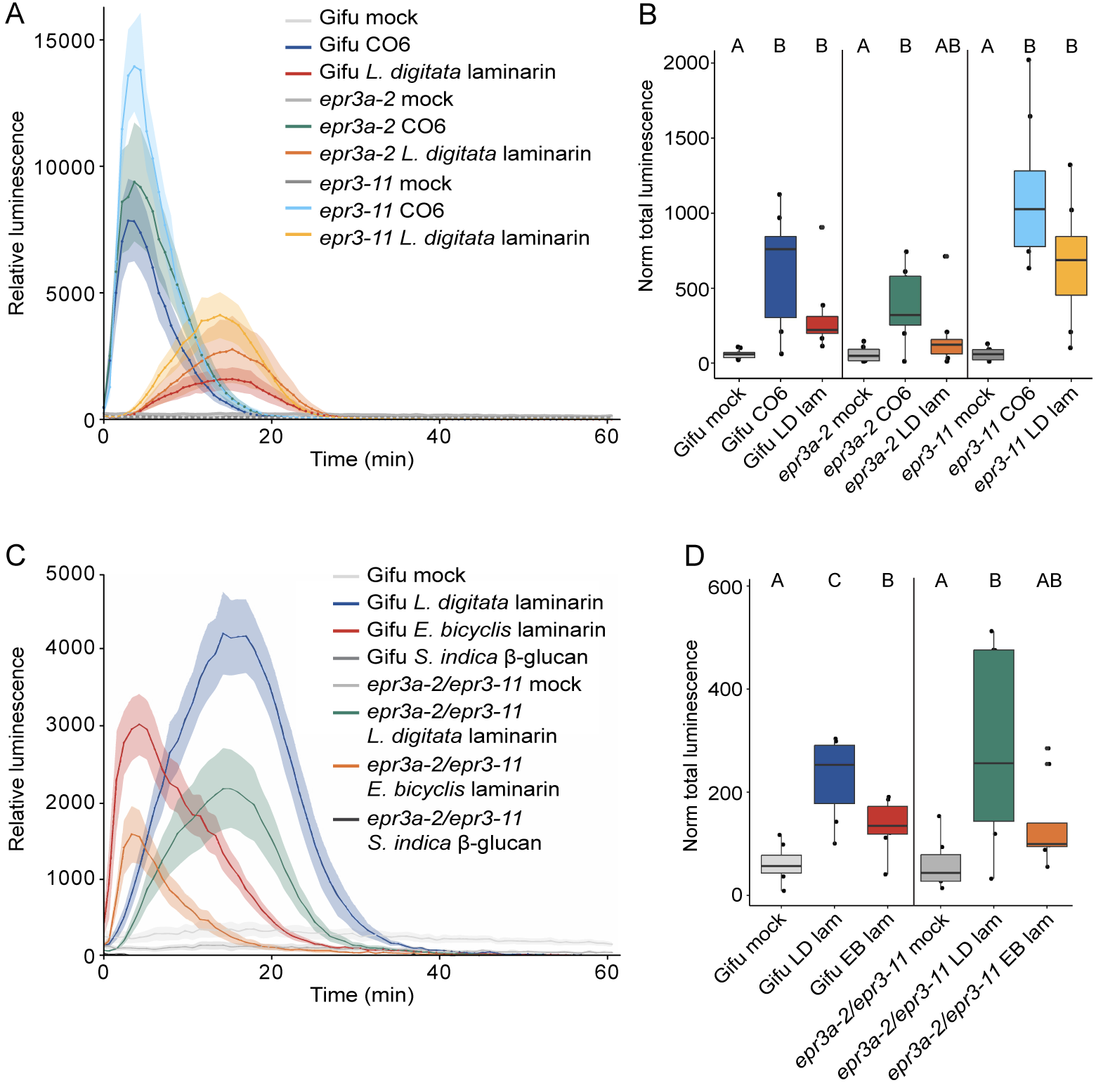

Supplement: S11 Fig — (A) ROS production measured over time in response to mock, chitohexaose (CO6, positive control), and L. digitata laminarin. CO6 elicits a fast and strong ROS response in Lotus Gifu, epr3-11 and epr3a-2. L. digitata laminarin elicits a delayed and relatively weaker ROS response compared to CO6. (B) Boxplot of normalised total ROS production measured over 60 min. L. digitata laminarin (LD lam) ROS elicitation is not significantly affected in epr3-11 or epr3a-2 compared to Gifu. Values represent means ± SEM from 8 wells. Letters represent significant differences based on Kruskal–Wallis and post hoc Dunn test. (C) E. bicyclis laminarin elicits a relatively faster and weaker ROS burst in Gifu and epr3-11/epr3a-2 compared to L. digitata laminarin. A single well/replicate was performed for the S. indica β-glucan decasaccharide in Gifu and epr3-11/epr3a-2 and no ROS elicitation was detected. (D) Boxplot of normalised total ROS production measured over 60 min. E. bicyclis (EB lam) and L. digitata laminarin (LD lam) ROS elicitation is not significantly affected in epr3-11/epr3a-2 compared to Gifu. Values represent means ± SEM from 8 wells. Letters represent significant differences based on Kruskal–Wallis and post hoc Dunn test. See S1 Data for underlying data. (TIF) [file pbio.3002127.s011.tif]

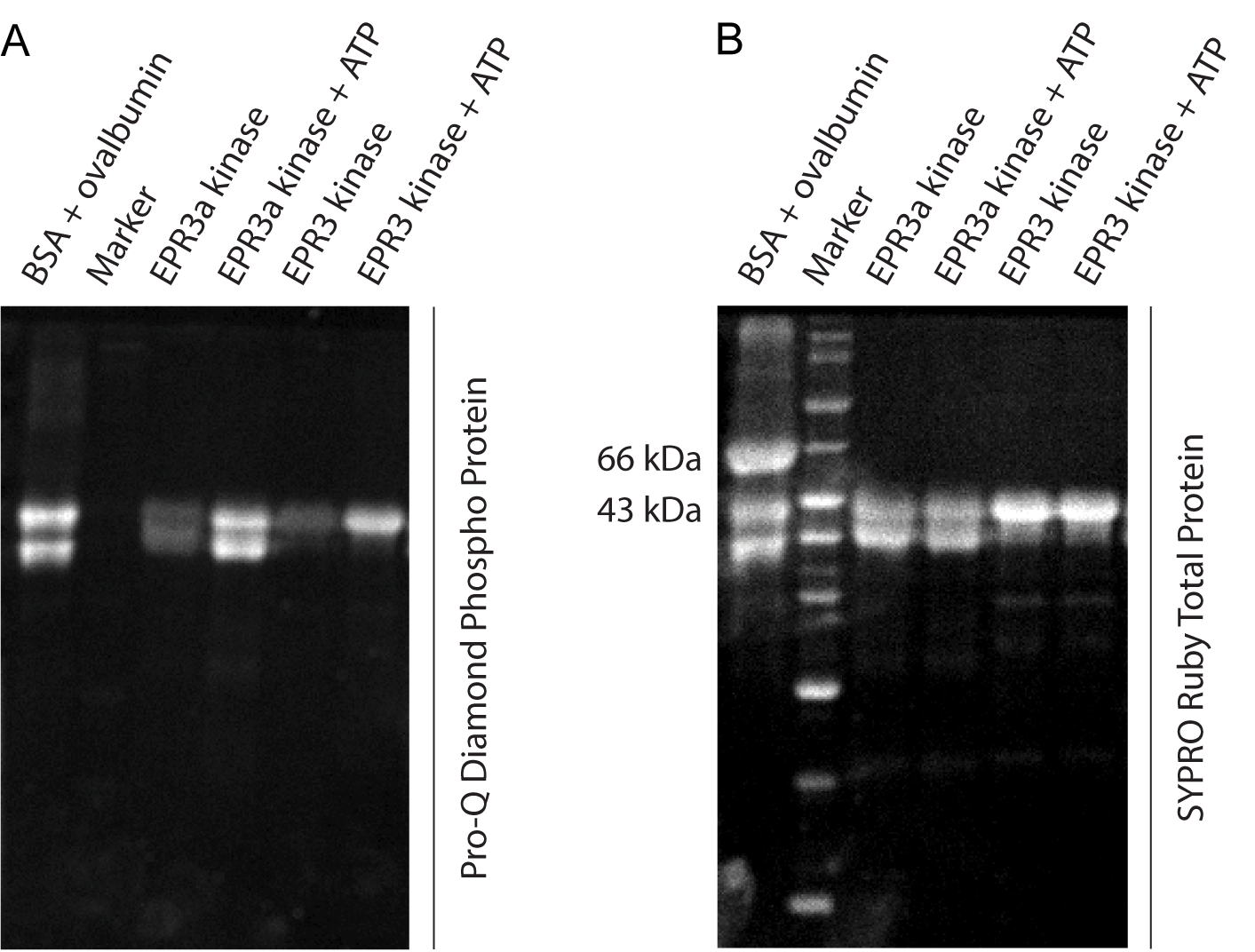

Supplement: S12 Fig — (A) EPR3a and EPR3 intracellular kinase domains purified from E. coli were incubated with or without ATP+MgCl2 and subsequently analysed by SDS-PAGE and Pro-Q Diamond phosphoprotein gel stain. Both EPR3a and EPR3 are able to autophosphorylate, as shown by the enhanced stain intensity in ATP+MgCl2 incubated samples. (B) The same SDS-PAGE as in (A) stained with SYPRO Ruby total protein stain. BSA (non-phosphorylated) and ovalbumin (phosphorylated) were included as markers for phosphorylation. See S1 Raw Images for underlying data. (TIF) [file pbio.3002127.s012.tif]

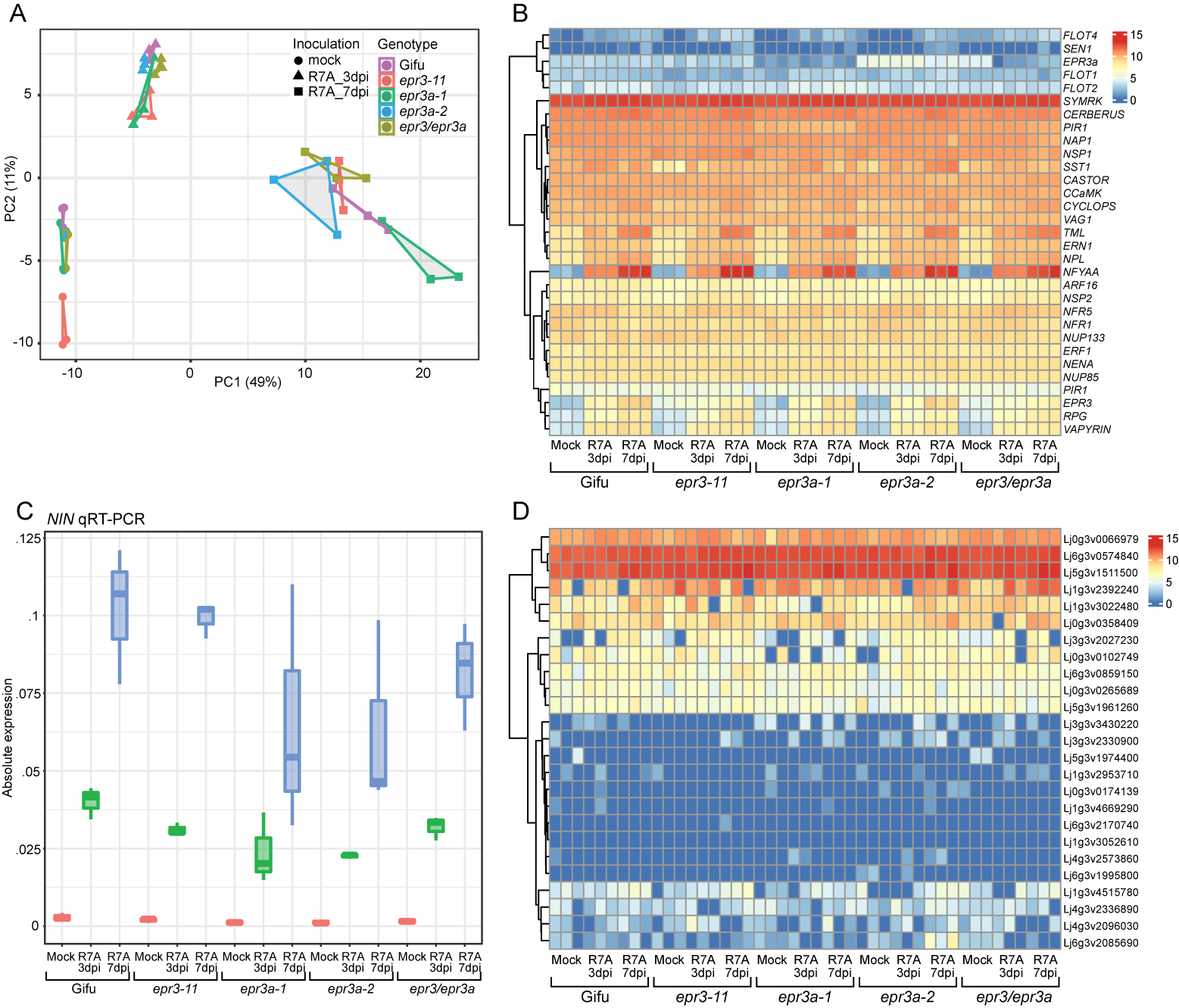

Supplement: S13 Fig — (A) Principal component analysis of RNA-seq data obtained from roots of wild-type and mutant plants inoculated with M. loti R7A. RNA-seq was performed on root samples harvested 3 and 7 dpi. (B) Expression of known symbiotic genes was comparable in wild-type and receptor mutant plants. (C) qRT-PCR analysis showed the NIN expression profile was comparable in wild-type and receptor mutant plants. (D) The 25 Lotus genes that show the highest transcriptional response to pathogenic Ralstonia were chosen to represent defence-related genes. No significant difference in the expression of the genes was identified between wild-type and mutant plants. See S2 and S3 Information, NCBI BioProject accession: PRJNA953045 for underlying data. (TIF) [file pbio.3002127.s013.tif]
