# Supplementary material for: A glycan receptor kinase facilitates intracellular accommodation of arbuscular mycorrhiza and symbiotic rhizobia in the legume Lotus japonicus
Source: PLoS Biol. 2023 May 18;21(5):e3002127. doi: 10.1371/journal.pbio.3002127 (PMC10231839; doi:10.1371/journal.pbio.3002127)
Supplement: S1 Table — Only LORE1 inserts in the Epr3a gene are shared between the epr3a-1 and epr3a-2 lines. (DOCX) [file pbio.3002127.s014.docx]

| Mutant line | Gene | Gene annotation |
| --- | --- | --- |
| *epr3a-1* |  |  |
| LORE1 line 30014218 | [Lj0g3v0307059](https://lotus.au.dk/view/gene/Lj0g3v0307059) | EPR3a |
|  | [Lj0g3v0109639](https://lotus.au.dk/view/gene/Lj0g3v0109639) | DUF246 domain-containing protein |
|  |  |  |
| *epr3a-2* |  |  |
| LORE1 line 30155999 | [Lj0g3v0307059](https://lotus.au.dk/view/gene/Lj0g3v0307059) | EPR3a |
|  | [Lj1g3v5020900](https://lotus.au.dk/view/gene/Lj1g3v5020900) | 121F-specific p53 inducible RNA |
|  | [Lj3g3v0300690](https://lotus.au.dk/view/gene/Lj3g3v0300690) | transcriptional regulator ATRX-like |
|  | [Lj3g3v3281620](https://lotus.au.dk/view/gene/Lj3g3v3281620) | Uncharacterized protein |
|  | [Lj6g3v0933430](https://lotus.au.dk/view/gene/Lj6g3v0933430) | differentially expressed in FDCP 6 homolog |
|  |  |  |

**Suppl. Table 1**. **LORE1 exonic insertions in *epr3a-1* and *epr3a-2* mutant lines.** Only LORE1 inserts in the *Epr3a* gene are shared between the *epr3a-1 and epr3a-2* lines.
