# Supplementary material for: A glycan receptor kinase facilitates intracellular accommodation of arbuscular mycorrhiza and symbiotic rhizobia in the legume Lotus japonicus
Source: PLoS Biol. 2023 May 18;21(5):e3002127. doi: 10.1371/journal.pbio.3002127 (PMC10231839; doi:10.1371/journal.pbio.3002127)
Supplement: S2 Table — (DOCX) [file pbio.3002127.s015.docx]

| Target gene | Forward primer (5’-3’) | Reverse primer (5’-3’) |
| --- | --- | --- |
| *Atp* | CAATGTCGCCAAGGCCCATGGTG | AACACCACTCTCGATCATTTCTCTG |
| *Ubi* | atgtgcattttaagacaggg | gaacgtagaagattgcctgaa |
| *Epr3* | GTCTTCAGCGGGGTATTTGA | TGGCAGCAGTTTTGAACAAG |
| *EPR3a* | TGAACTCACTTATTACTGTTGTTA | GATTATTATCTATGACATCTTCTA |
| *Pt4* | GTACAATGACCTCATGGTTCT | CGTTCATCTCGAAATCCTTATC |

**Suppl. Table 2**. Primers used in qRT-PCR experiments.
