# Supplementary material for: A glycan receptor kinase facilitates intracellular accommodation of arbuscular mycorrhiza and symbiotic rhizobia in the legume Lotus japonicus
Source: PLoS Biol. 2023 May 18;21(5):e3002127. doi: 10.1371/journal.pbio.3002127 (PMC10231839; doi:10.1371/journal.pbio.3002127)
Supplement: S4 Information — (PDF) [file pbio.3002127.s020.pdf]

```
File       :E:\AA4970.D
Operator   : Artur
Acquired   : 17 Apr 2021  14:19     using AcqMethod SERVLABPMAA10TO1.M
Instrument :   GC-MS AA
Sample Name: L.digitata Laminarin
Misc Info  :
Vial Number: 44
```

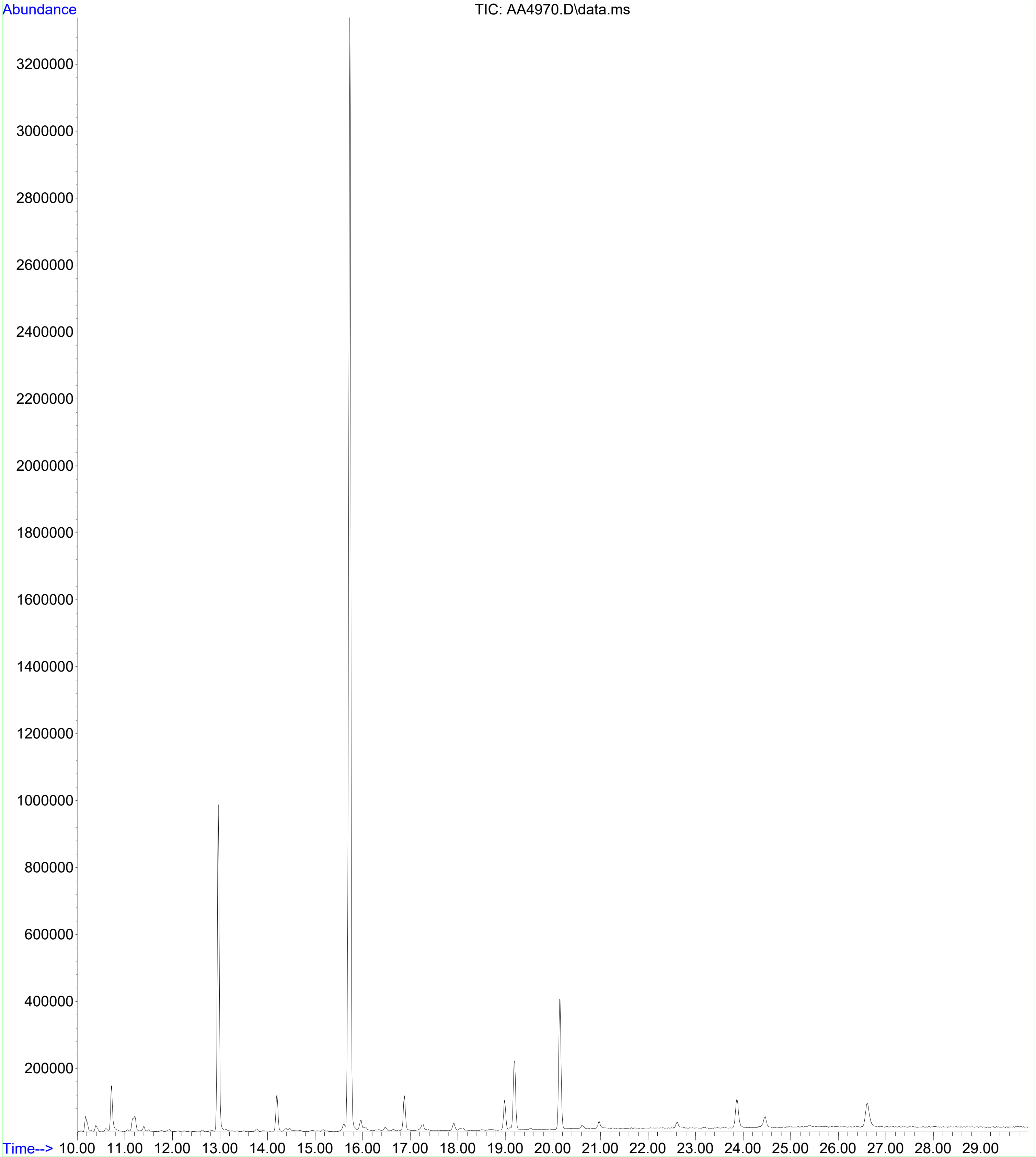

TIC: AA4970.D\data.ms
L.digitata Laminarin

Peak #	Ret Time	Type	Width	Area	Start Time	End Time
1	10.723	VB	0.047	4114240	10.664	10.891
2	12.969	BV	0.048	28351401	12.88	13.102
3	13.772	M	0.061	310778	13.719	13.839
4	14.201	BB	0.05	3421300	14.12	14.305
5	14.388	M	0.061	269083	14.325	14.421
6	14.464	M	0.064	323251	14.425	14.51
7	15.607	M	0.058	757568	15.525	15.633
8	15.736	VV	0.053	1.09E+08	15.639	15.886
9	15.961	M	0.057	974759	15.903	16.011
10	16.059	M	0.068	427009	16.015	16.111
11	16.475	M	0.071	446556	16.416	16.547
12	16.88	BB	0.051	3270958	16.793	16.996
13	17.26	M	0.067	726616	17.141	17.312
14	17.916	M	0.061	850741	17.824	17.996
15	18.105	M	0.113	540409	18.006	18.206
16	18.99	BV	0.052	2873406	18.911	19.067
17	19.195	VB	0.057	7574199	19.067	19.337
18	20.151	BB	0.054	13336195	20.049	20.28
19	20.618	M	0.063	389013	20.539	20.679
20	20.972	M	0.065	881455	20.891	21.051
21	22.61	M	0.061	633582	22.549	22.67
22	23.873	BB	0.072	3948600	23.758	24.036
23	24.461	M	0.076	1400693	24.349	24.569
24	26.609	M	0.099	4153762	26.479	26.791

File   :E:\AA4970.D
Operator   : Artur
Acquired   : 17 Apr 2021   14:19   using AcqMethod SERVLABPMAA10TO1.M
Instrument :   GC-MS AA
Sample Name: L.digitata Laminarin
Misc Info   :
Vial Number: 44

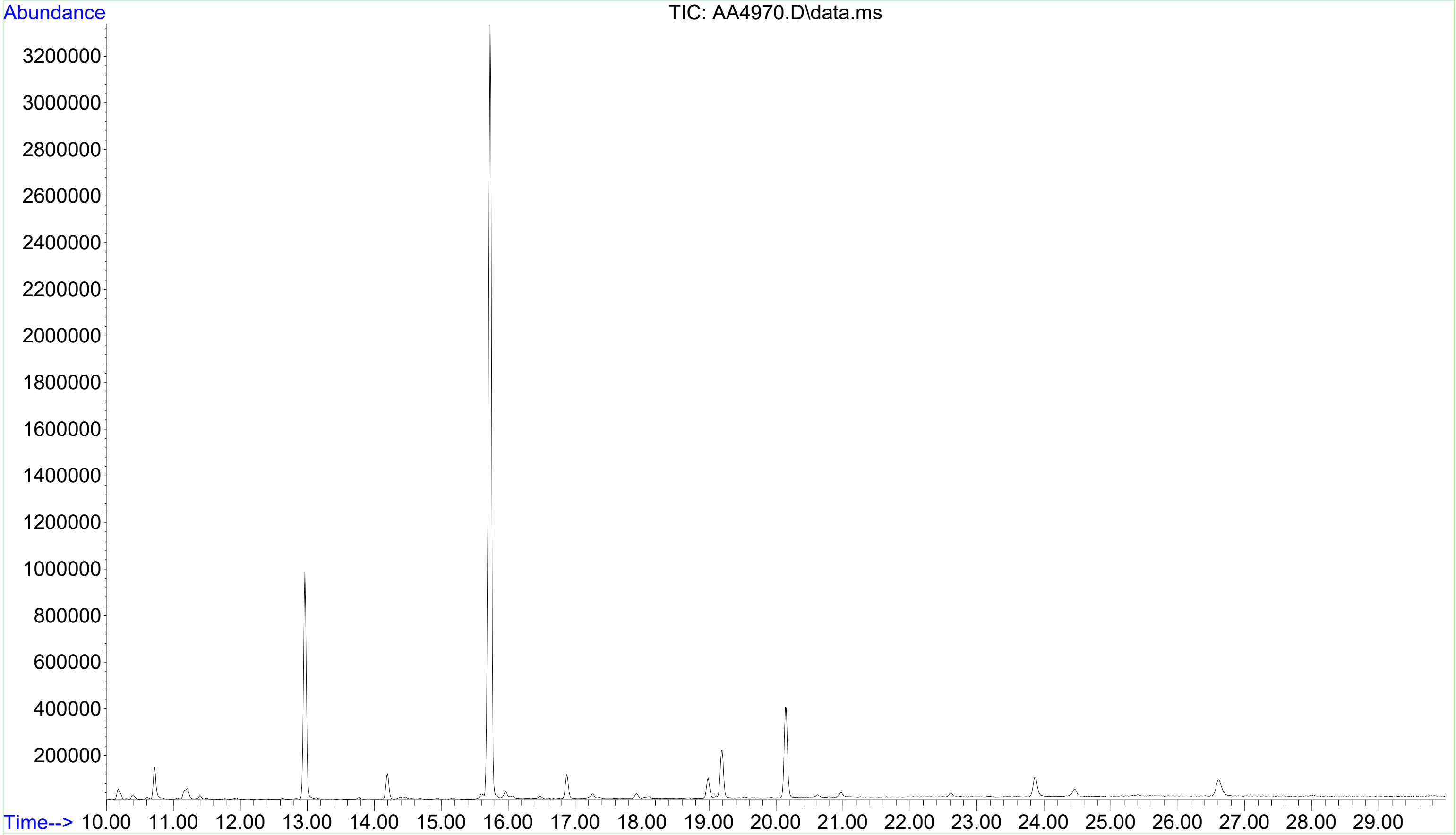

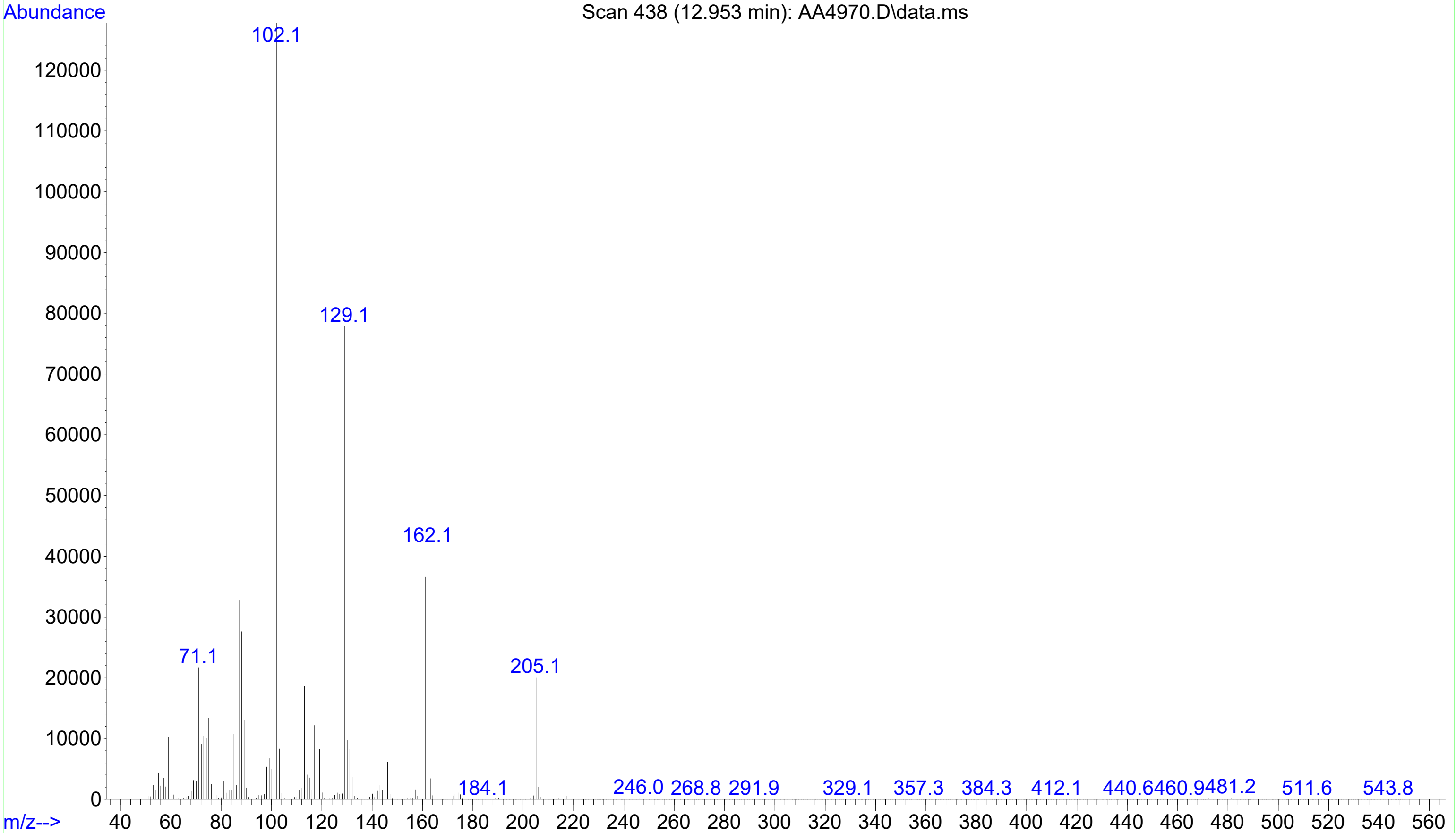

File   :E:\AA4970.D
Operator   : Artur
Acquired   : 17 Apr 2021   14:19   using AcqMethod SERVLABPMAA10TO1.M
Instrument :   GC-MS AA
Sample Name: L.digitata Laminarin
Misc Info   :
Vial Number: 44

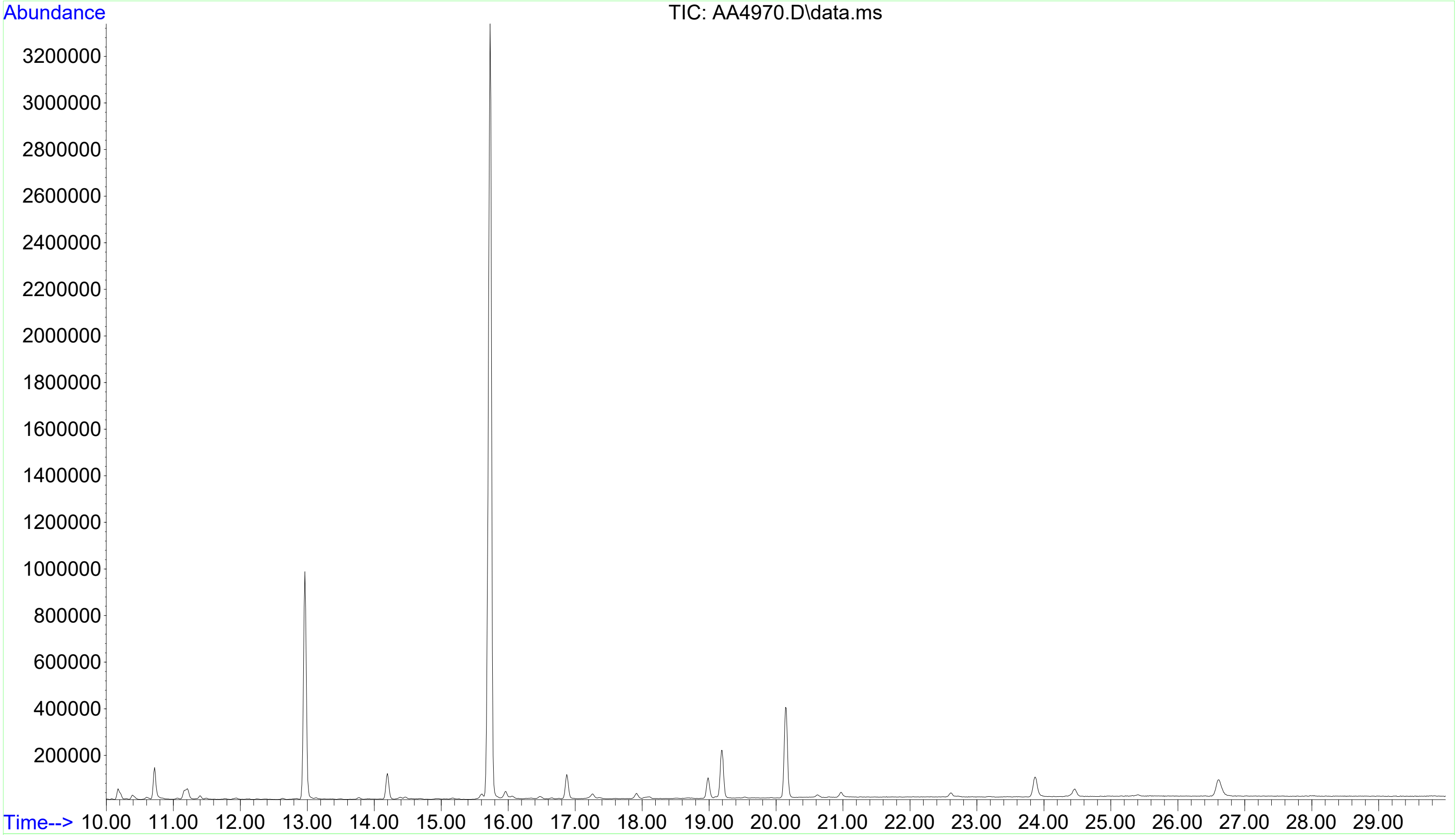

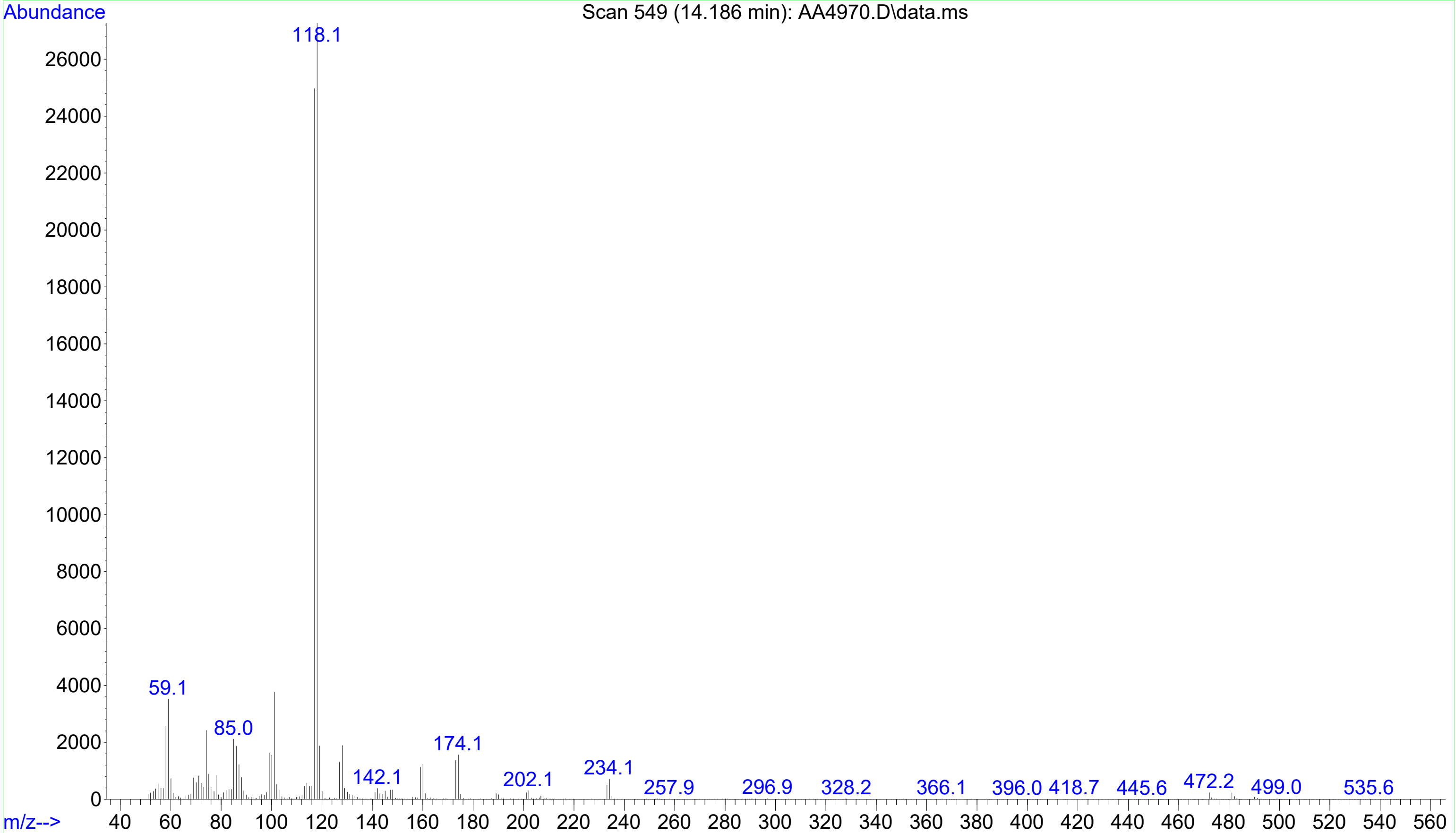

File   :E:\AA4970.D
Operator   : Artur
Acquired   : 17 Apr 2021   14:19   using AcqMethod SERVLABPMAA10TO1.M
Instrument :   GC-MS AA
Sample Name: L.digitata Laminarin
Misc Info   :
Vial Number: 44

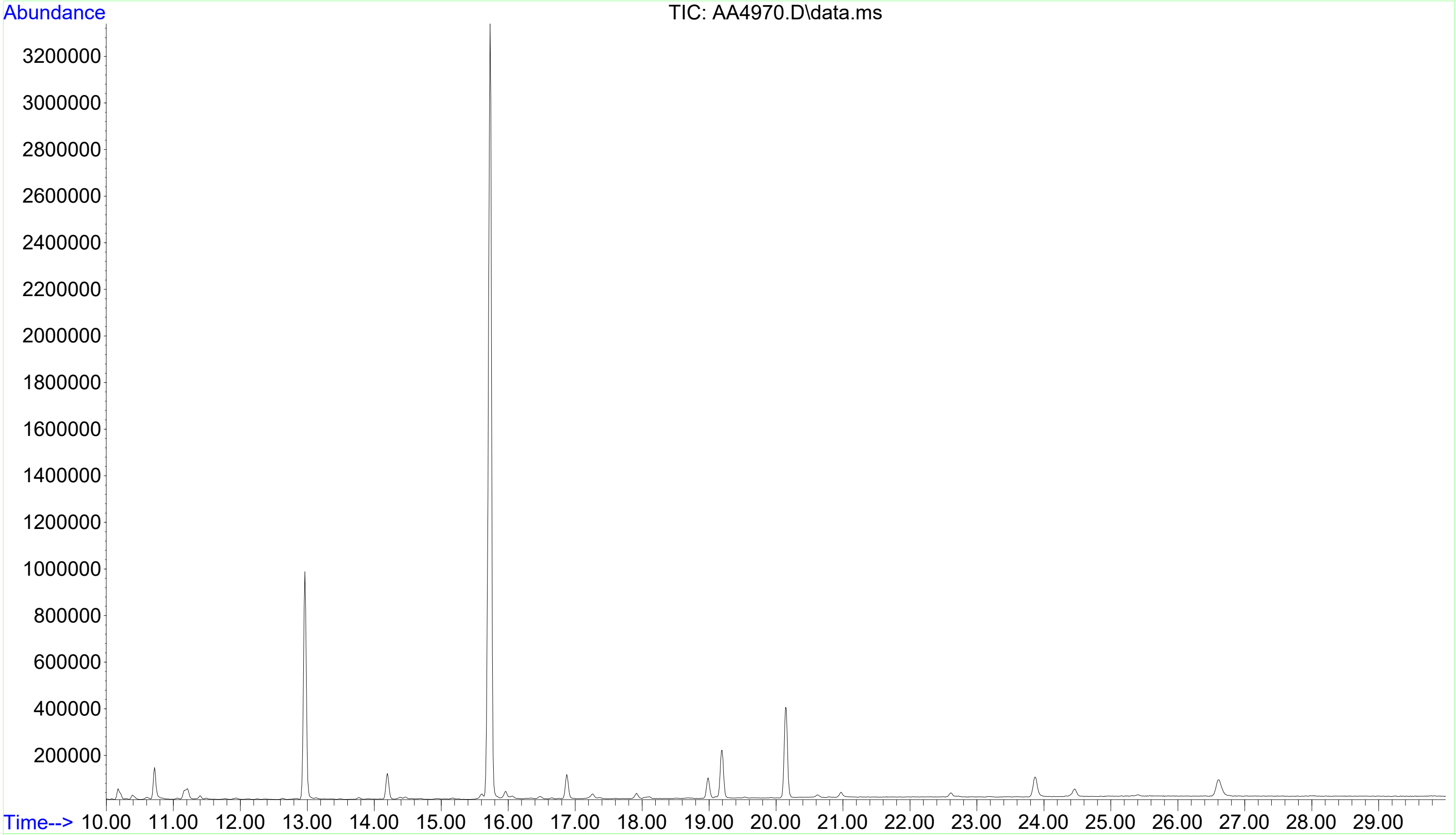

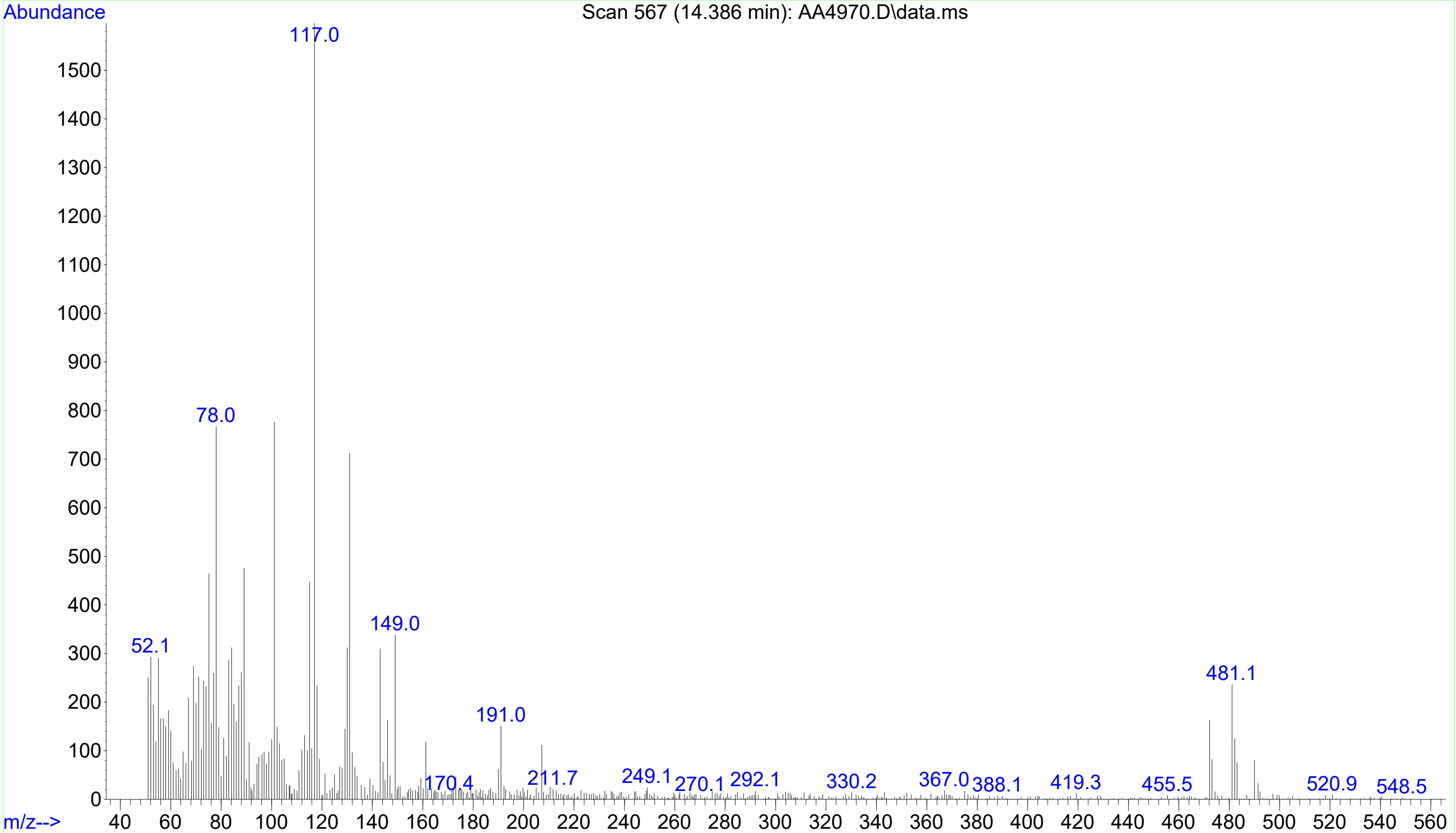

File   :E:\AA4970.D
Operator   : Artur
Acquired   : 17 Apr 2021   14:19   using AcqMethod SERVLABPMAA10TO1.M
Instrument :   GC-MS AA
Sample Name: L.digitata Laminarin
Misc Info   :
Vial Number: 44

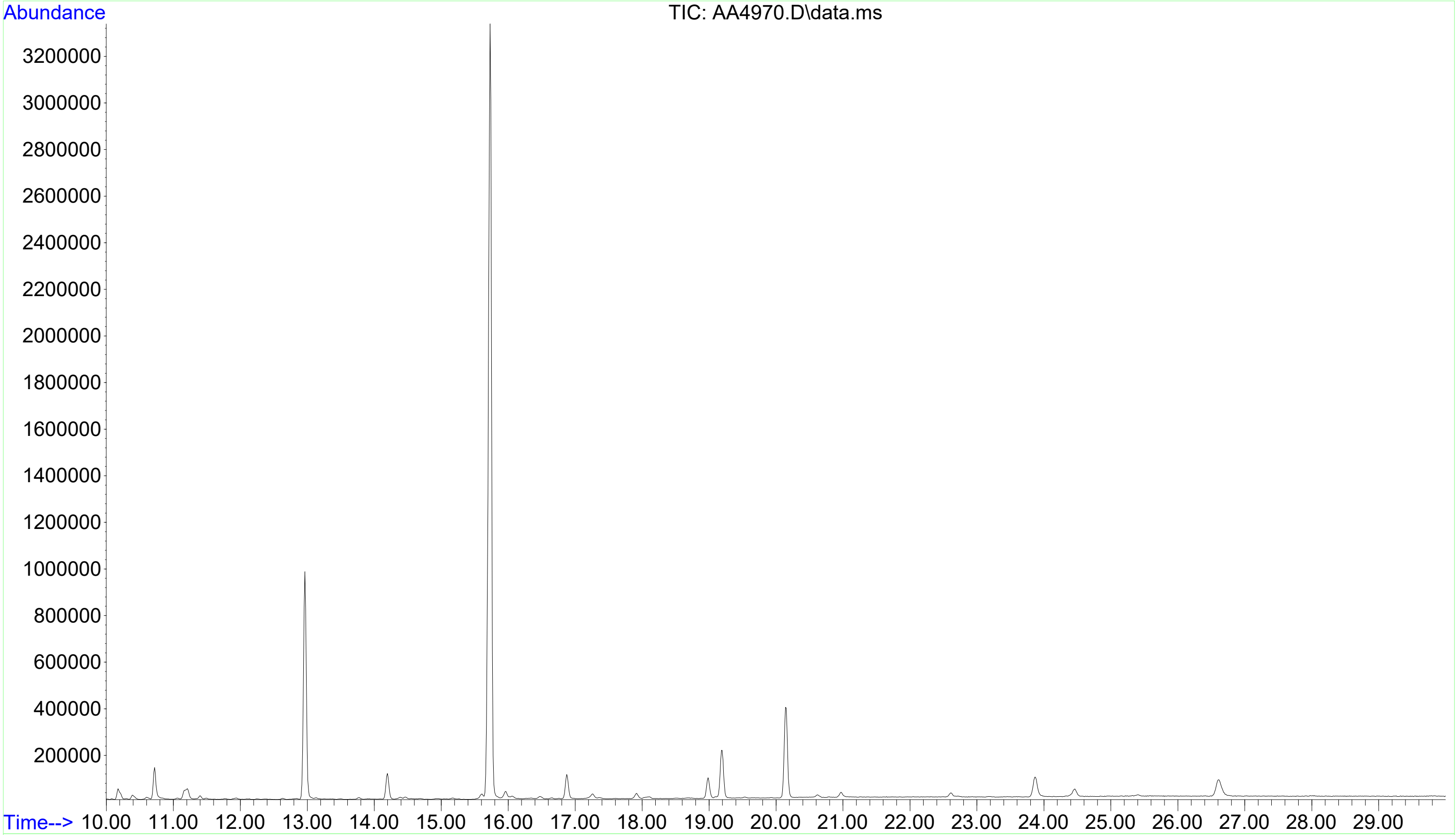

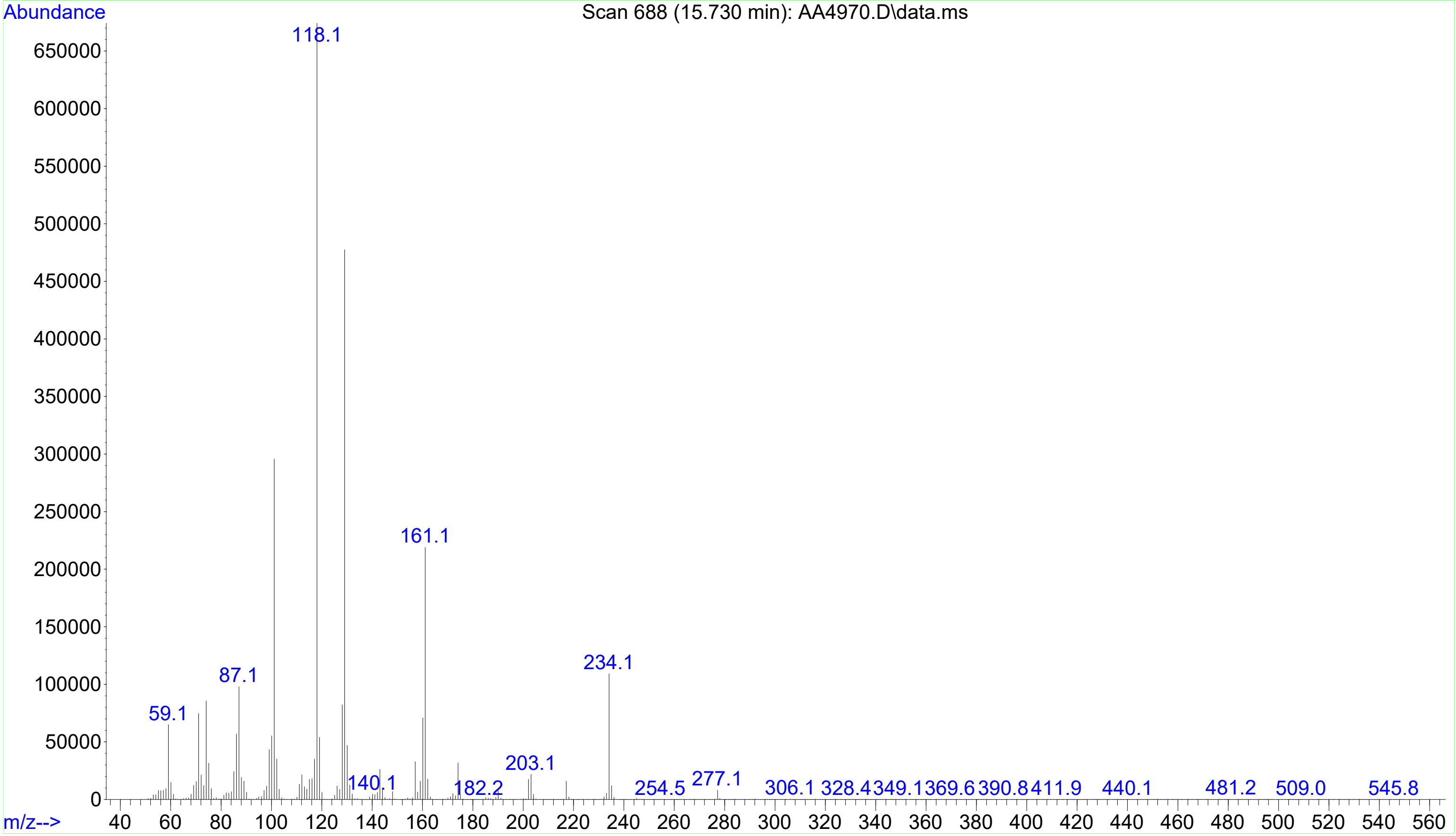

```
File        :E:\AA4970.D
Operator    : Artur
Acquired    : 17 Apr 2021   14:19     using AcqMethod SERVLABPMAA10TO1.M
Instrument :     GC-MS AA
Sample Name: L.digitata Laminarin
Misc Info   :
Vial Number: 44
```

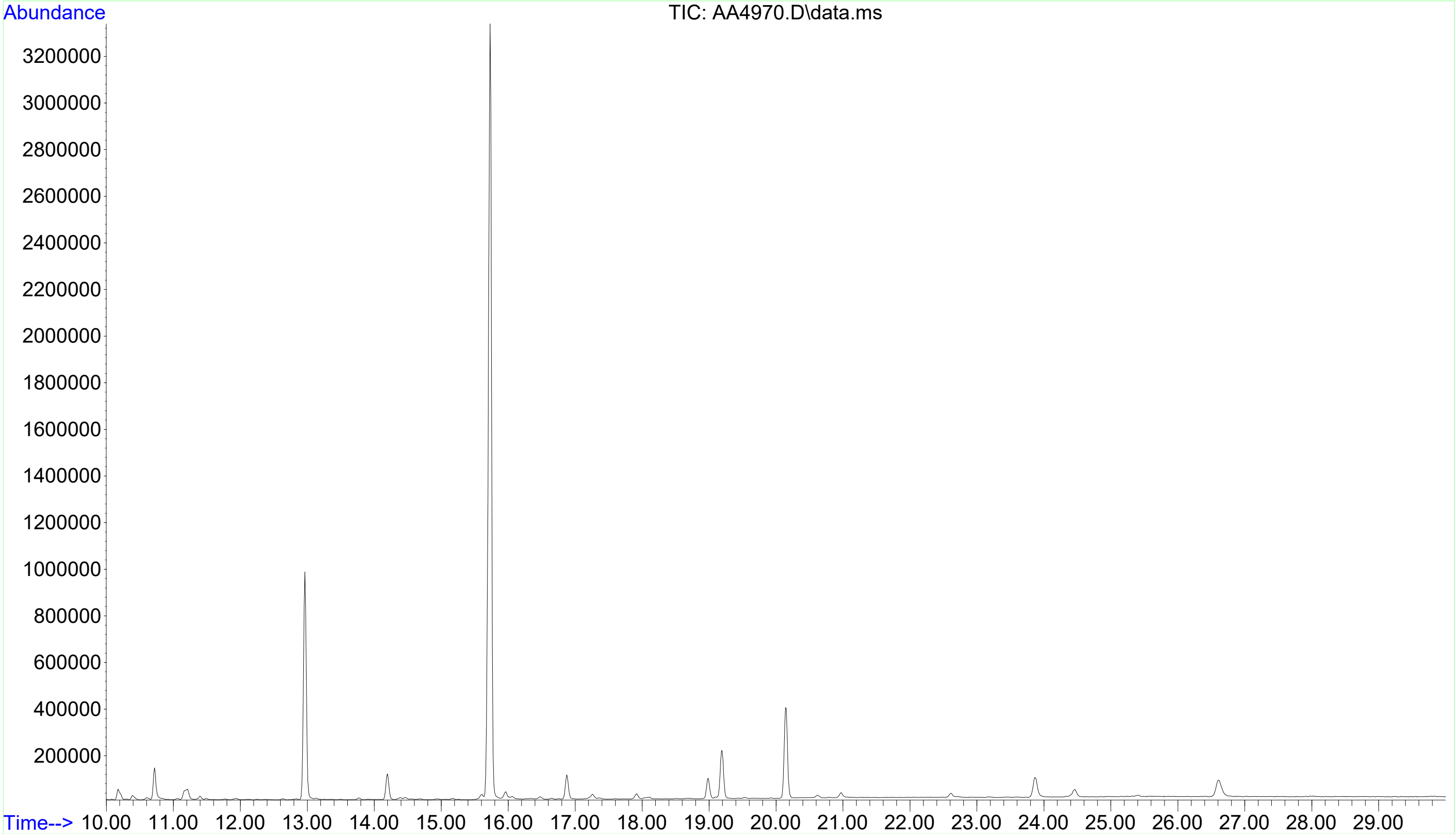

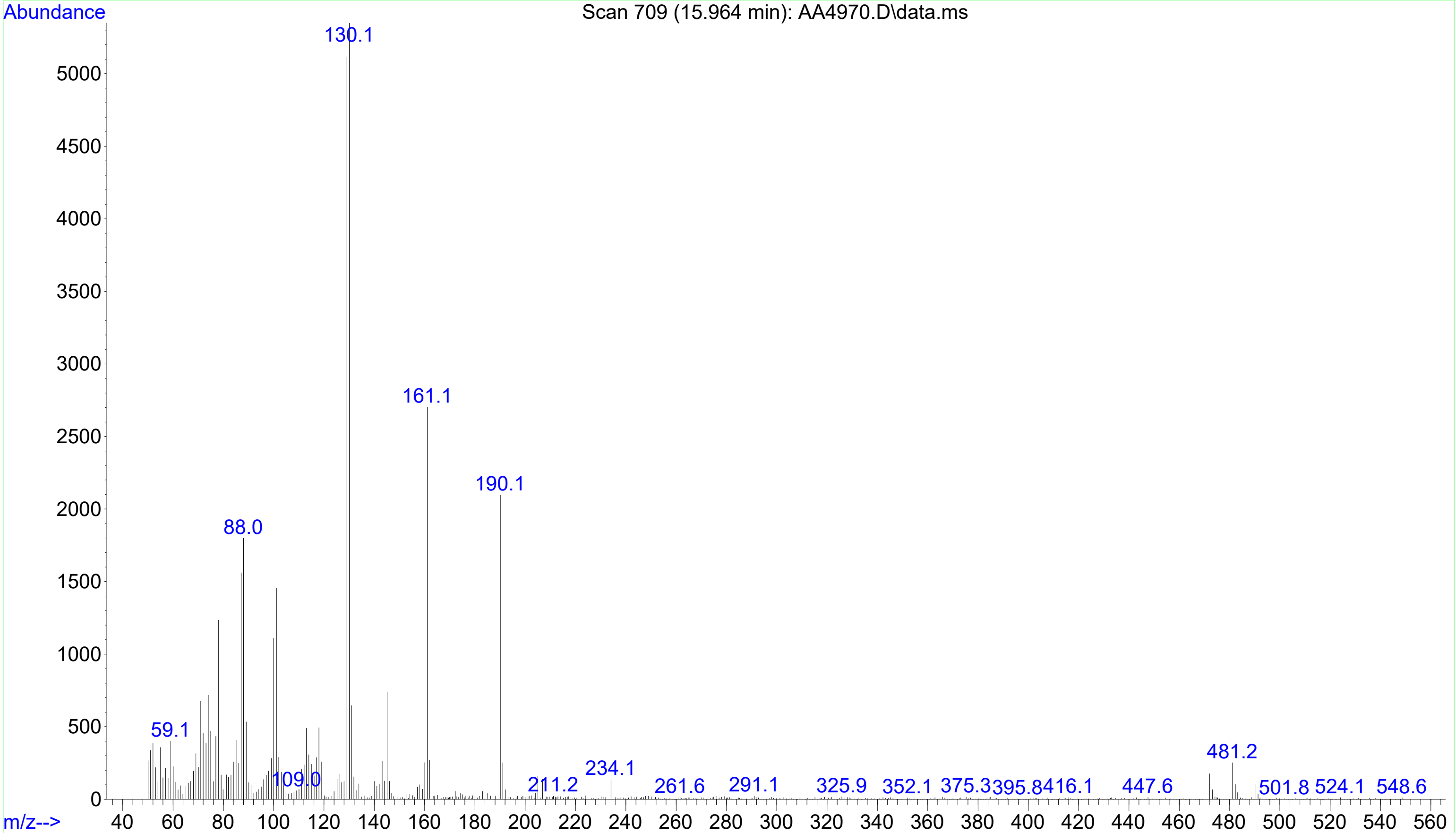

File   :E:\AA4970.D
Operator   : Artur
Acquired   : 17 Apr 2021   14:19   using AcqMethod SERVLABPMAA10TO1.M
Instrument :   GC-MS AA
Sample Name: L.digitata Laminarin
Misc Info   :
Vial Number: 44

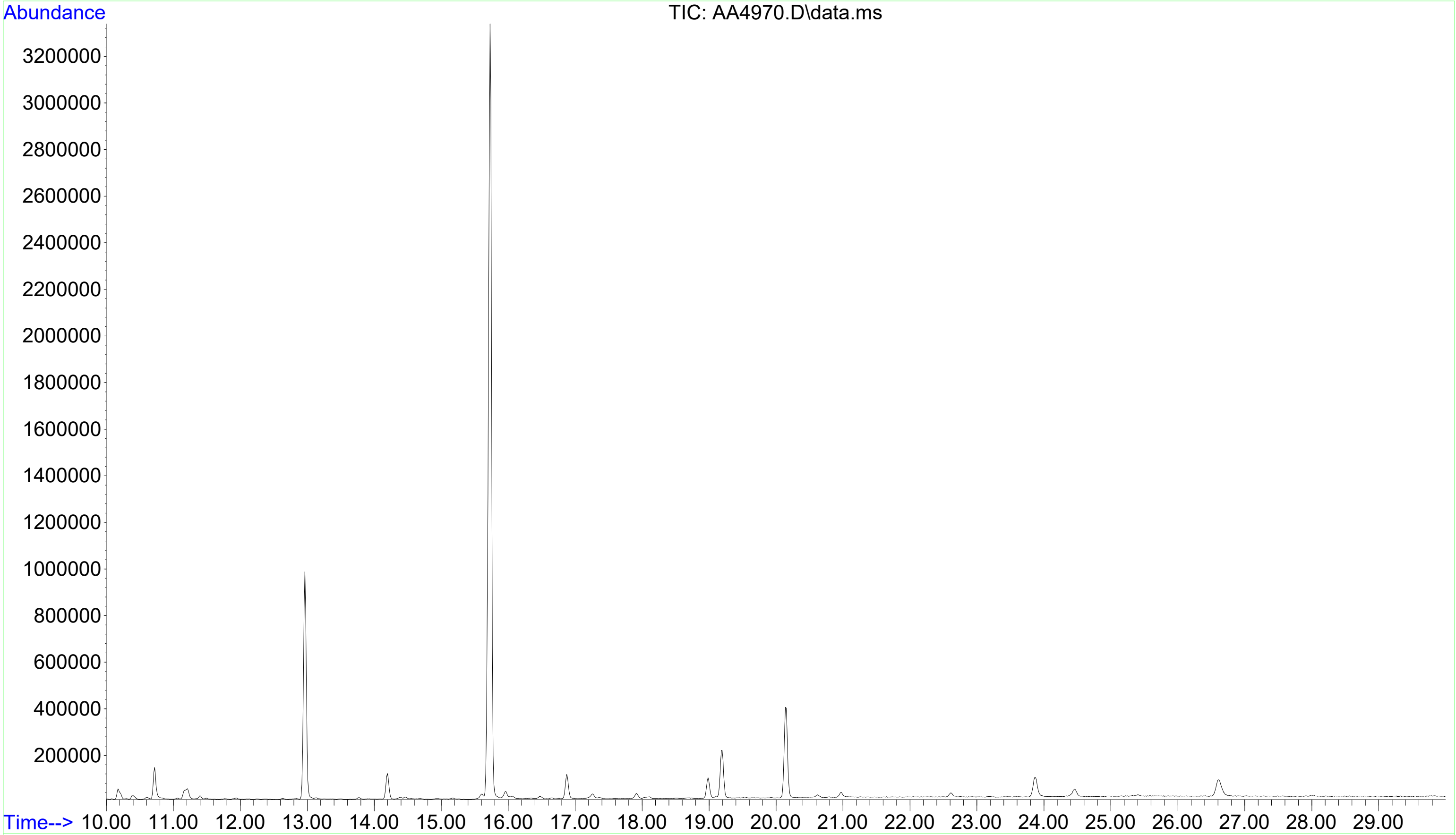

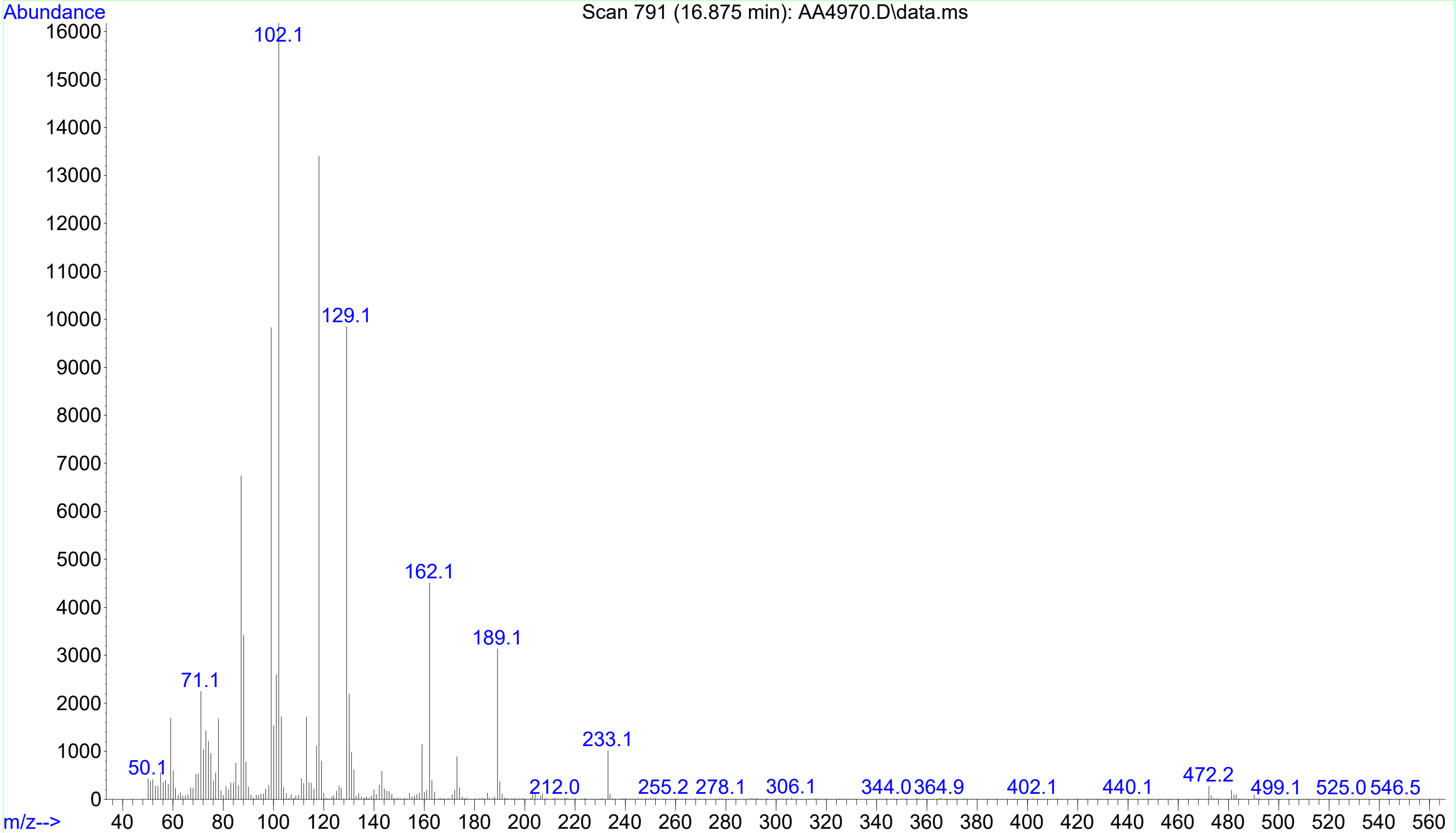

File   :E:\AA4970.D
Operator   : Artur
Acquired   : 17 Apr 2021   14:19   using AcqMethod SERVLABPMAA10TO1.M
Instrument :   GC-MS AA
Sample Name: L.digitata Laminarin
Misc Info   :
Vial Number: 44

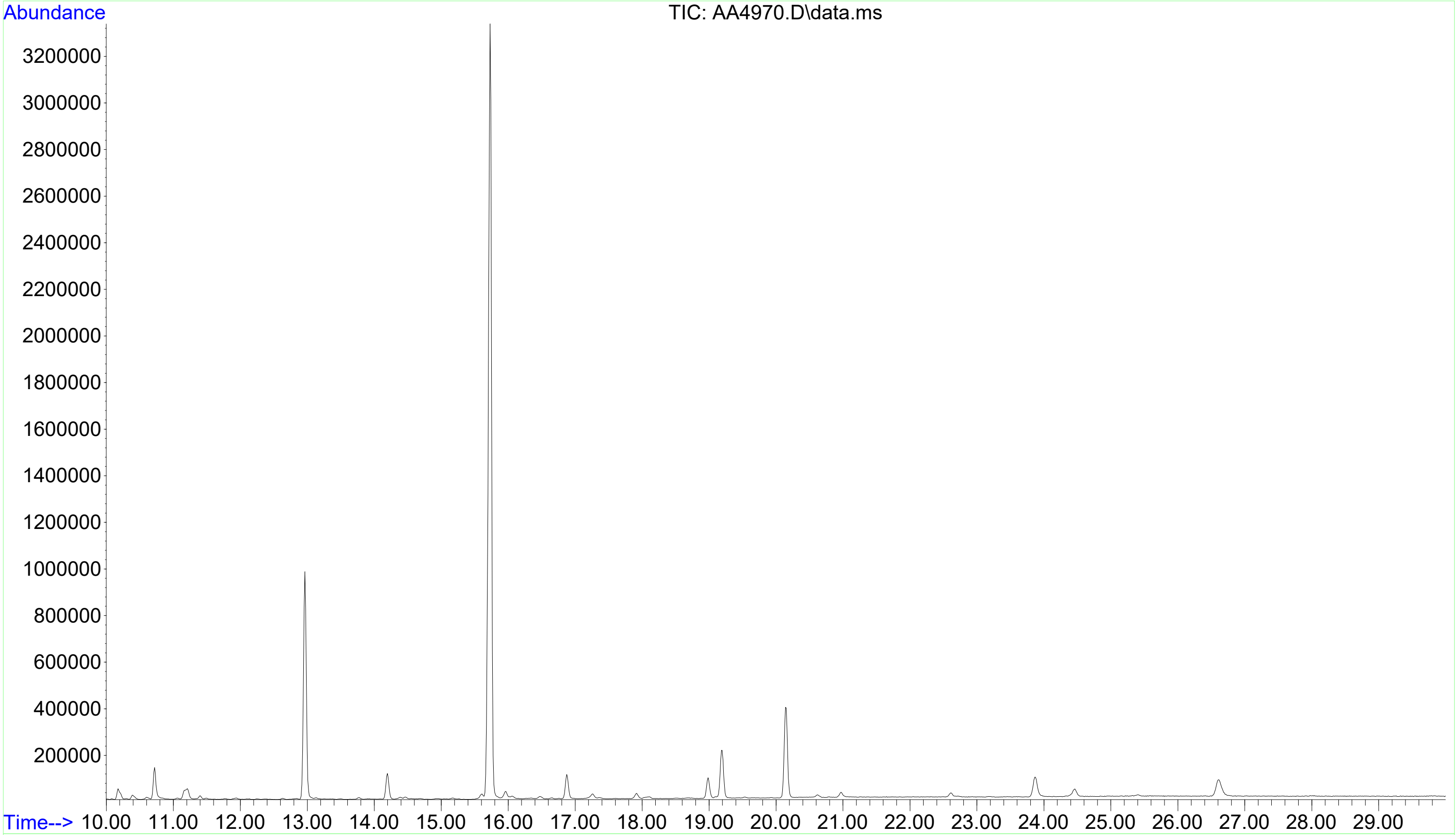

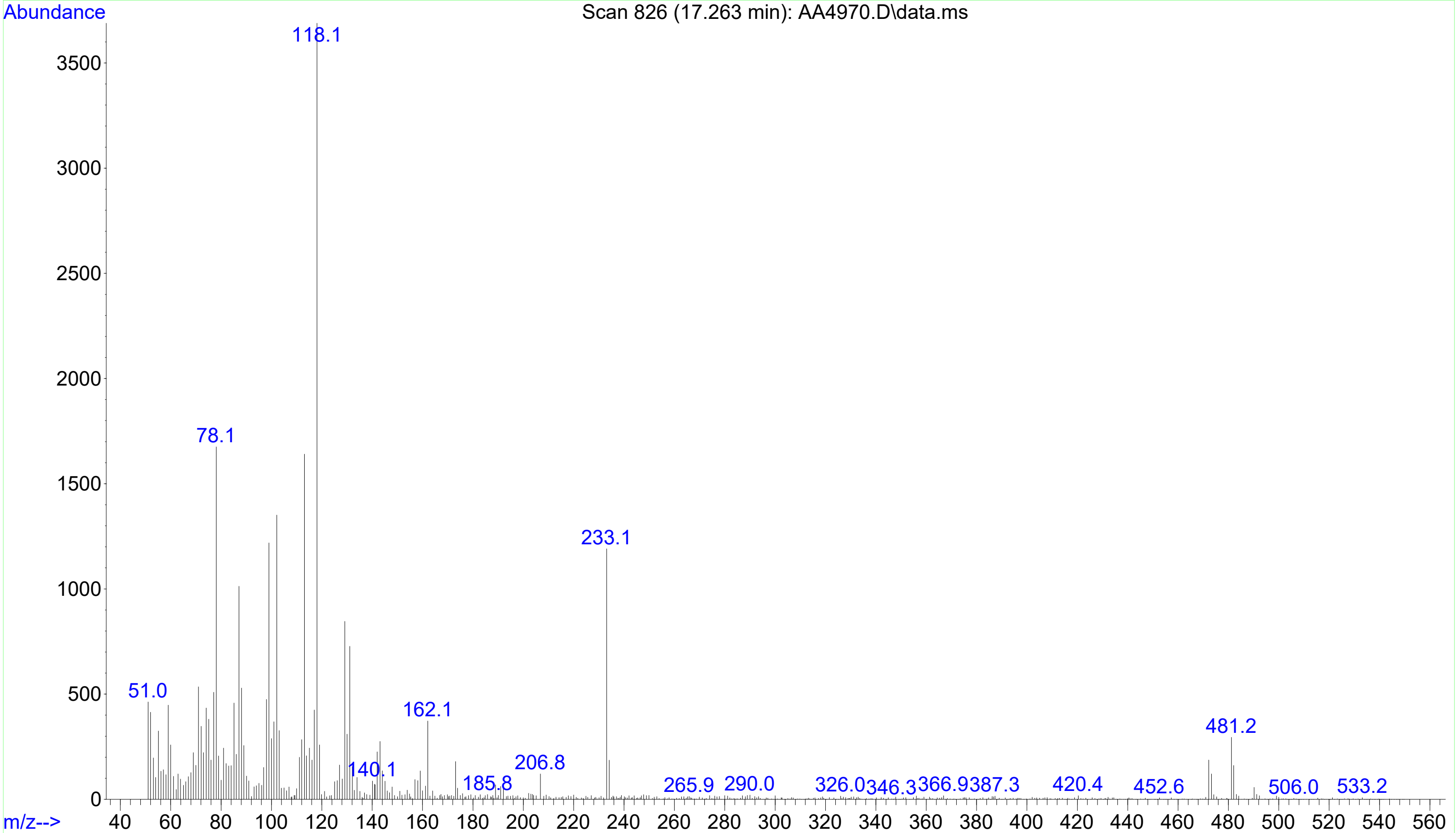

```
File        :E:\AA4970.D
Operator    : Artur
Acquired    : 17 Apr 2021   14:19      using AcqMethod SERVLABPMAA10TO1.M
Instrument :     GC-MS AA
Sample Name: L.digitata Laminarin
Misc Info   :
Vial Number: 44
```

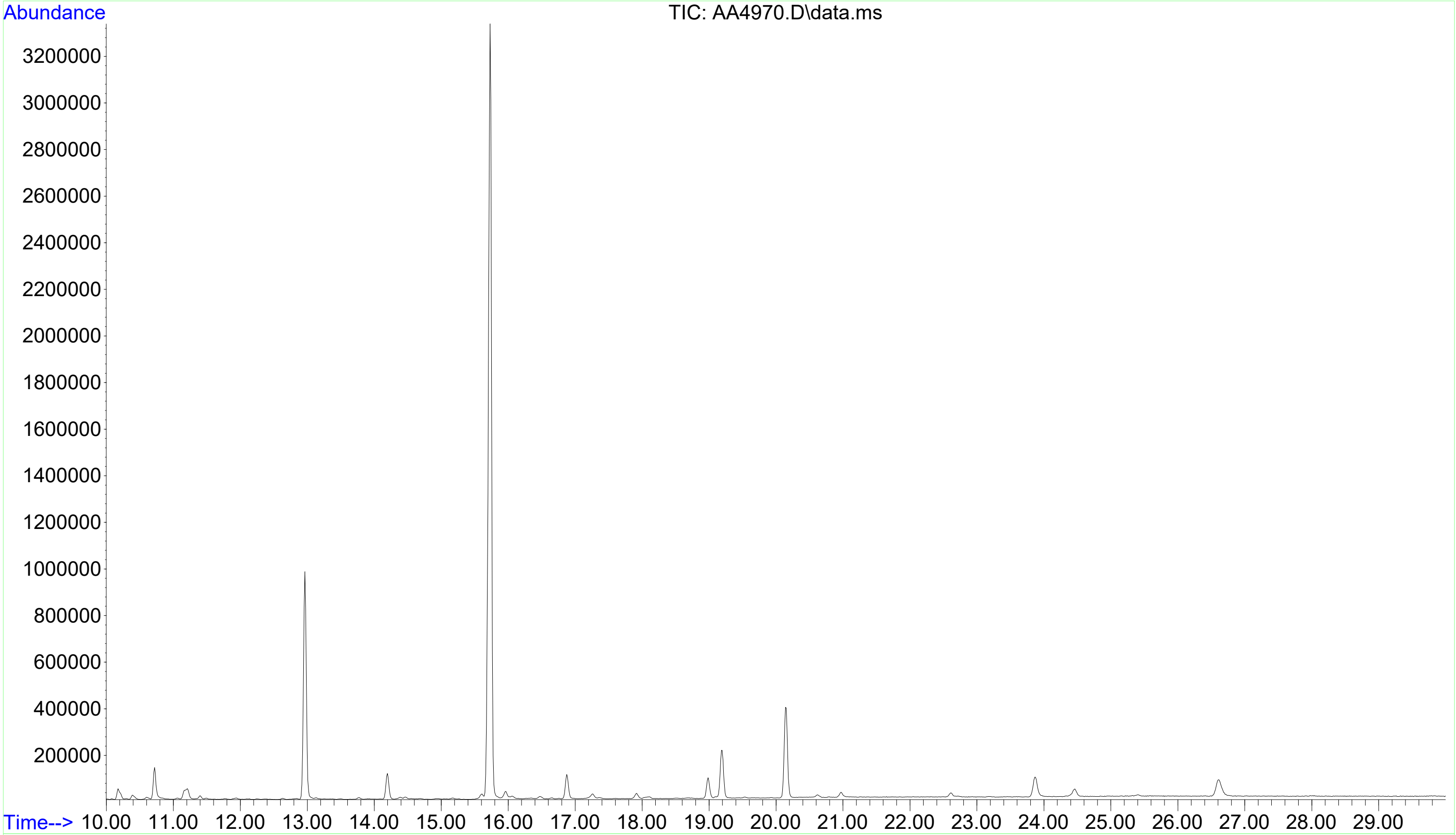

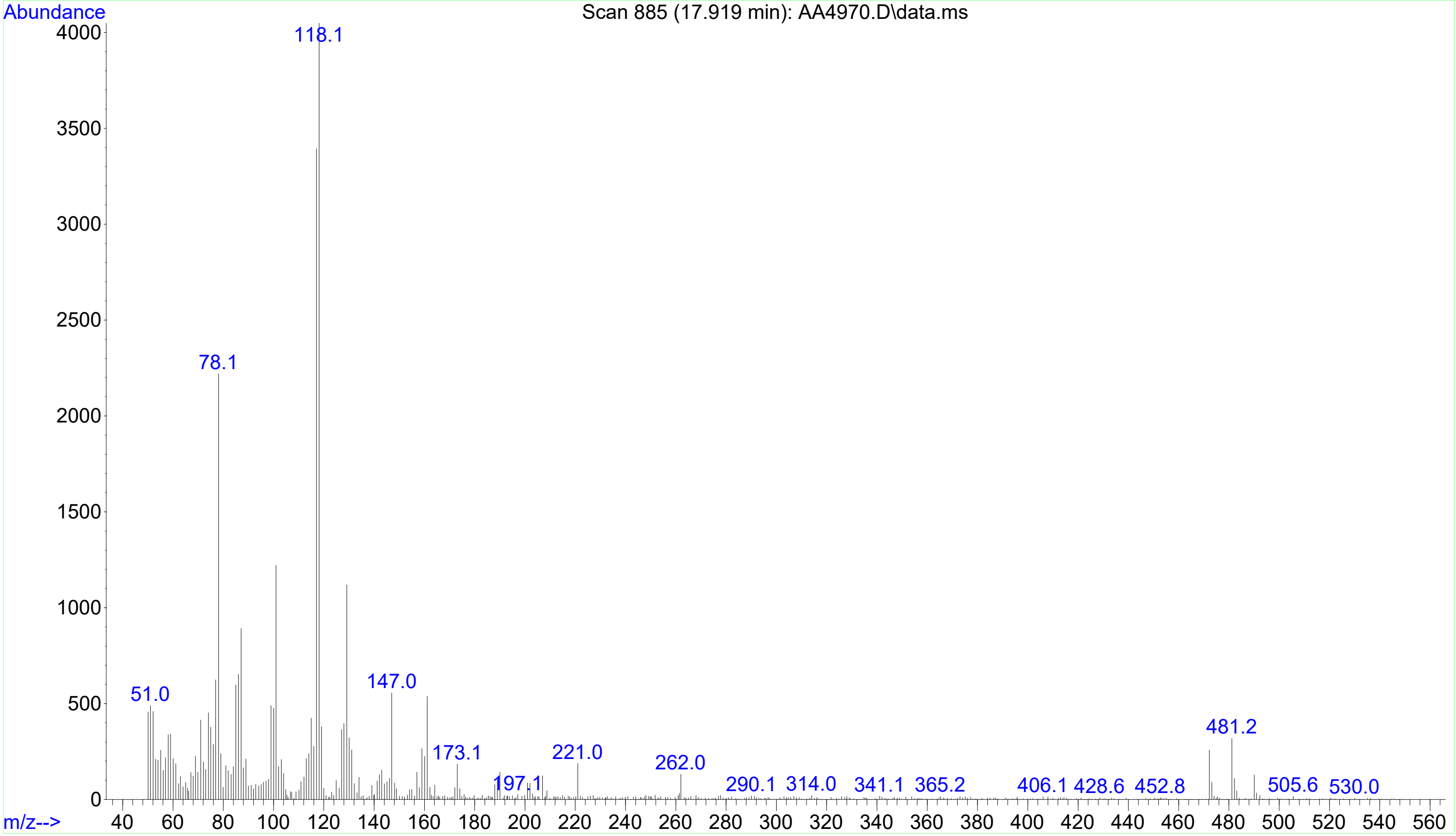

File   :E:\AA4970.D
Operator   : Artur
Acquired   : 17 Apr 2021   14:19   using AcqMethod SERVLABPMAA10TO1.M
Instrument :   GC-MS AA
Sample Name: L.digitata Laminarin
Misc Info   :
Vial Number: 44

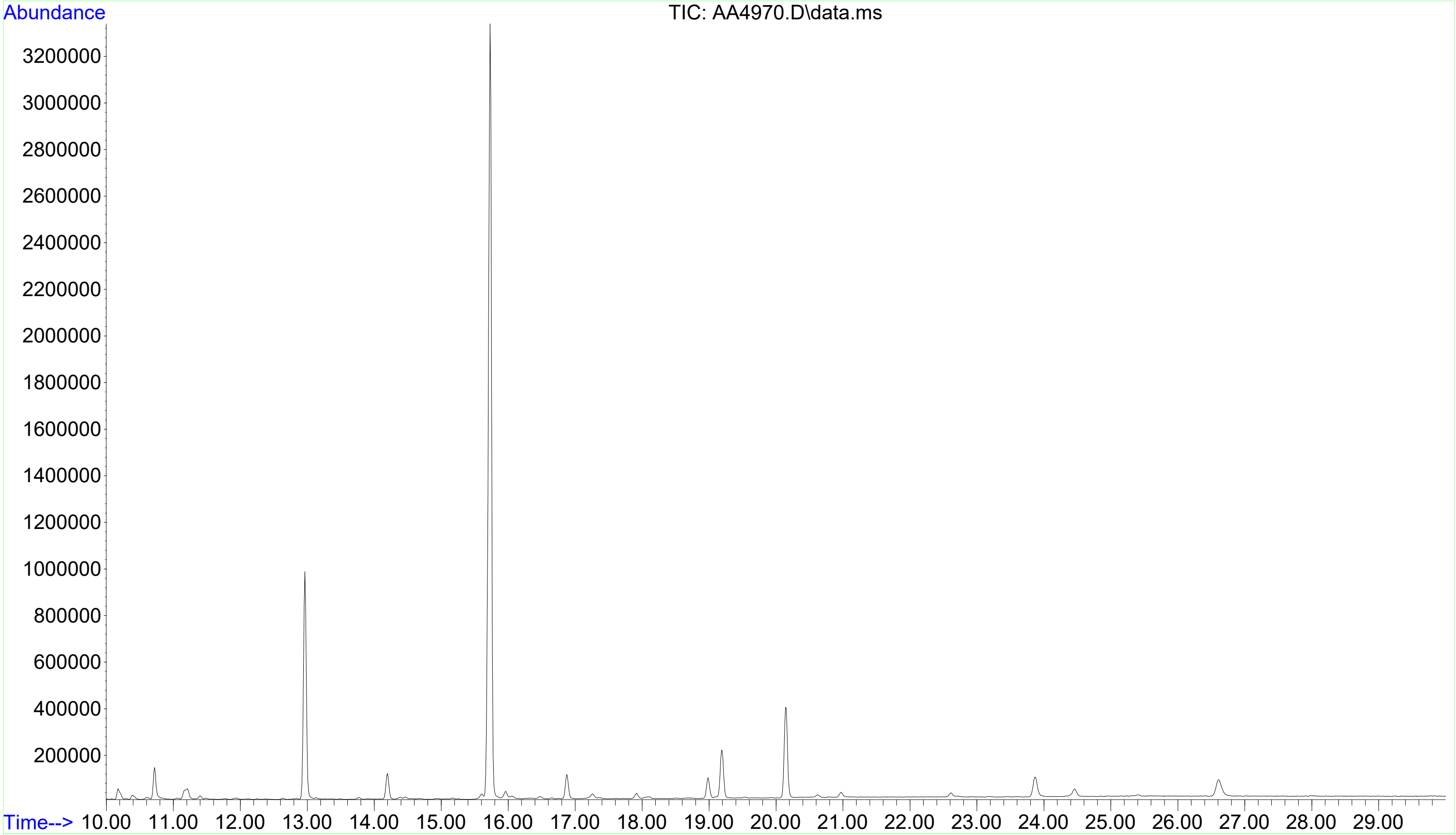

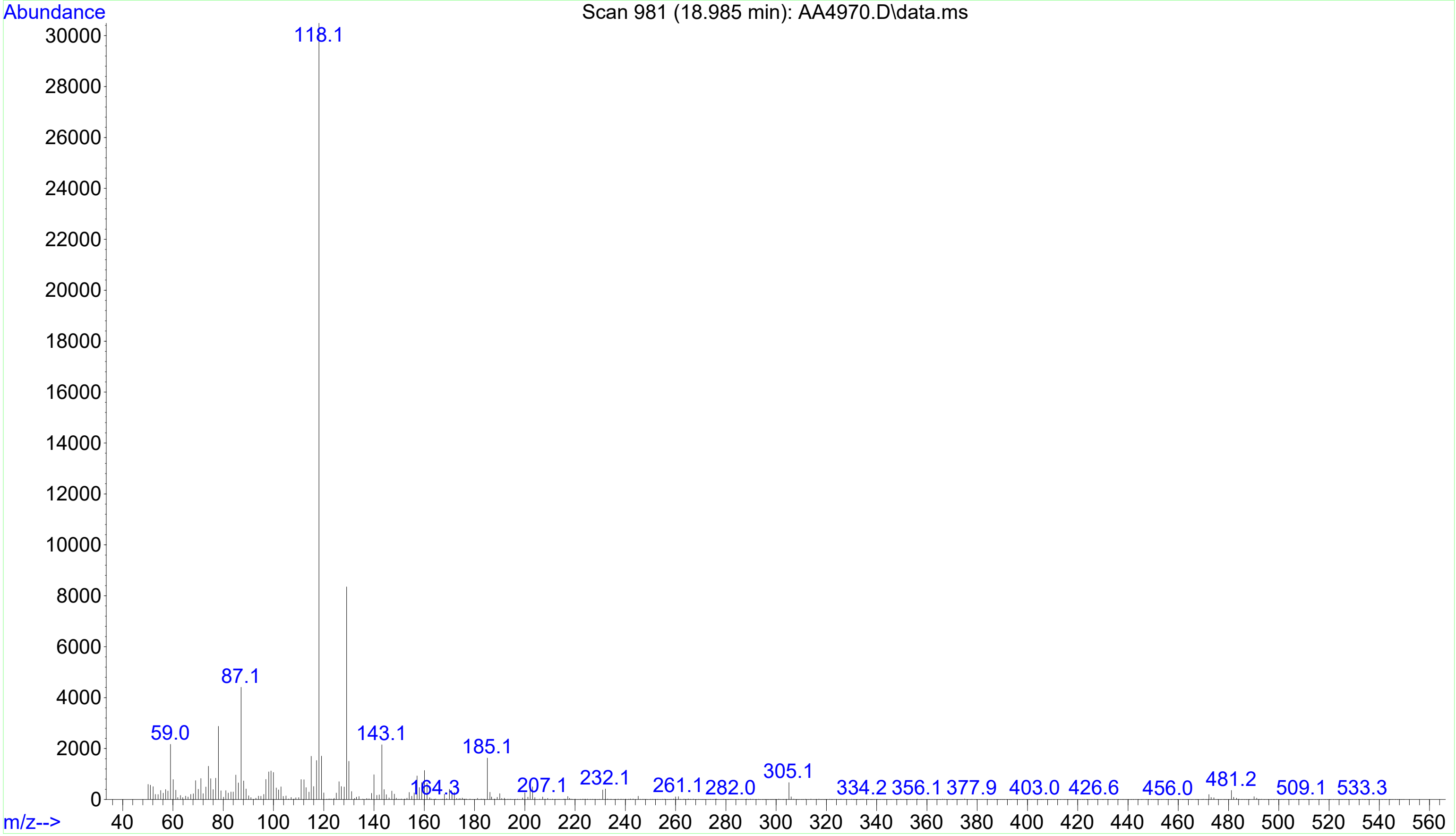

File   :E:\AA4970.D
Operator   : Artur
Acquired   : 17 Apr 2021   14:19   using AcqMethod SERVLABPMAA10TO1.M
Instrument :   GC-MS AA
Sample Name: L.digitata Laminarin
Misc Info   :
Vial Number: 44

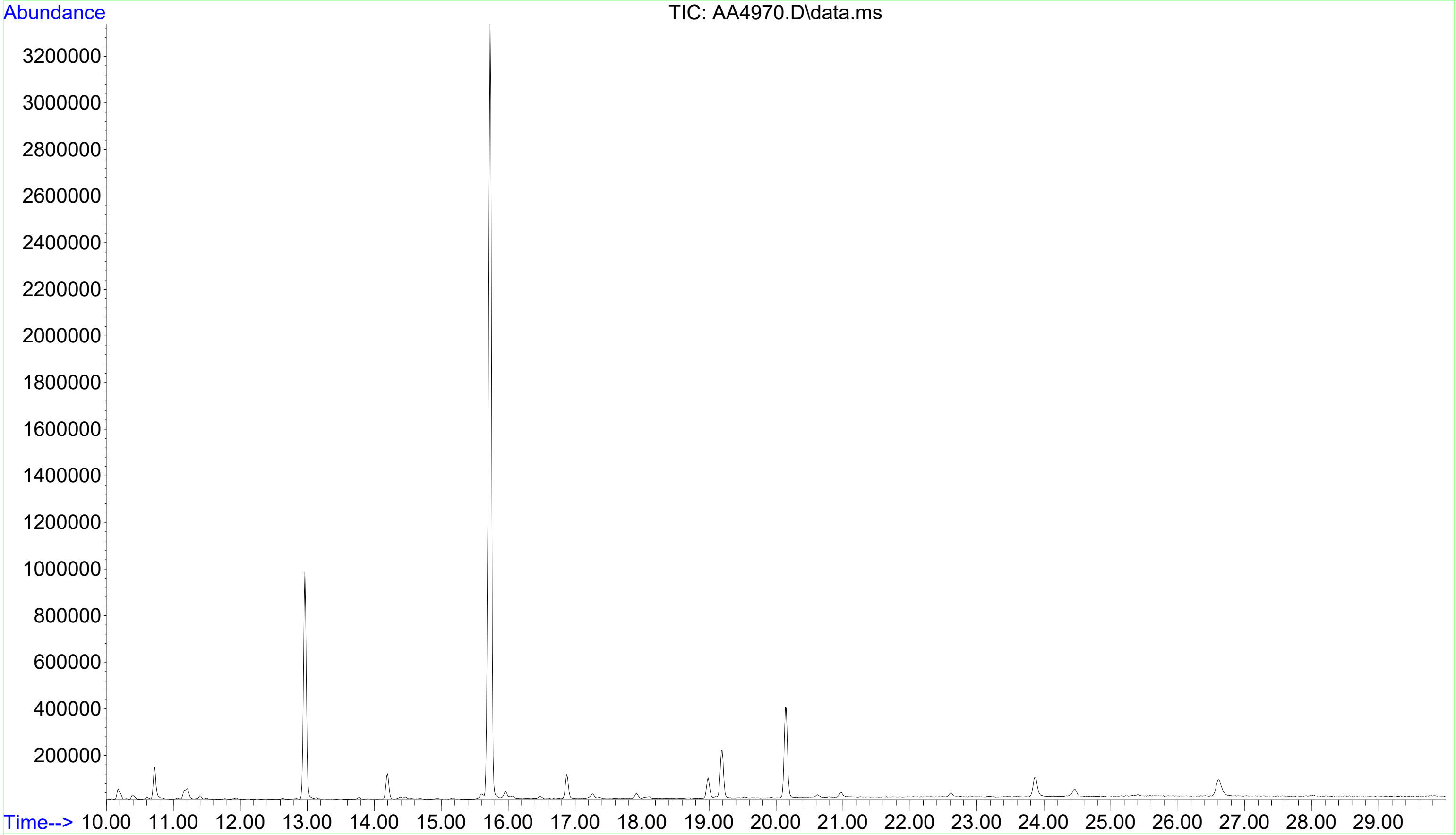

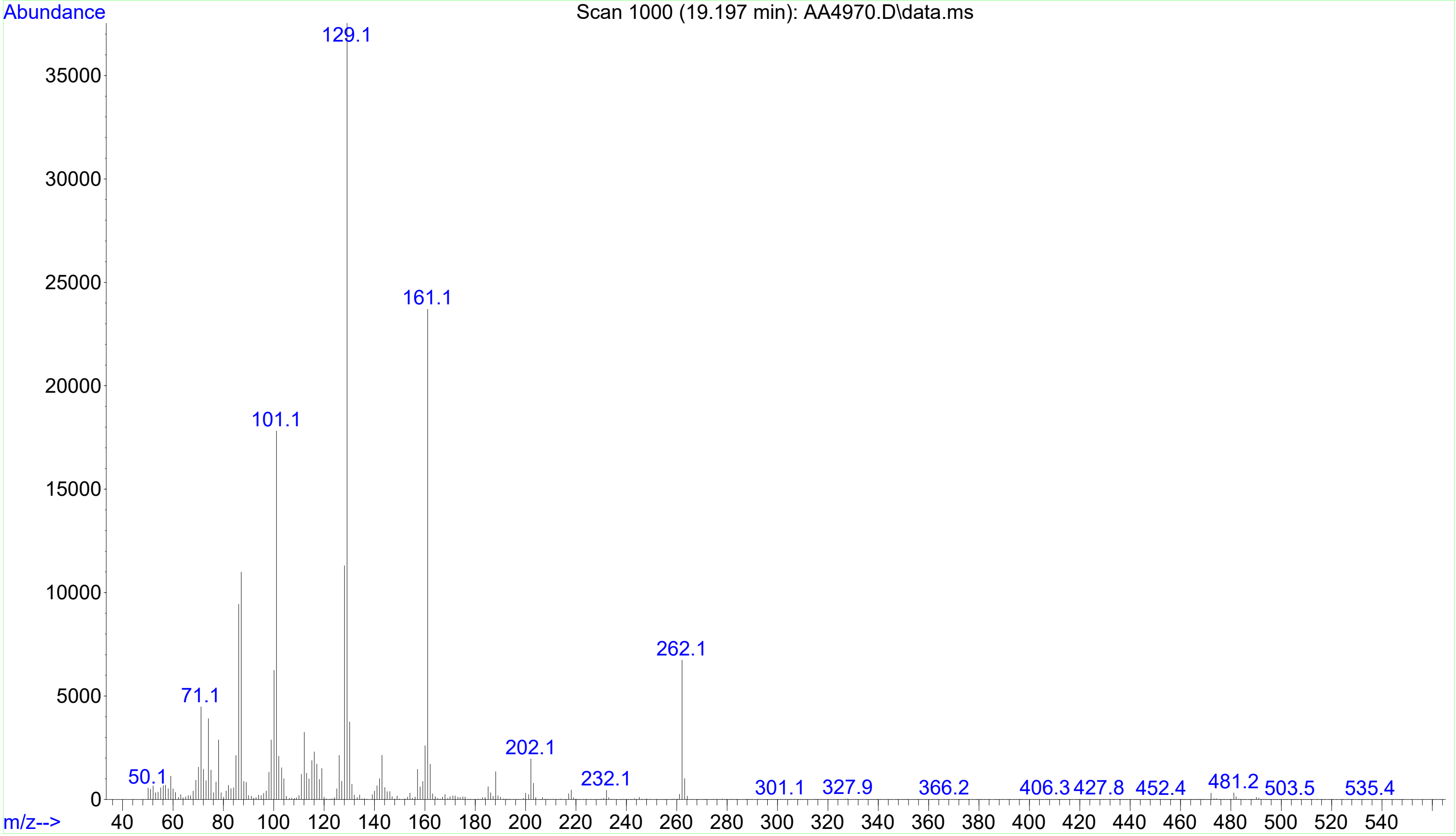

```
File        :E:\AA4970.D
Operator    : Artur
Acquired    : 17 Apr 2021  14:19      using AcqMethod SERVLABPMAA10TO1.M
Instrument  :    GC-MS AA
Sample Name: L.digitata Laminarin
Misc Info   :
Vial Number: 44
```

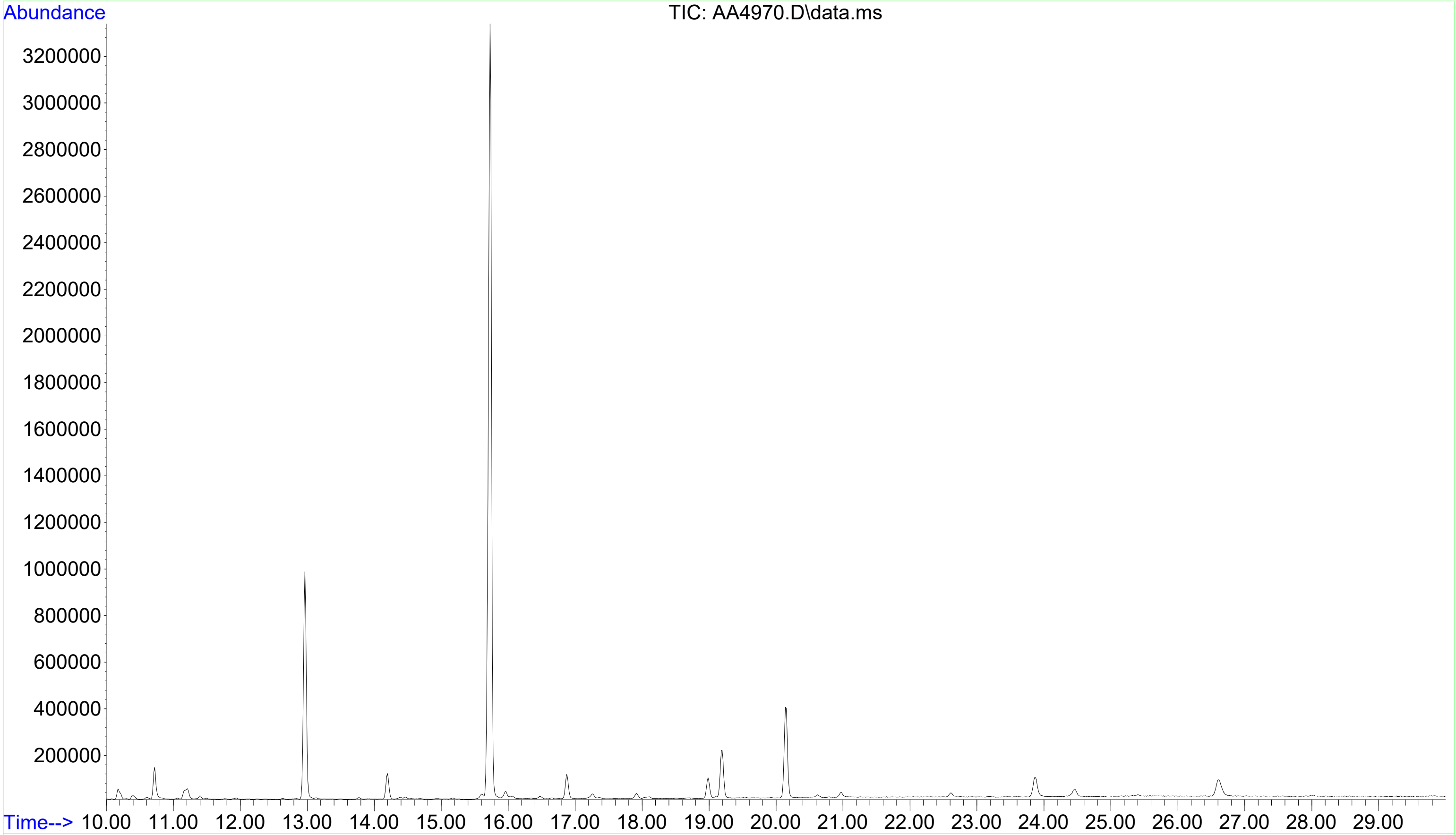

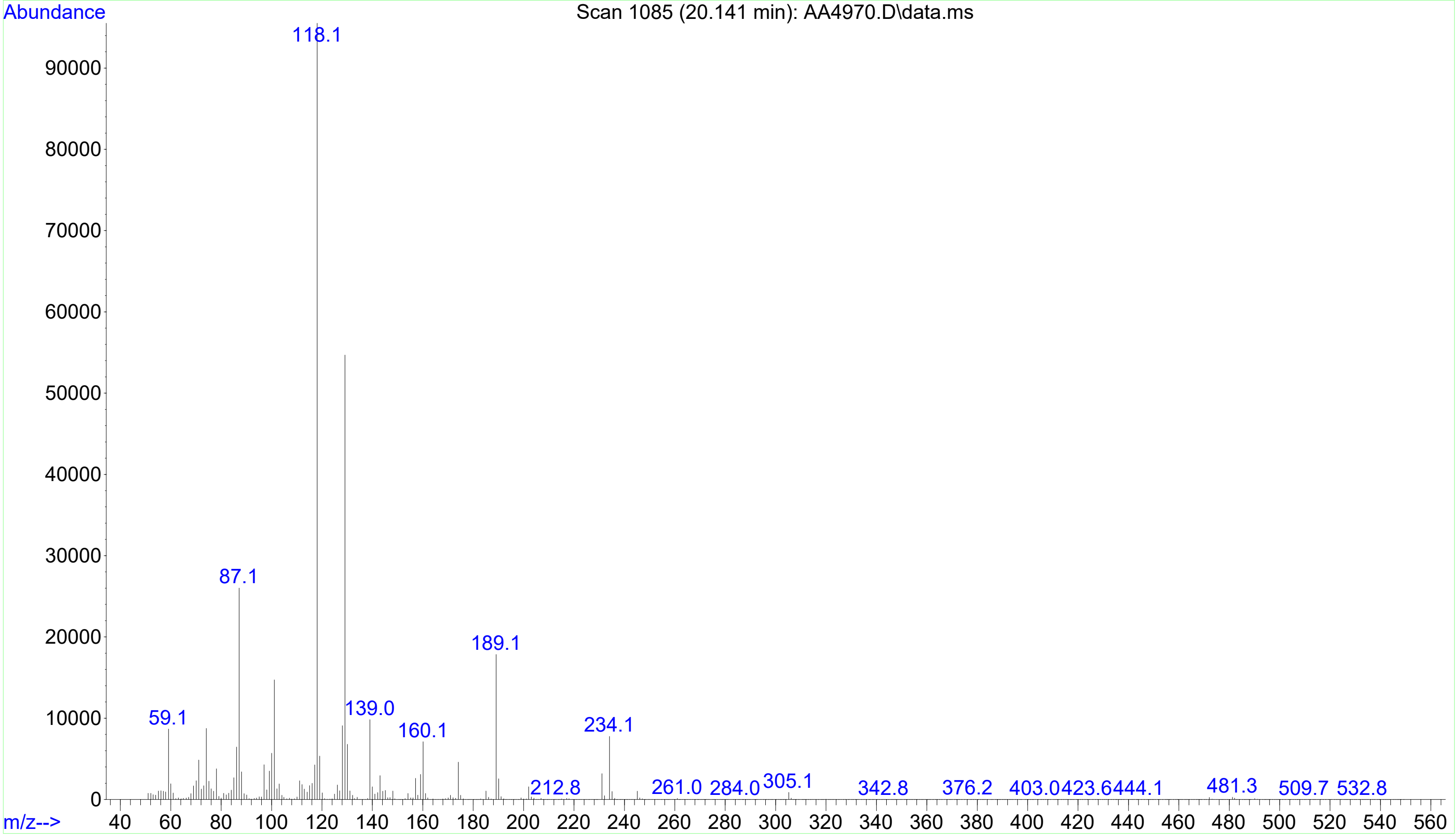

File   :E:\AA4970.D
Operator   : Artur
Acquired   : 17 Apr 2021   14:19   using AcqMethod SERVLABPMAA10TO1.M
Instrument :   GC-MS AA
Sample Name: L.digitata Laminarin
Misc Info   :
Vial Number: 44

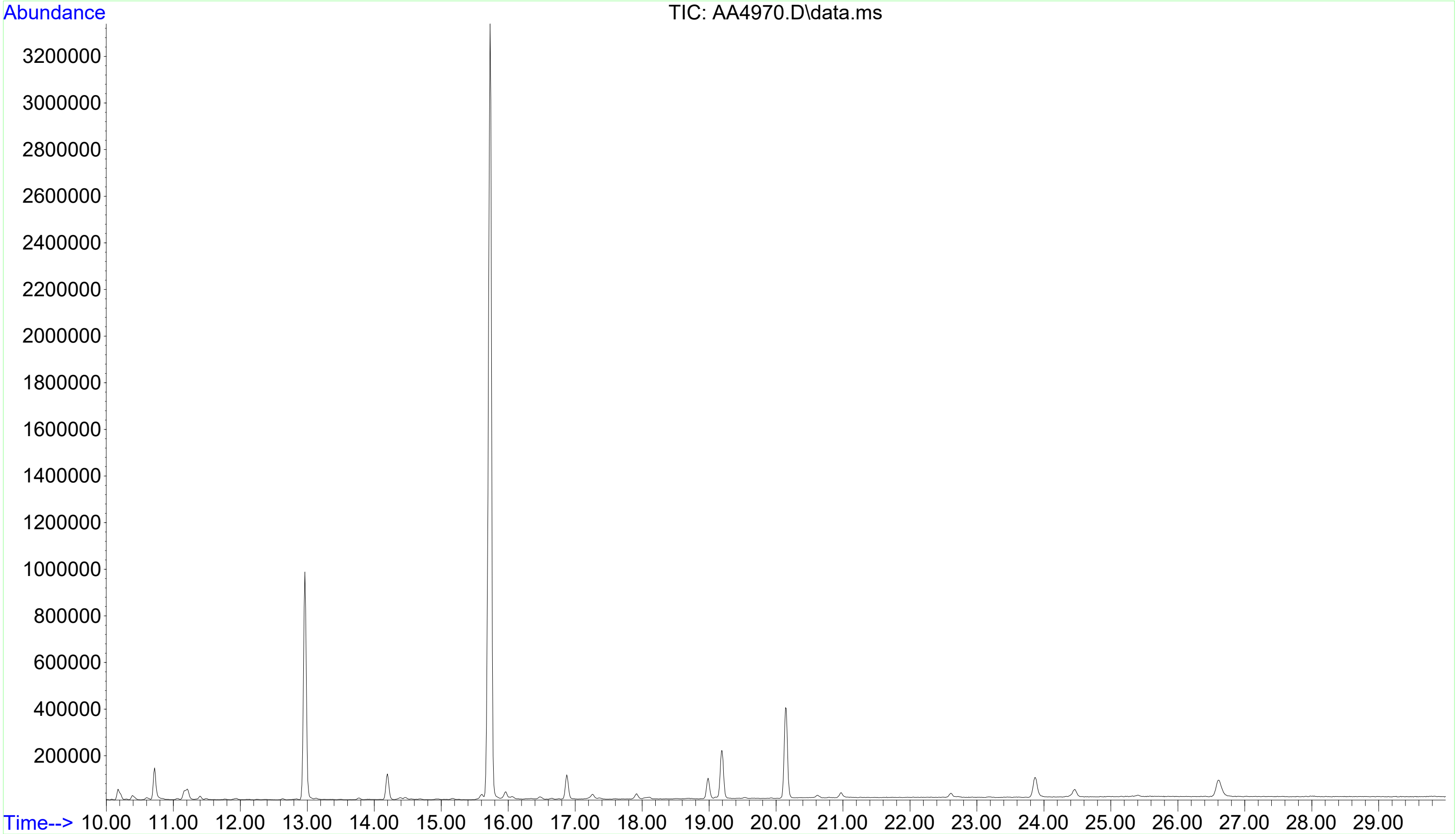

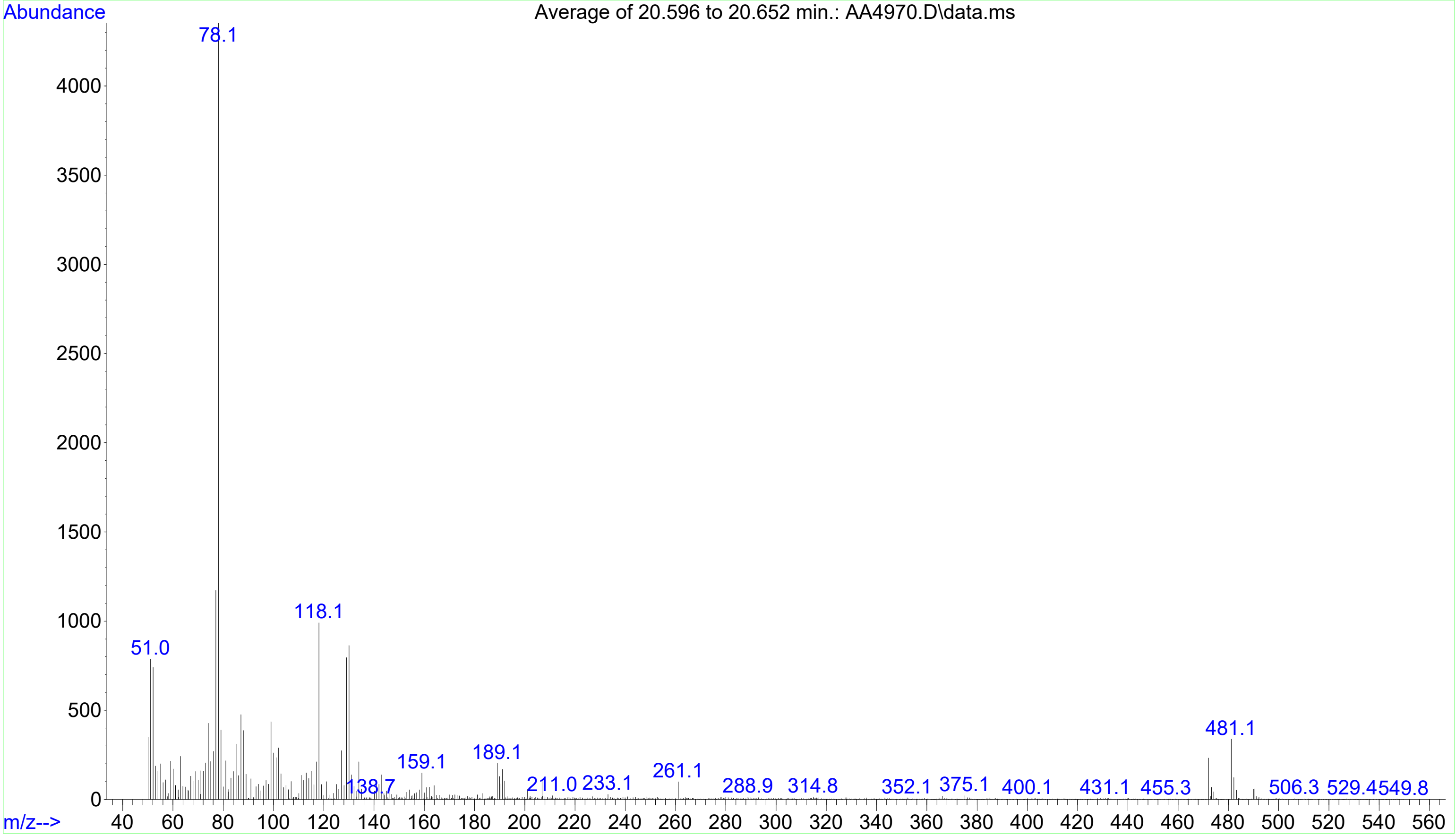

```
File        :E:\AA4970.D
Operator    : Artur
Acquired    : 17 Apr 2021   14:19      using AcqMethod SERVLABPMAA10TO1.M
Instrument  :    GC-MS AA
Sample Name: L.digitata Laminarin
Misc Info   :
Vial Number: 44
```

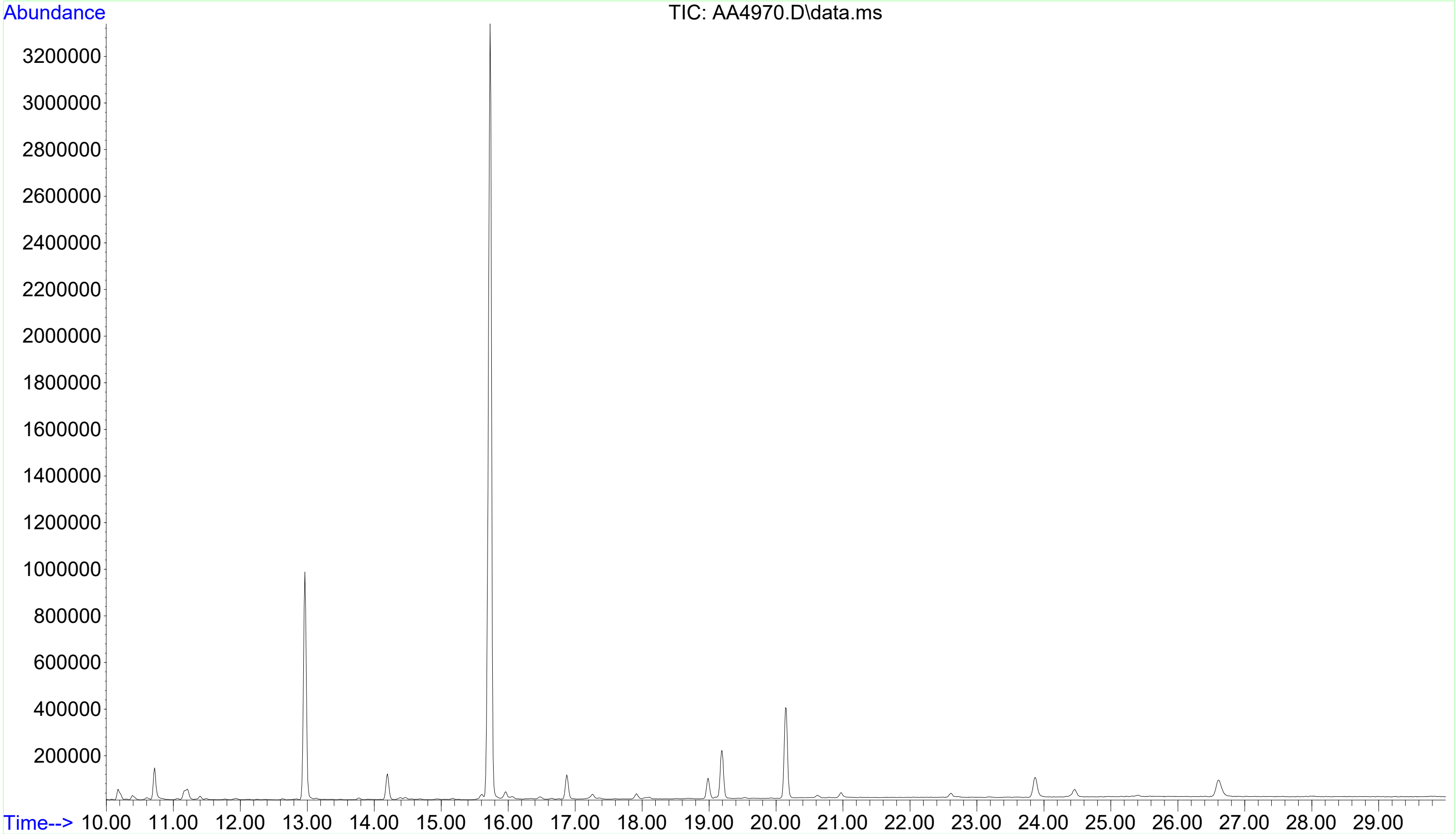

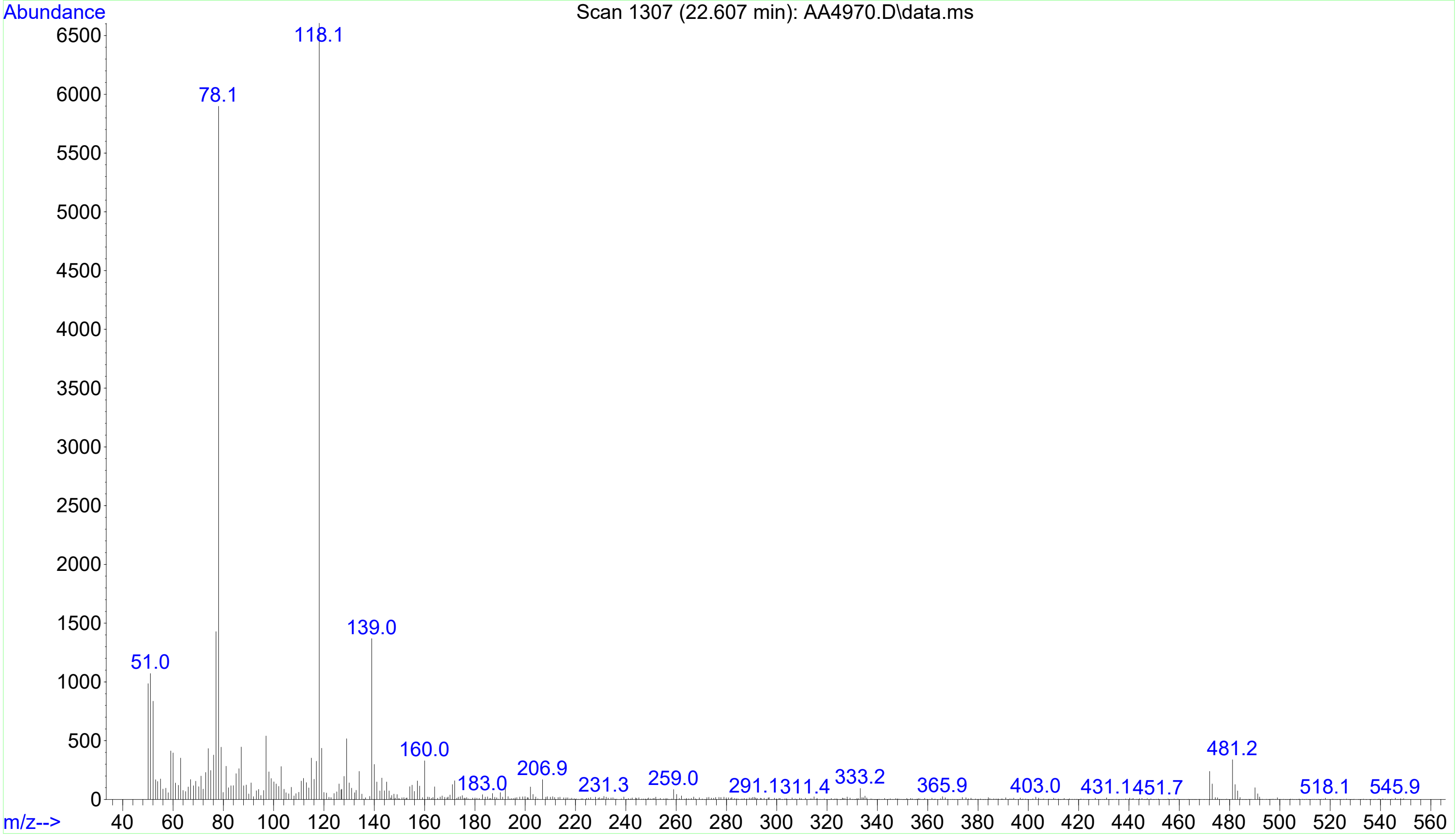

```
File        :E:\AA4970.D
Operator    : Artur
Acquired    : 17 Apr 2021   14:19     using AcqMethod SERVLABPMAA10TO1.M
Instrument :    GC-MS AA
Sample Name: L.digitata Laminarin
Misc Info   :
Vial Number: 44
```

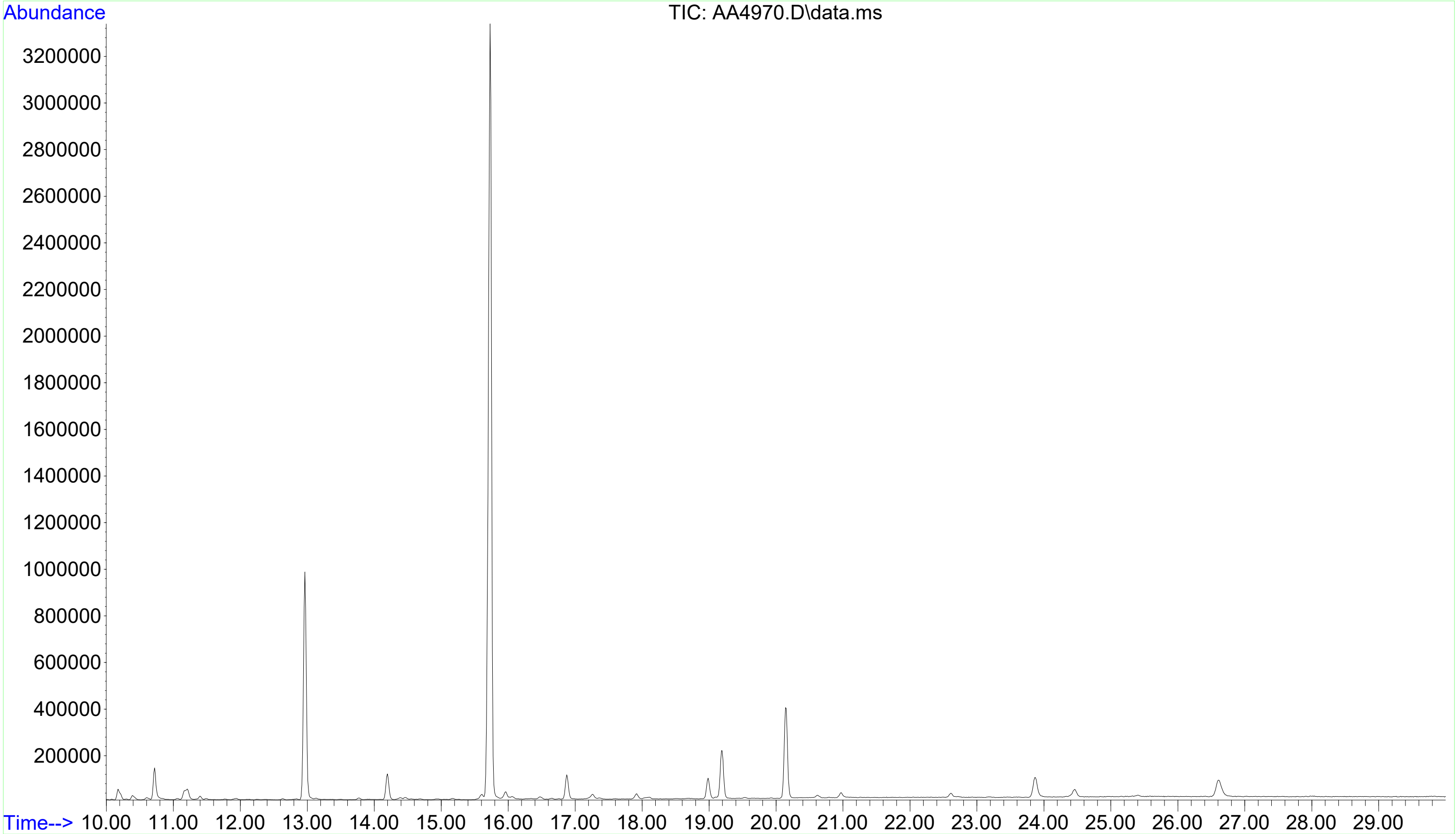

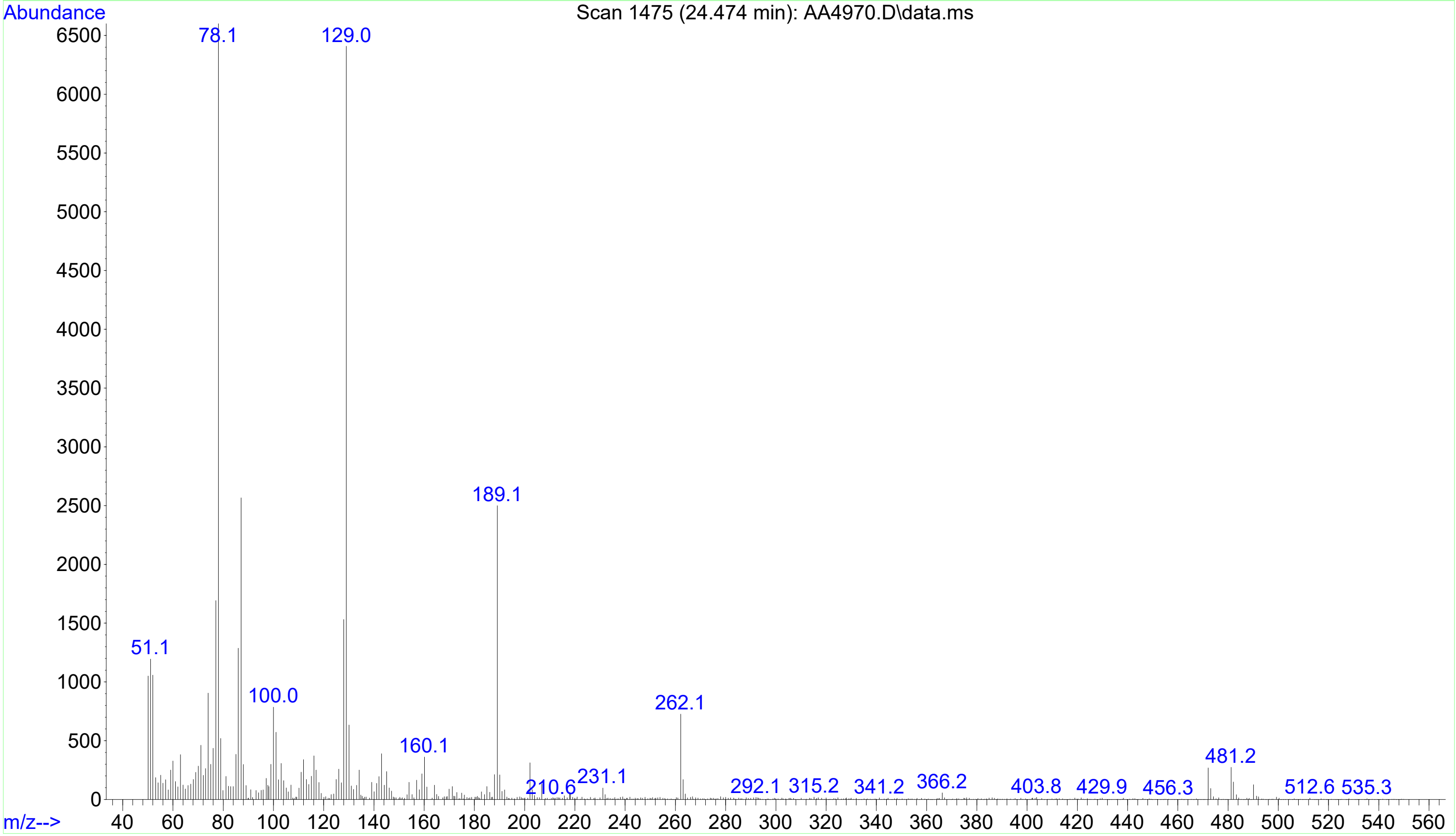

```
File        :E:\AA4970.D
Operator    : Artur
Acquired    : 17 Apr 2021   14:19      using AcqMethod SERVLABPMAA10TO1.M
Instrument :     GC-MS AA
Sample Name: L.digitata Laminarin
Misc Info   :
Vial Number: 44
```

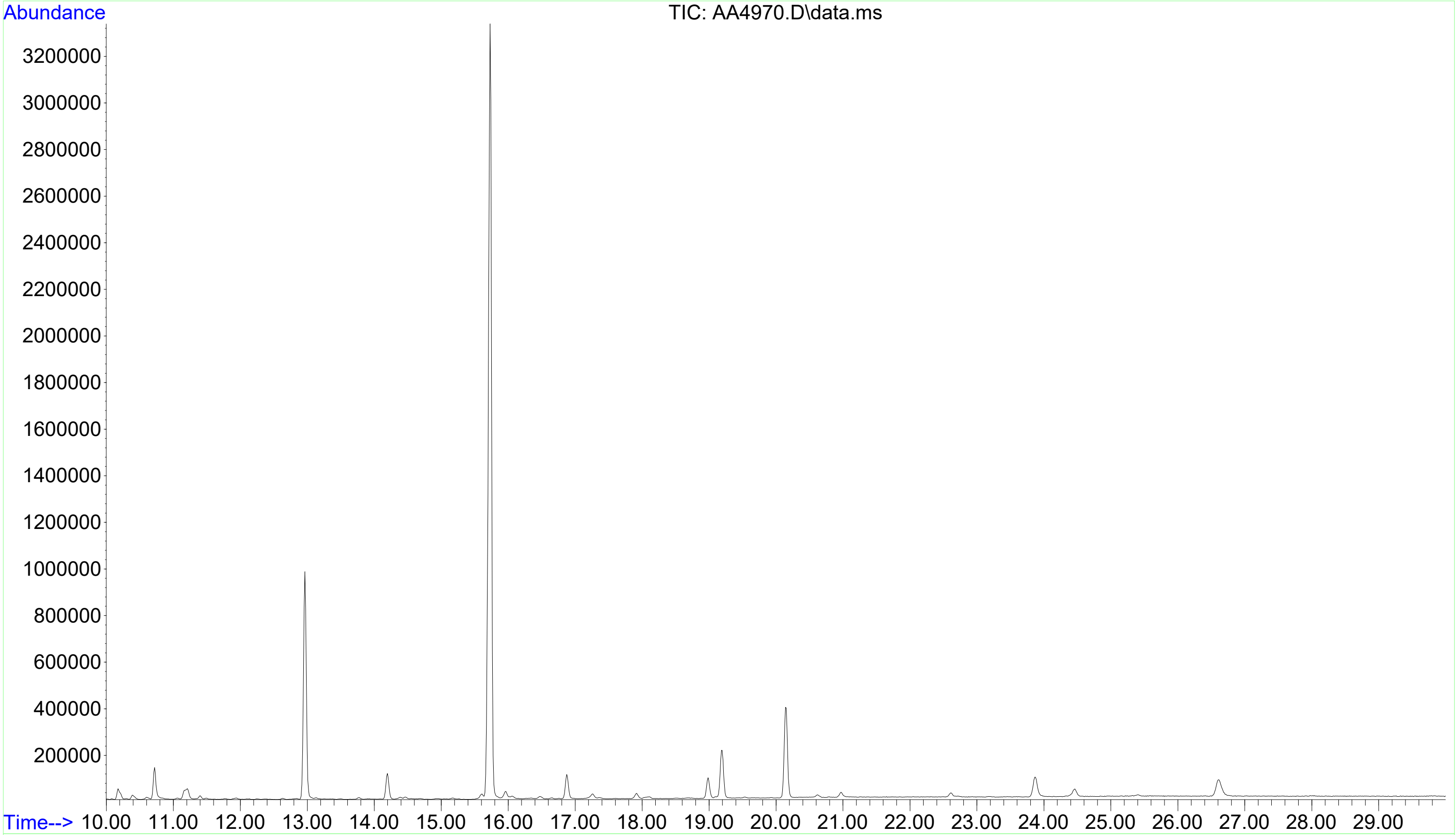

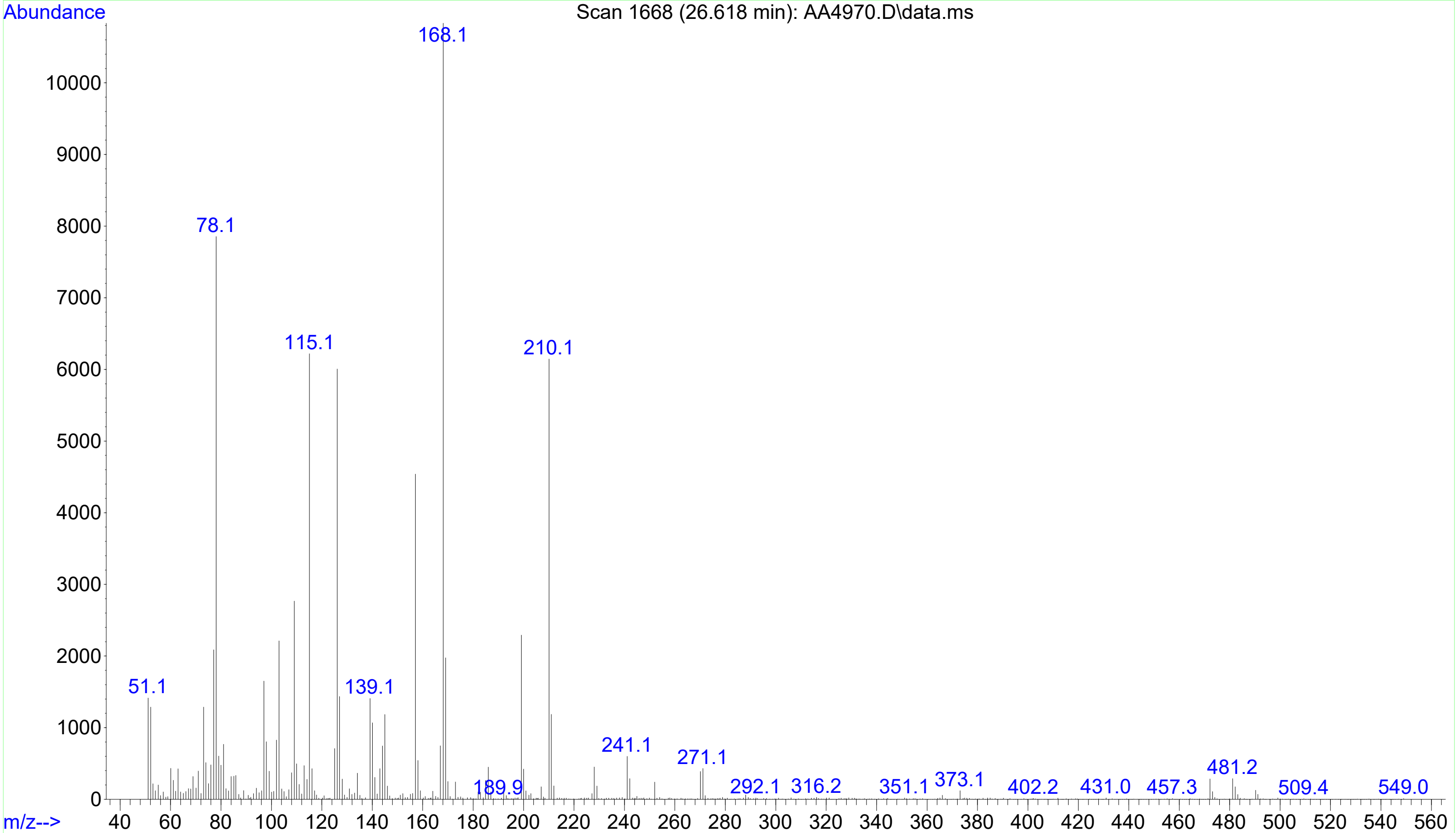

```
File        :E:\AA4971.D
Operator    : Artur
Acquired    : 17 Apr 2021  15:03     using AcqMethod SERVLABPMAA10TO1.M
Instrument  :   GC-MS AA
Sample Name: E. bicyclis Laminarin
Misc Info   :
Vial Number: 45
```

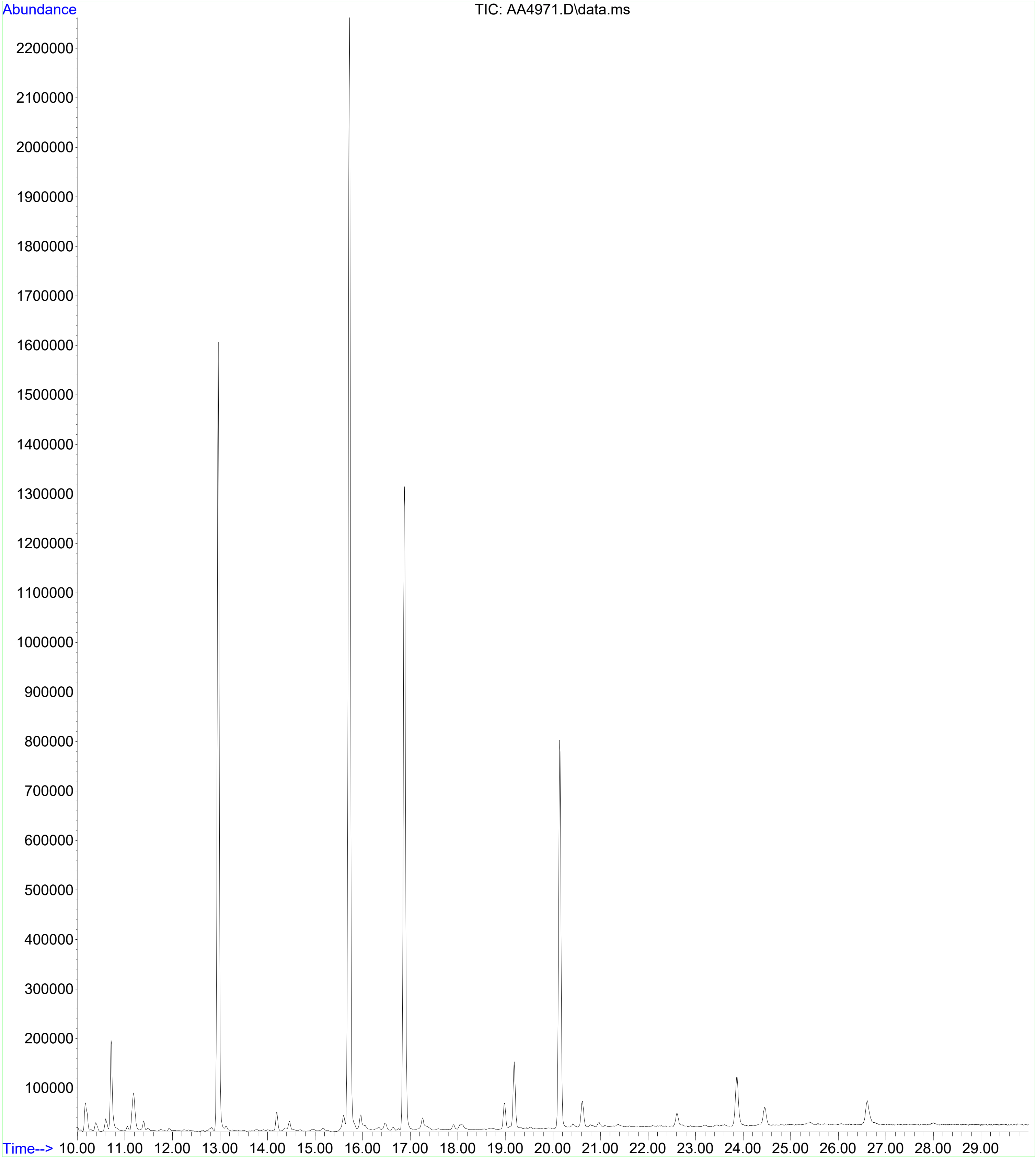

TIC: AA4971.D\data.ms
E. bicyclis Laminarin

Peak #	Ret Time	Type	Width	Area	Start Time	End Time
1	10.169	M	0.063	2193710	10.106	10.255
2	10.387	M	0.063	687014	10.345	10.459
3	10.598	M	0.059	920446	10.563	10.654
4	10.718	VB	0.046	5544134	10.66	10.891
5	11.187	VB	0.069	3279094	11.103	11.308
6	11.395	M	0.048	566217	11.328	11.442
7	12.968	BV	0.048	46525814	12.88	13.093
8	14.192	M	0.052	1232707	14.115	14.251
9	15.6	M	0.063	1109998	15.5	15.632
10	15.721	M	0.054	74356173	15.651	15.865
11	15.959	M	0.063	1169873	15.873	16.01
12	16.332	M	0.092	420607	16.25	16.399
13	16.476	M	0.069	660394	16.433	16.547
14	16.883	PB	0.05	42067570	16.703	17.061
15	17.26	M	0.068	976183	17.153	17.31
16	17.323	M	0.084	372238	17.323	17.48
17	17.91	M	0.075	521359	17.814	17.986
18	18.048	M	0.12	790626	18.008	18.19
19	18.983	M	0.059	1902710	18.888	19.037
20	19.19	VB	0.054	4515511	19.059	19.3
21	20.149	BB	0.056	27353198	20.03	20.308
22	20.618	M	0.063	1965375	20.544	20.681
23	20.789	M	0.059	154510	20.747	20.85
24	20.971	M	0.052	278126	20.917	21.007
25	22.608	M	0.069	1177497	22.531	22.657
26	23.873	BB	0.073	4662164	23.767	24.045
27	24.453	M	0.085	1960158	24.358	24.564
28	26.61	M	0.097	2861623	26.483	26.734

File   :E:\AA4971.D
Operator   : Artur
Acquired   : 17 Apr 2021   15:03   using AcqMethod SERVLABPMAA10TO1.M
Instrument :   GC-MS AA
Sample Name: E. bicyclis Laminarin
Misc Info   :
Vial Number: 45

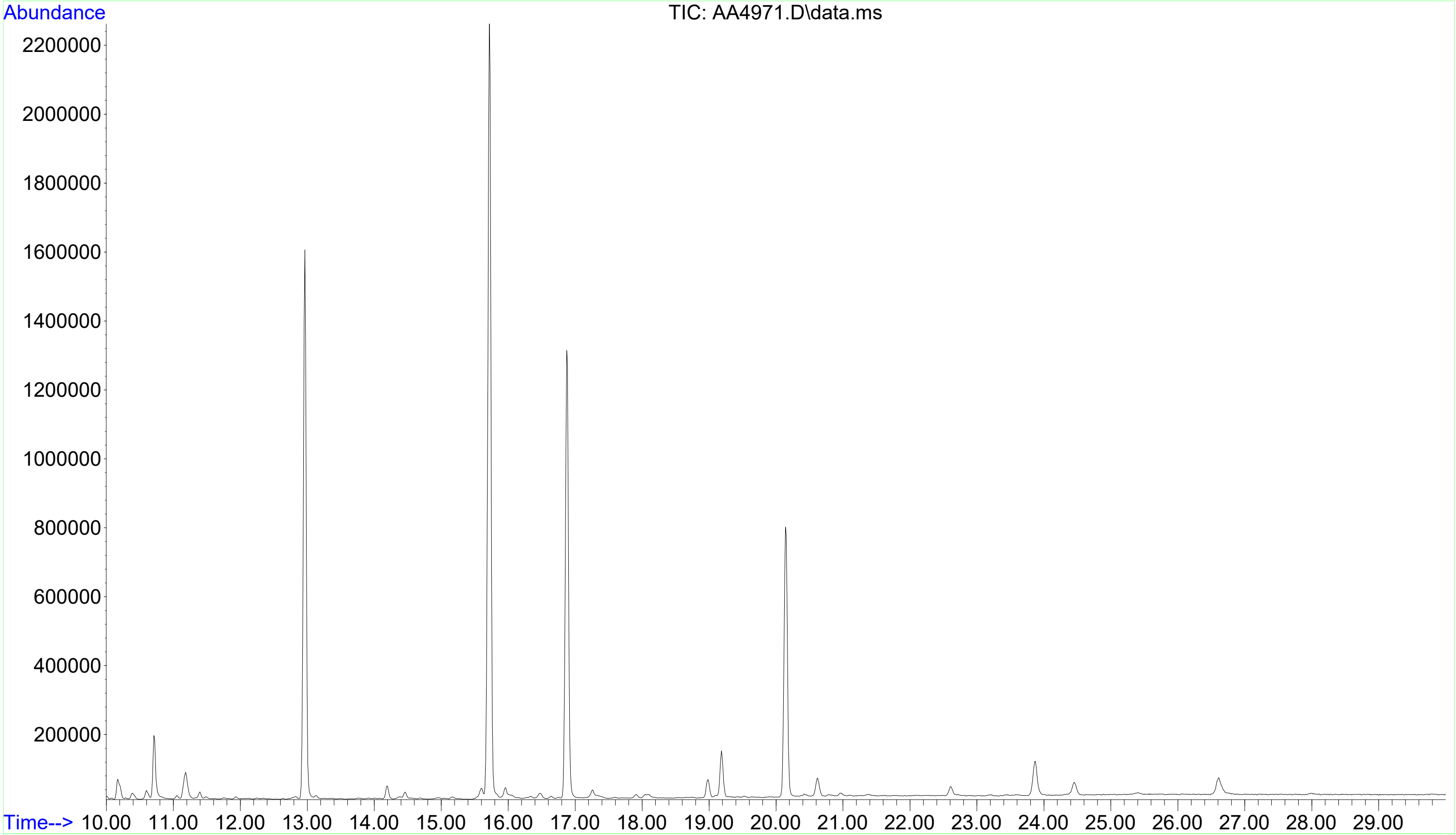

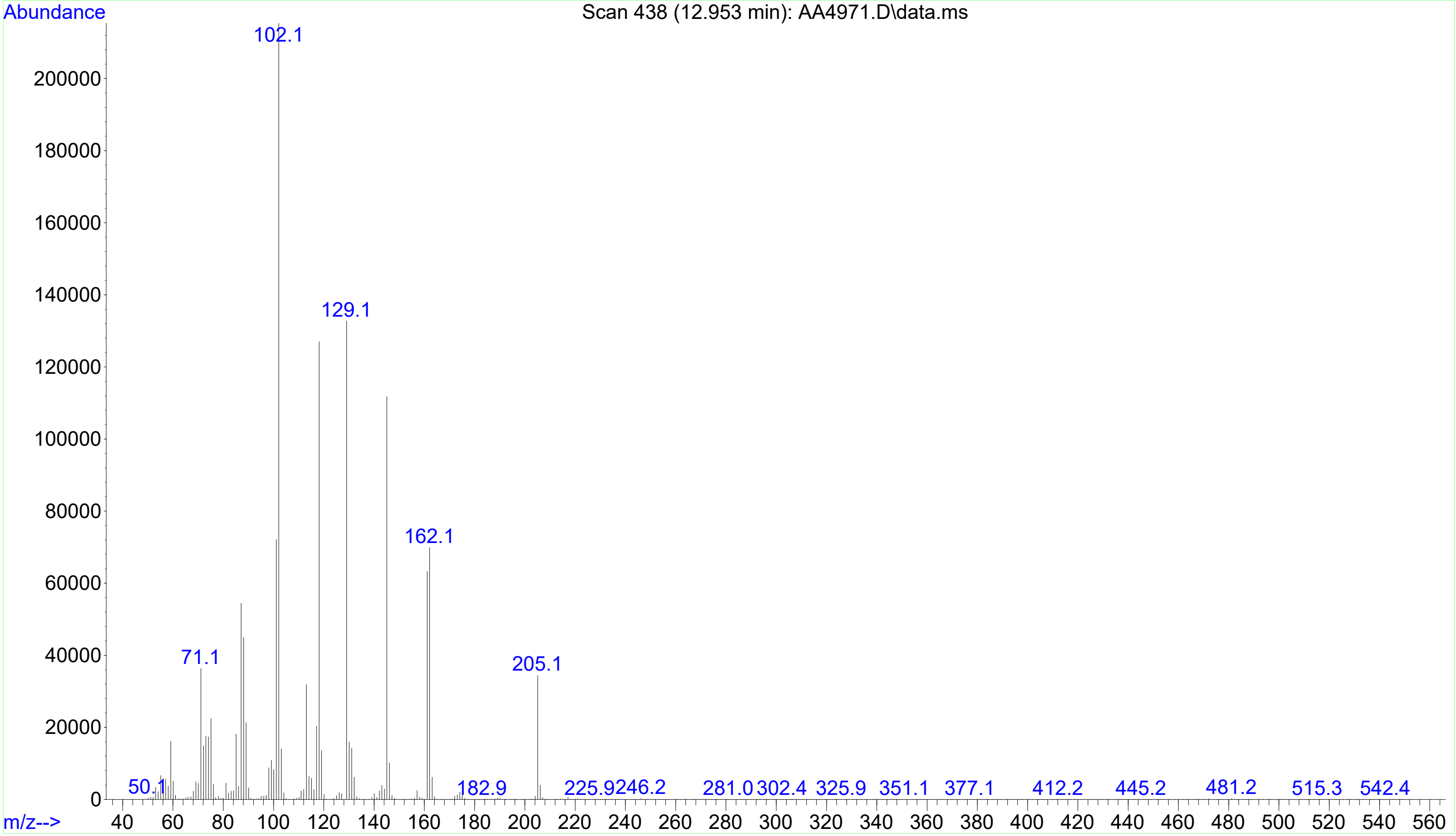

```
File        :E:\AA4971.D
Operator    : Artur
Acquired    : 17 Apr 2021  15:03     using AcqMethod SERVLABPMAA10TO1.M
Instrument :     GC-MS AA
Sample Name: E. bicyclis Laminarin
Misc Info   :
Vial Number: 45
```

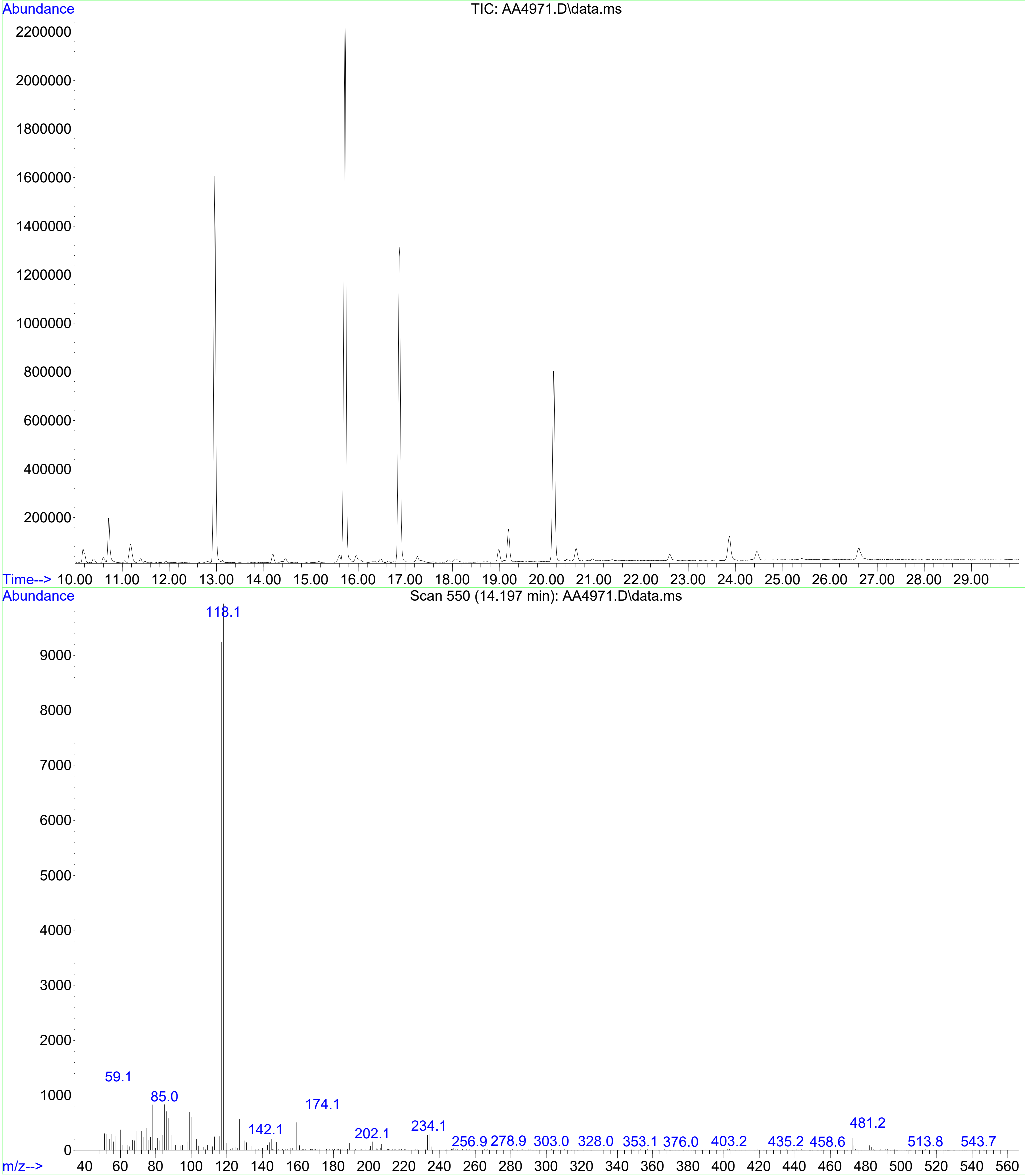

```
File        :E:\AA4971.D
Operator    : Artur
Acquired    : 17 Apr 2021   15:03      using AcqMethod SERVLABPMAA10TO1.M
Instrument  :     GC-MS AA
Sample Name: E. bicyclis Laminarin
Misc Info   :
Vial Number: 45
```

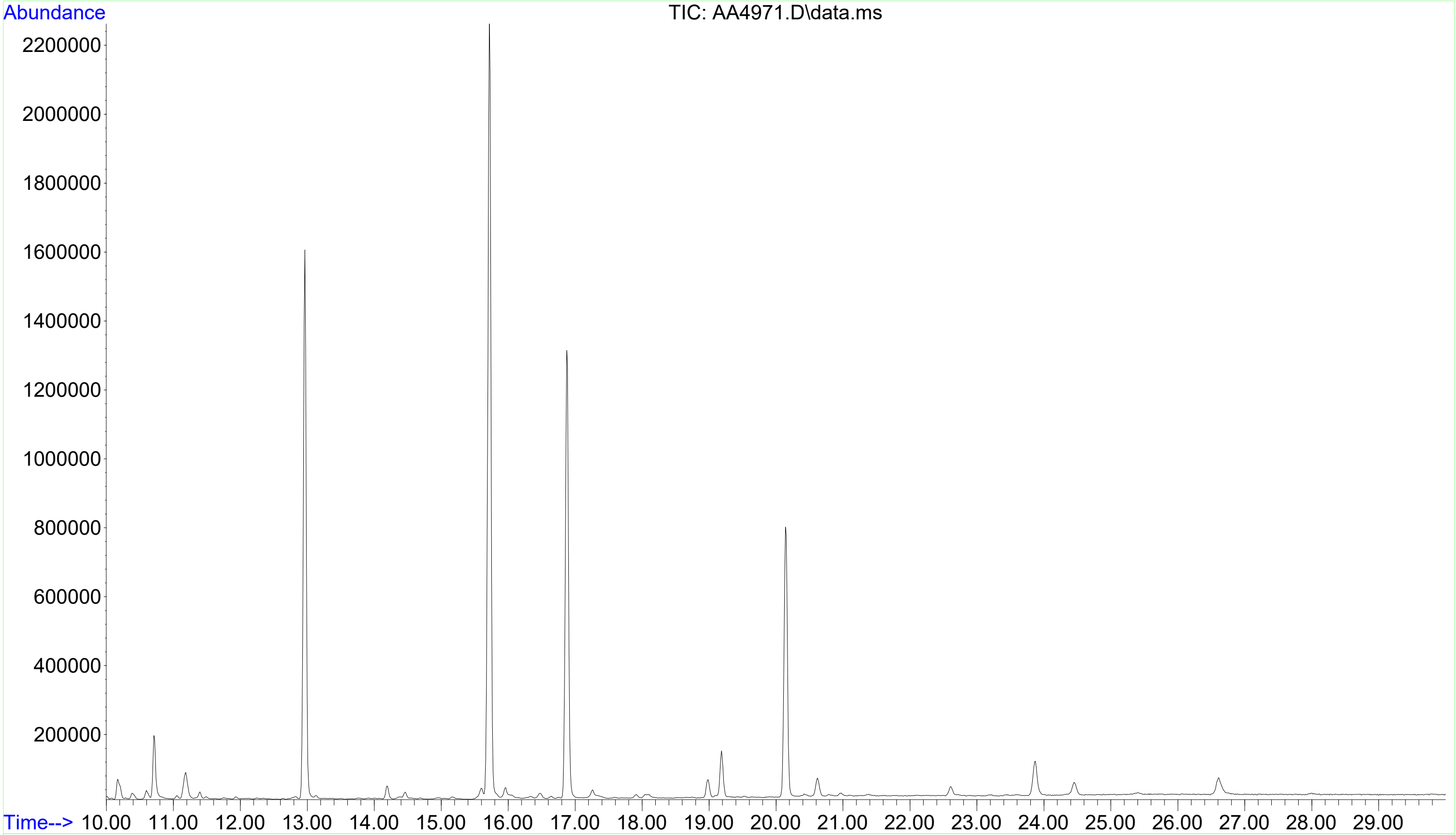

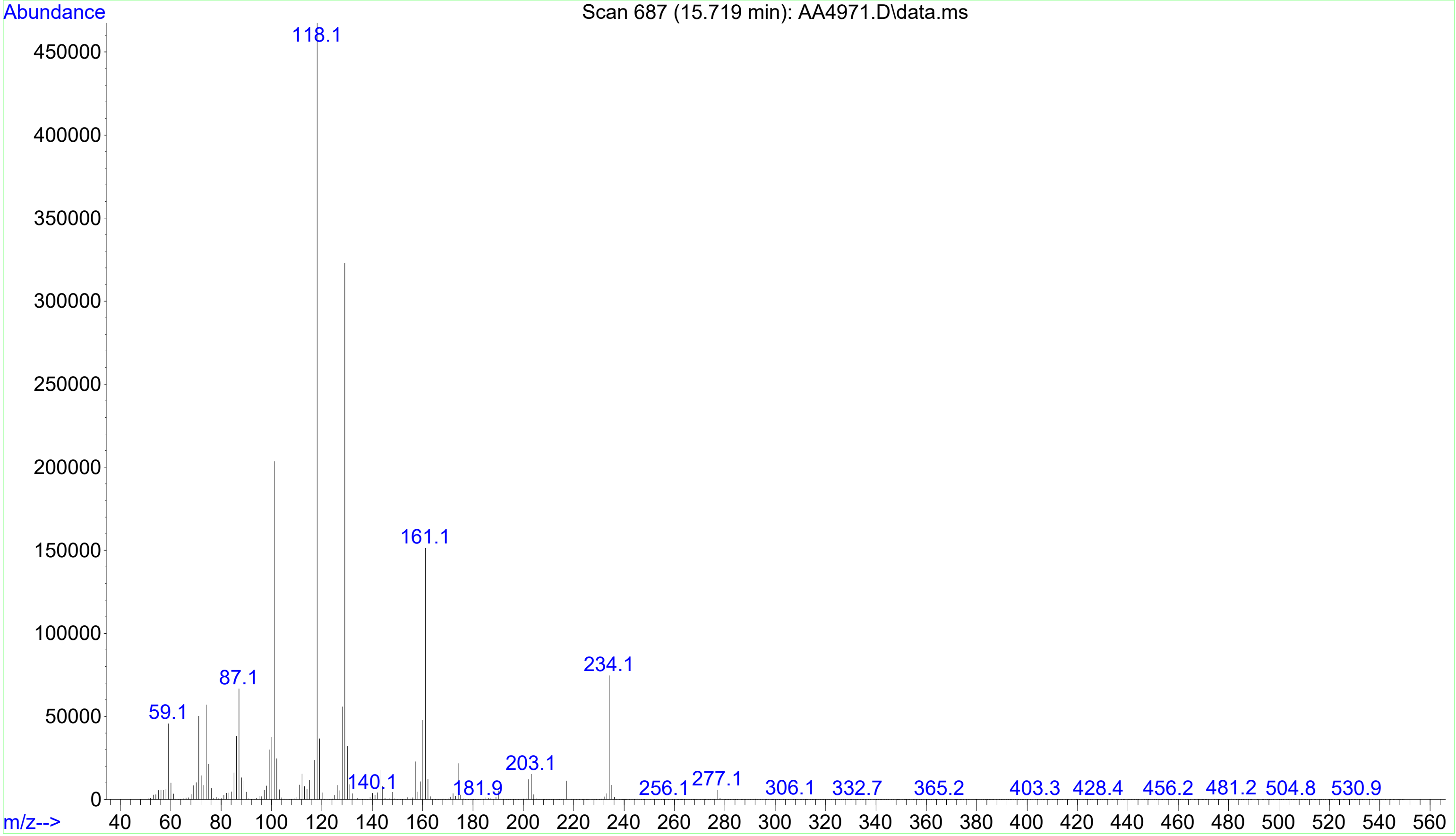

File :E:\AA4971.D
Operator : Artur
Acquired : 17 Apr 2021 15:03 using AcqMethod SERVLABPMAA10TO1.M
Instrument : GC-MS AA
Sample Name: E. bicyclis Laminarin
Misc Info :
Vial Number: 45

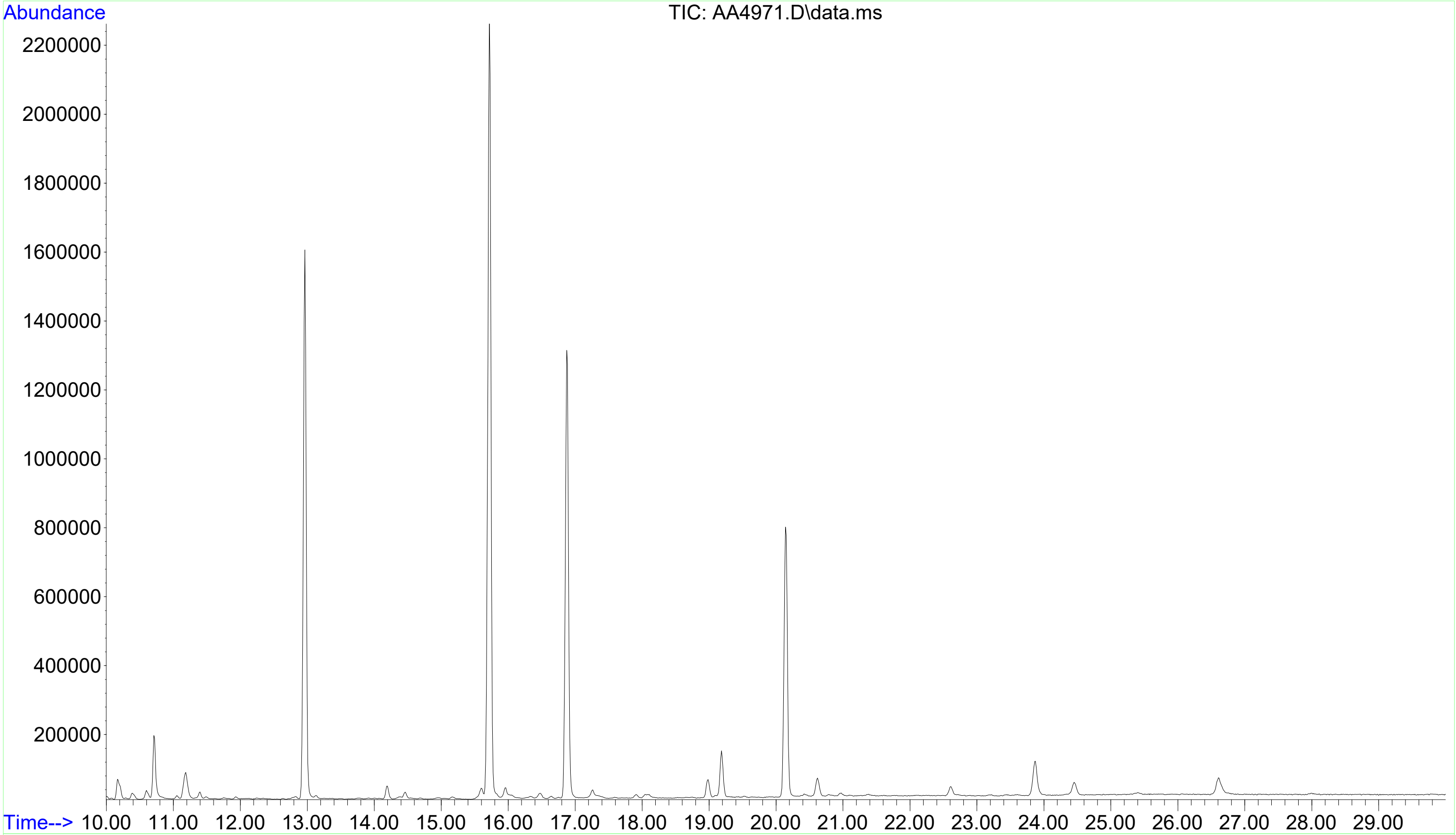

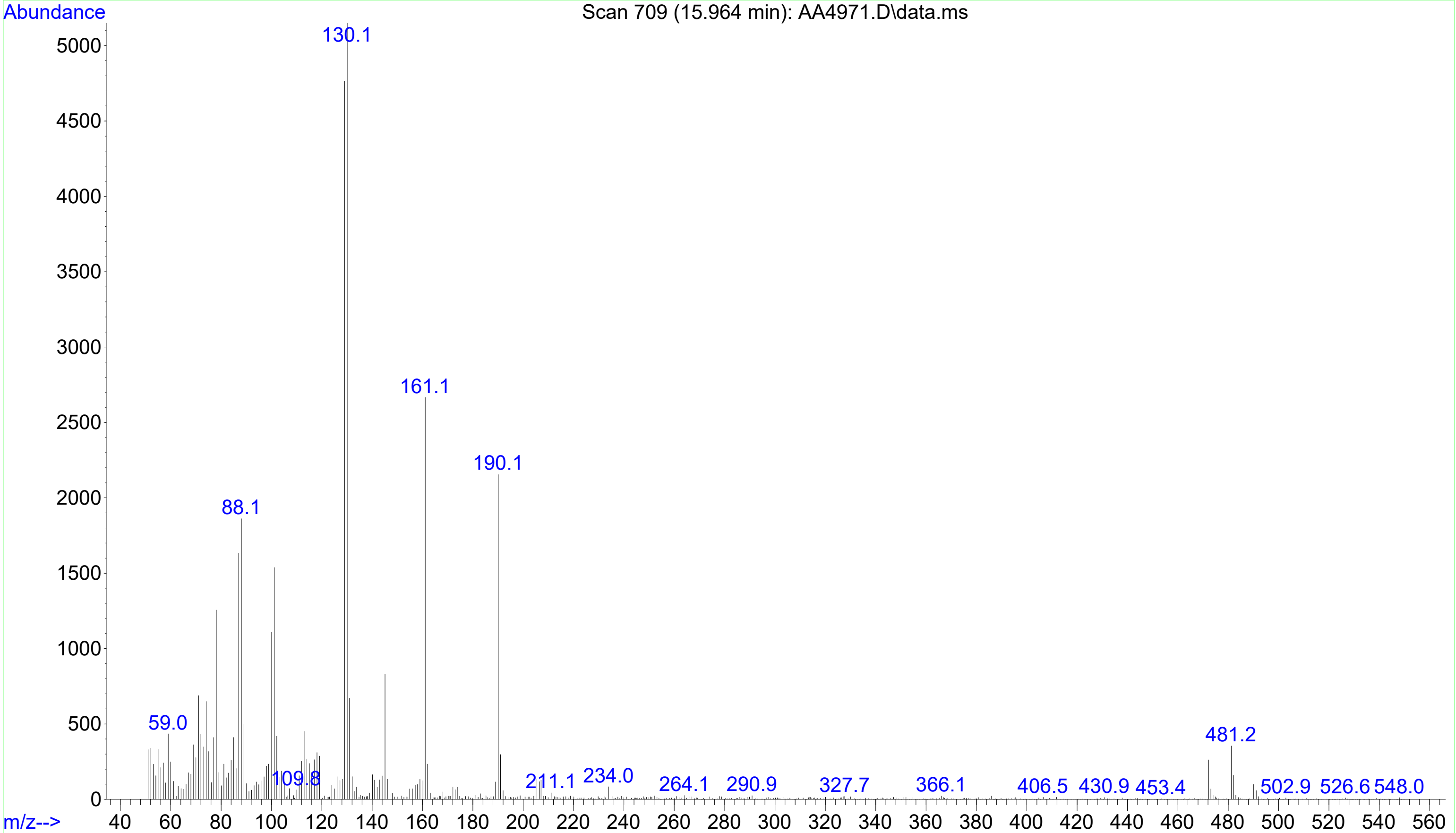

File   :E:\AA4971.D
Operator   : Artur
Acquired   : 17 Apr 2021   15:03   using AcqMethod SERVLABPMAA10TO1.M
Instrument :   GC-MS AA
Sample Name: E. bicyclis Laminarin
Misc Info   :
Vial Number: 45

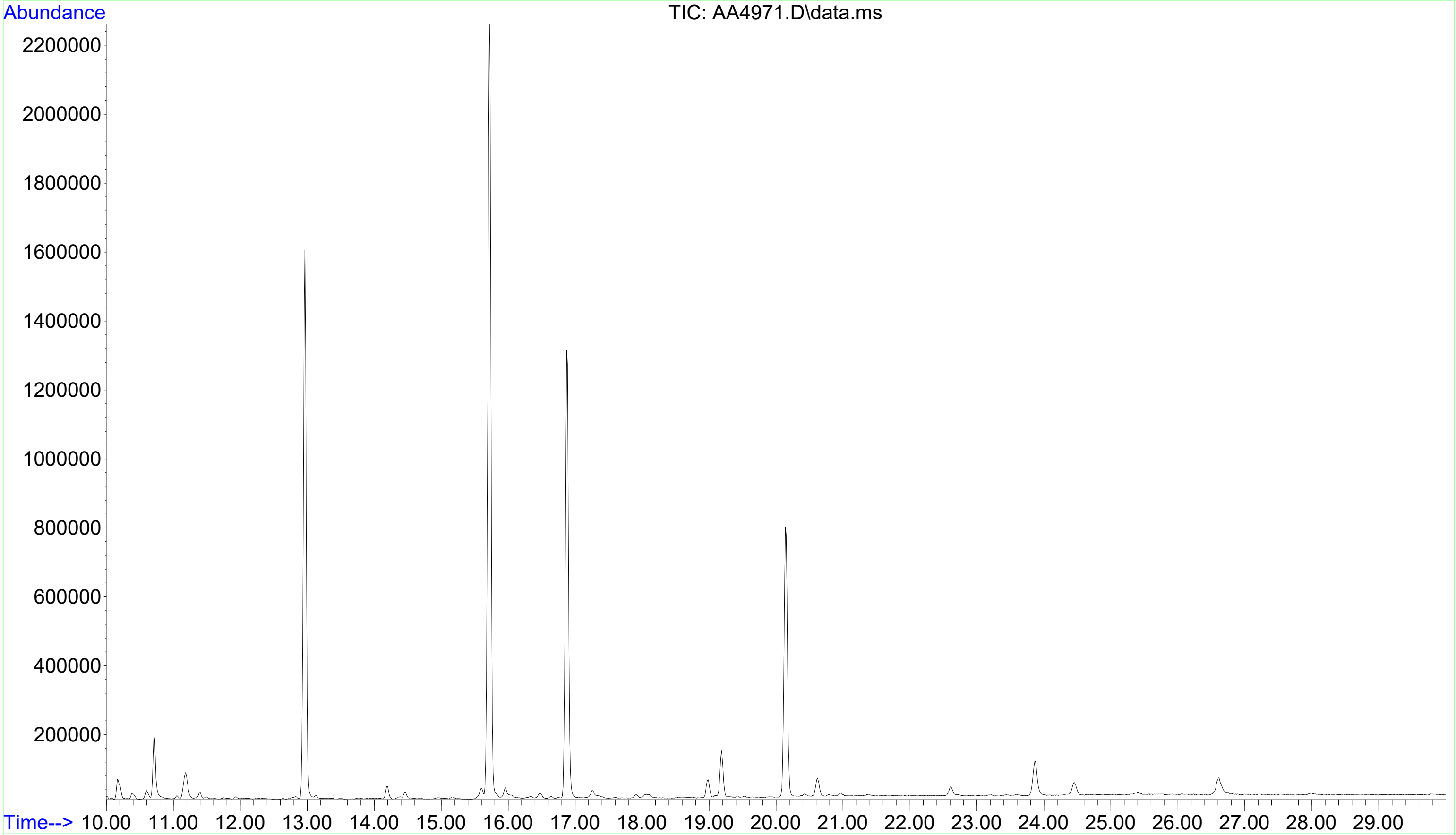

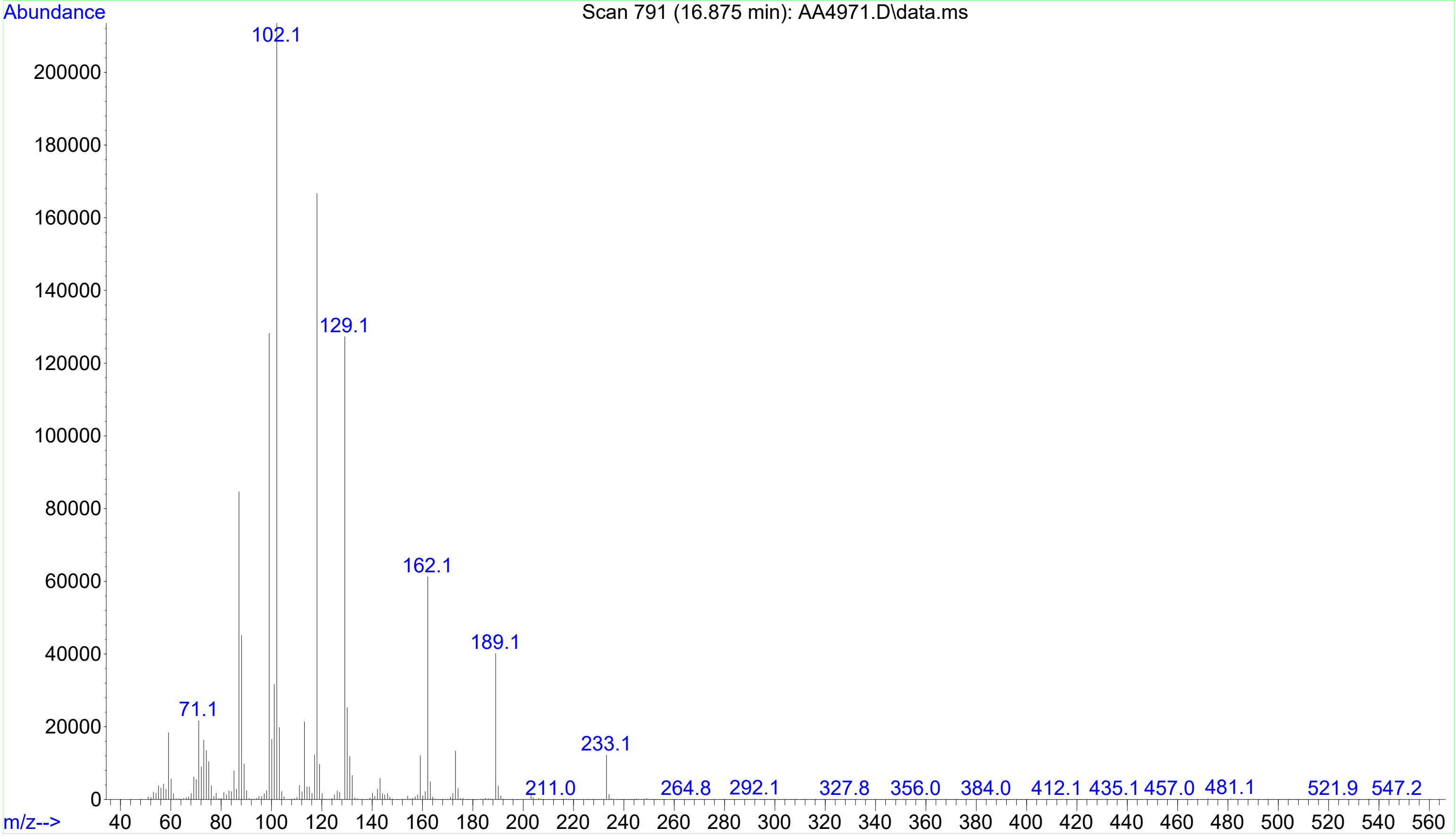

File :E:\AA4971.D
Operator : Artur
Acquired : 17 Apr 2021 15:03 using AcqMethod SERVLABPMAA10TO1.M
Instrument : GC-MS AA
Sample Name: E. bicyclis Laminarin
Misc Info :
Vial Number: 45

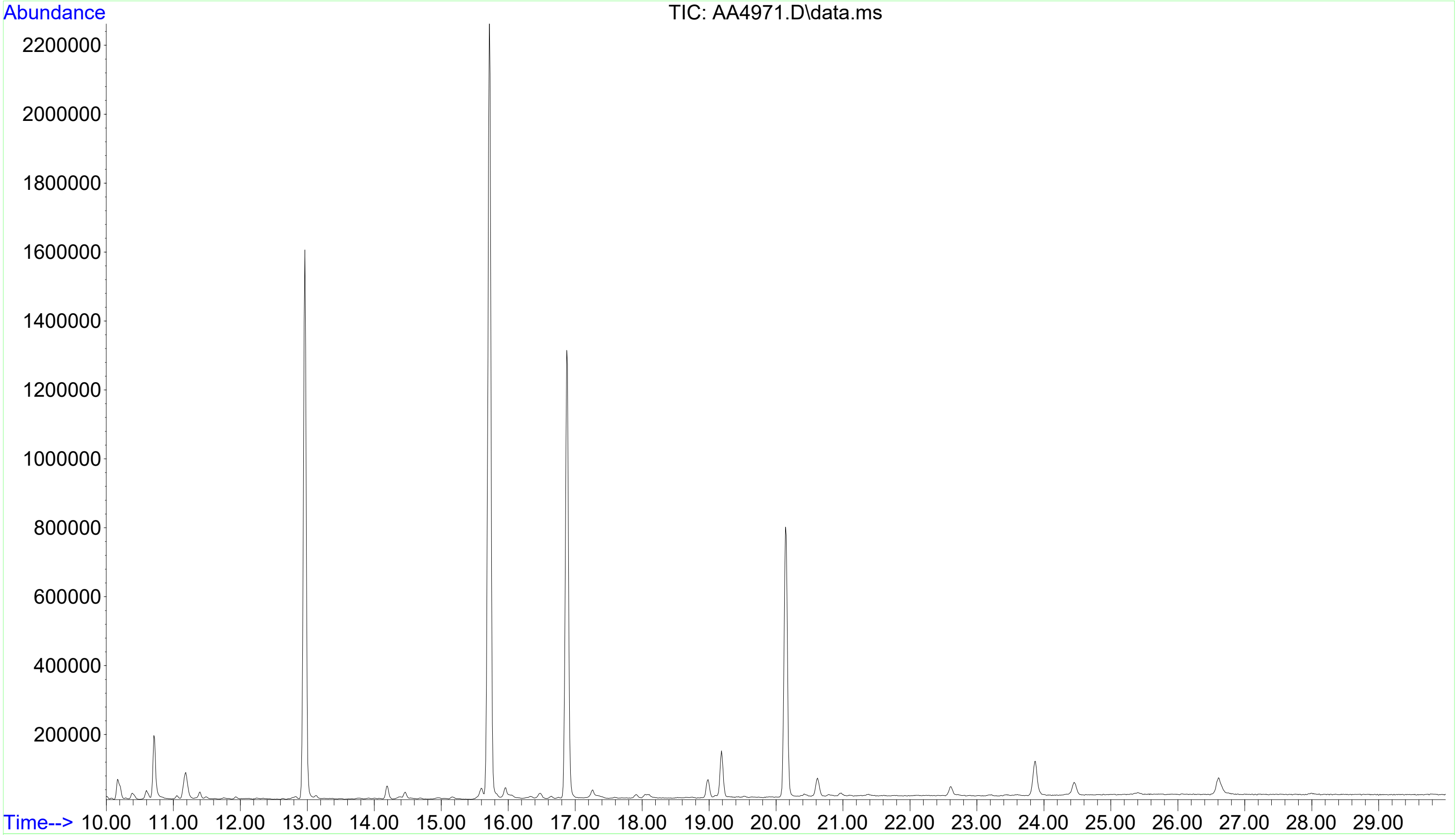

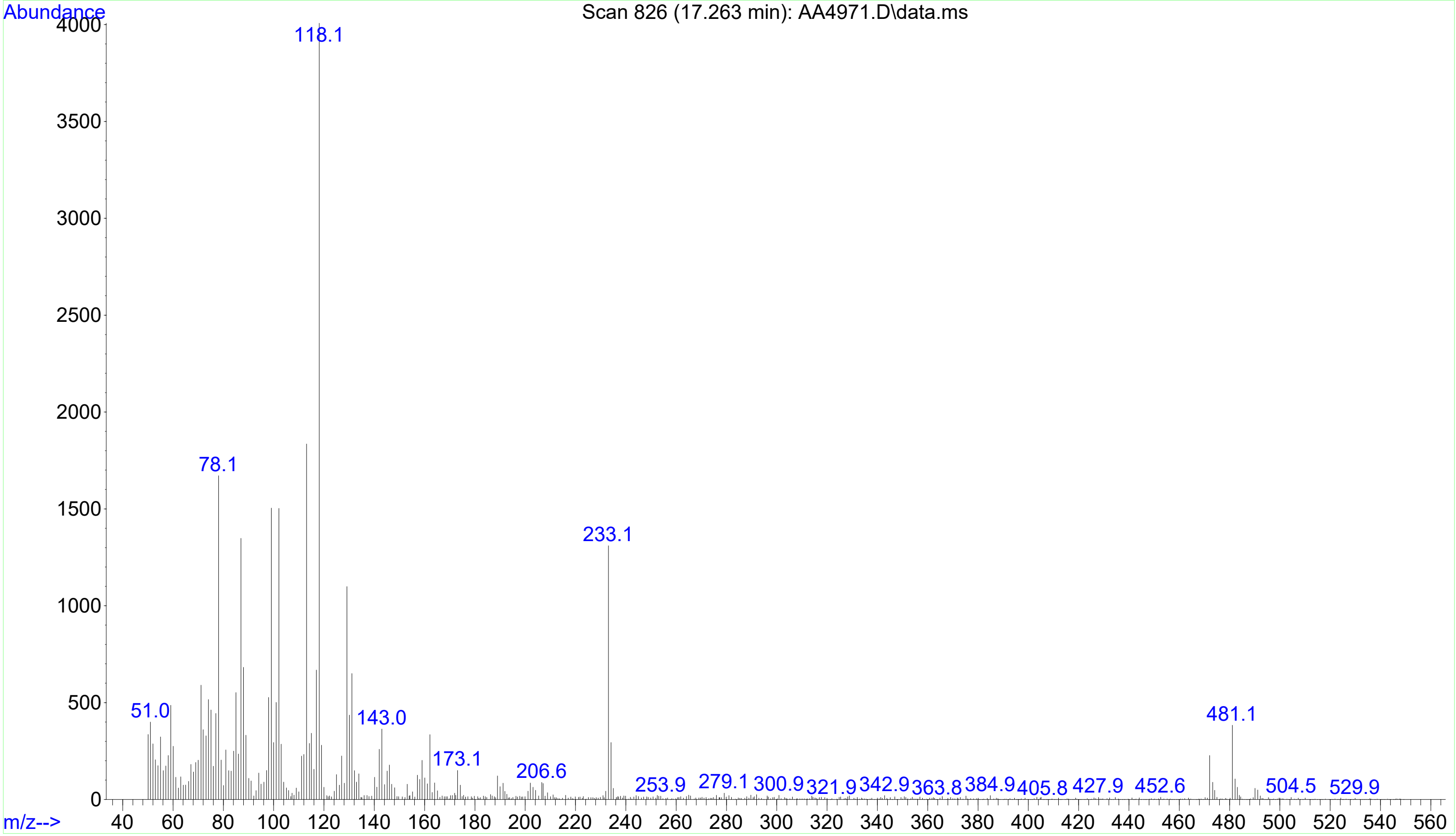

```
File        :E:\AA4971.D
Operator    : Artur
Acquired    : 17 Apr 2021   15:03      using AcqMethod SERVLABPMAA10TO1.M
Instrument :      GC-MS AA
Sample Name: E. bicyclis Laminarin
Misc Info   :
Vial Number: 45
```

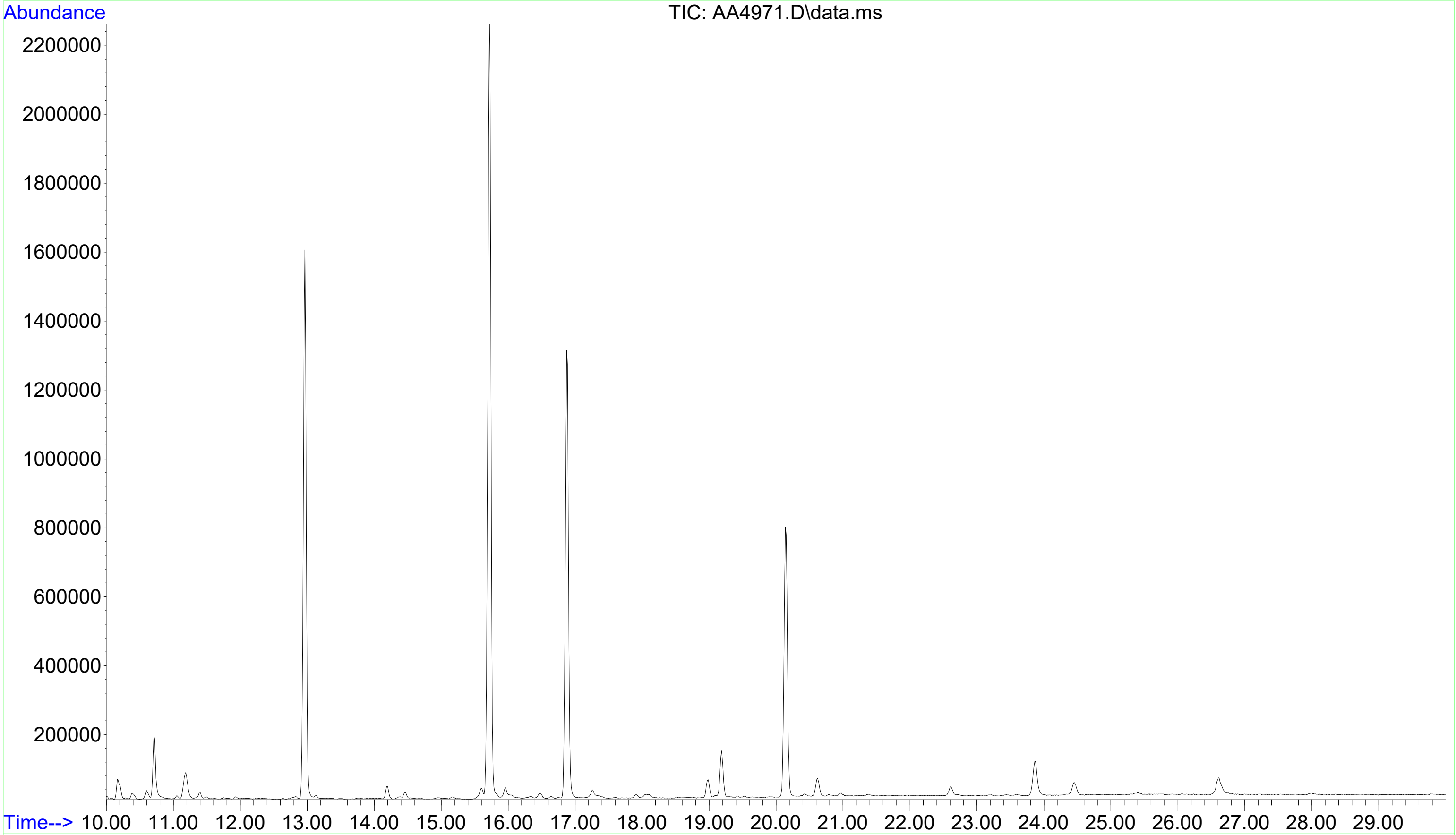

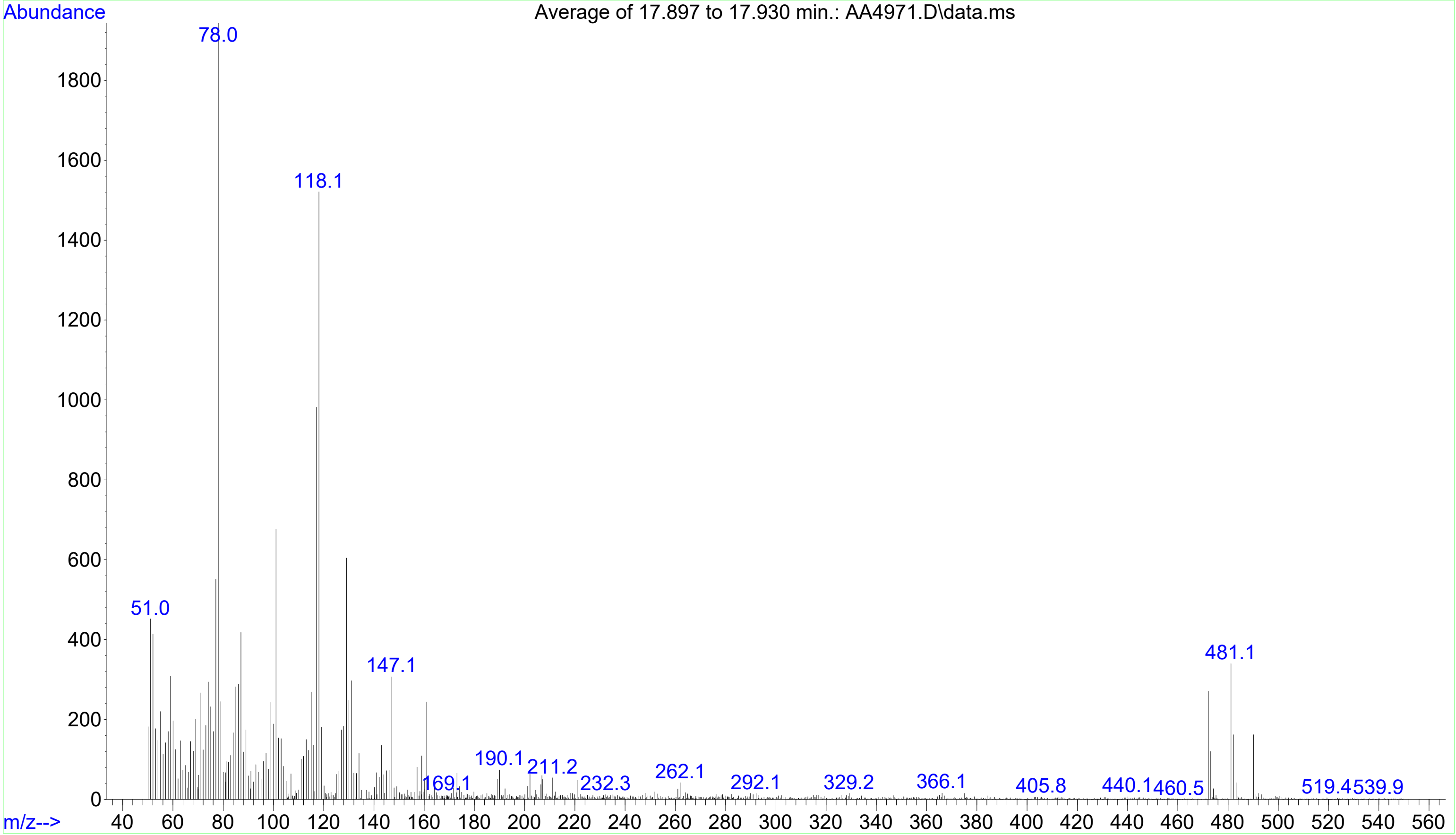

File   :E:\AA4971.D
Operator   : Artur
Acquired   : 17 Apr 2021   15:03   using AcqMethod SERVLABPMAA10TO1.M
Instrument :   GC-MS AA
Sample Name: E. bicyclis Laminarin
Misc Info   :
Vial Number: 45

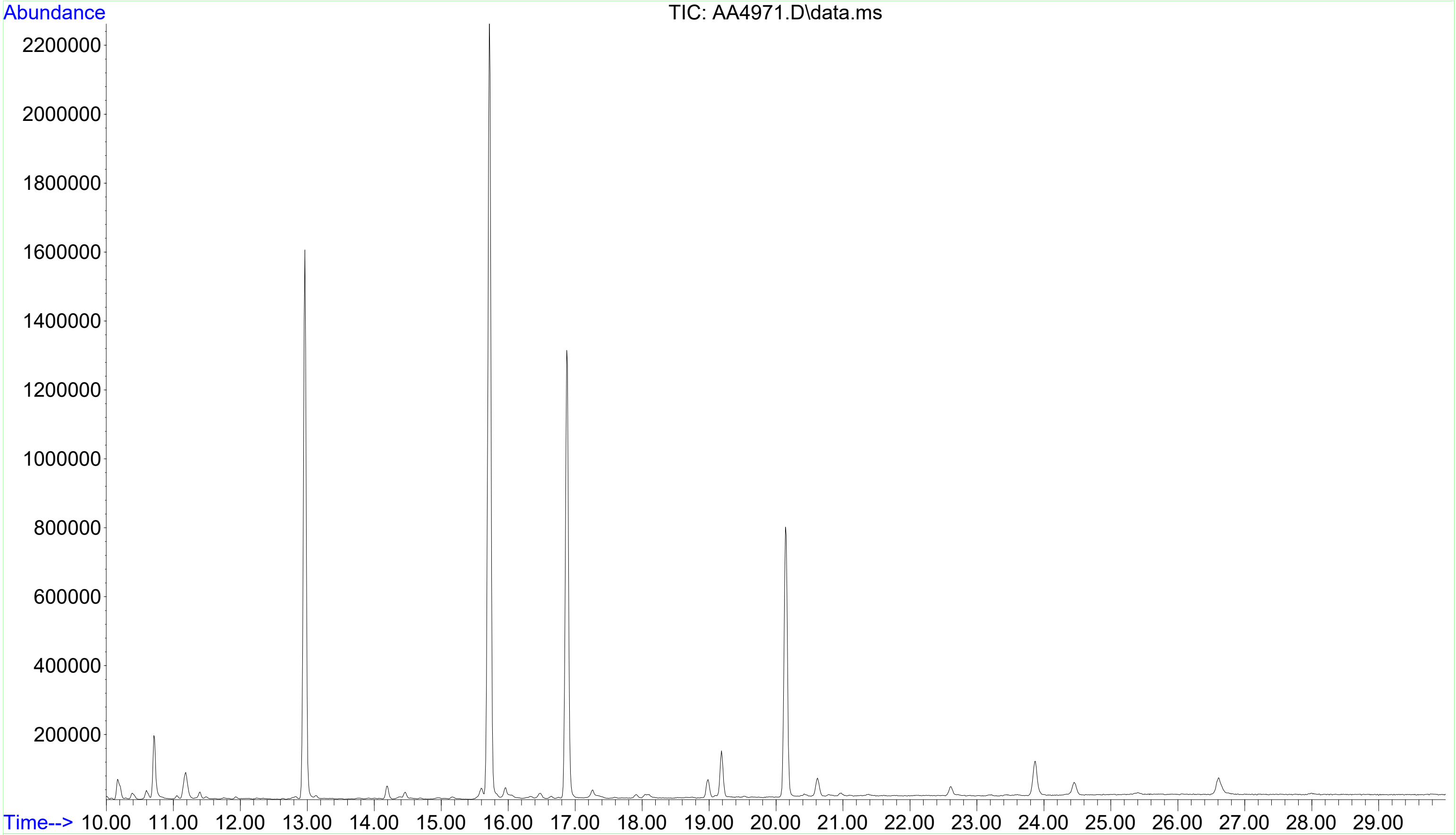

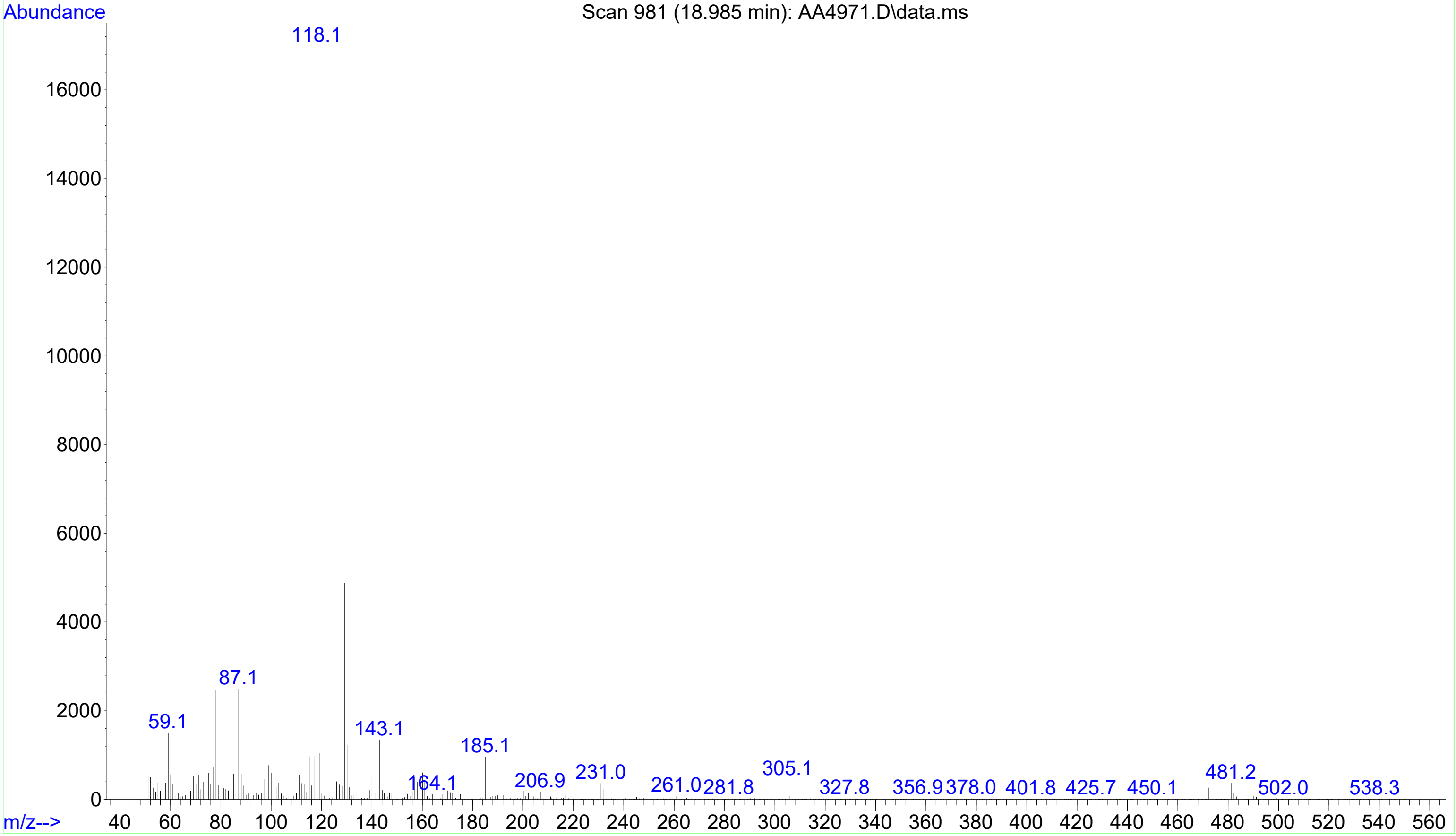

File :E:\AA4971.D
Operator : Artur
Acquired : 17 Apr 2021 15:03 using AcqMethod SERVLABPMAA10TO1.M
Instrument : GC-MS AA
Sample Name: E. bicyclis Laminarin
Misc Info :
Vial Number: 45

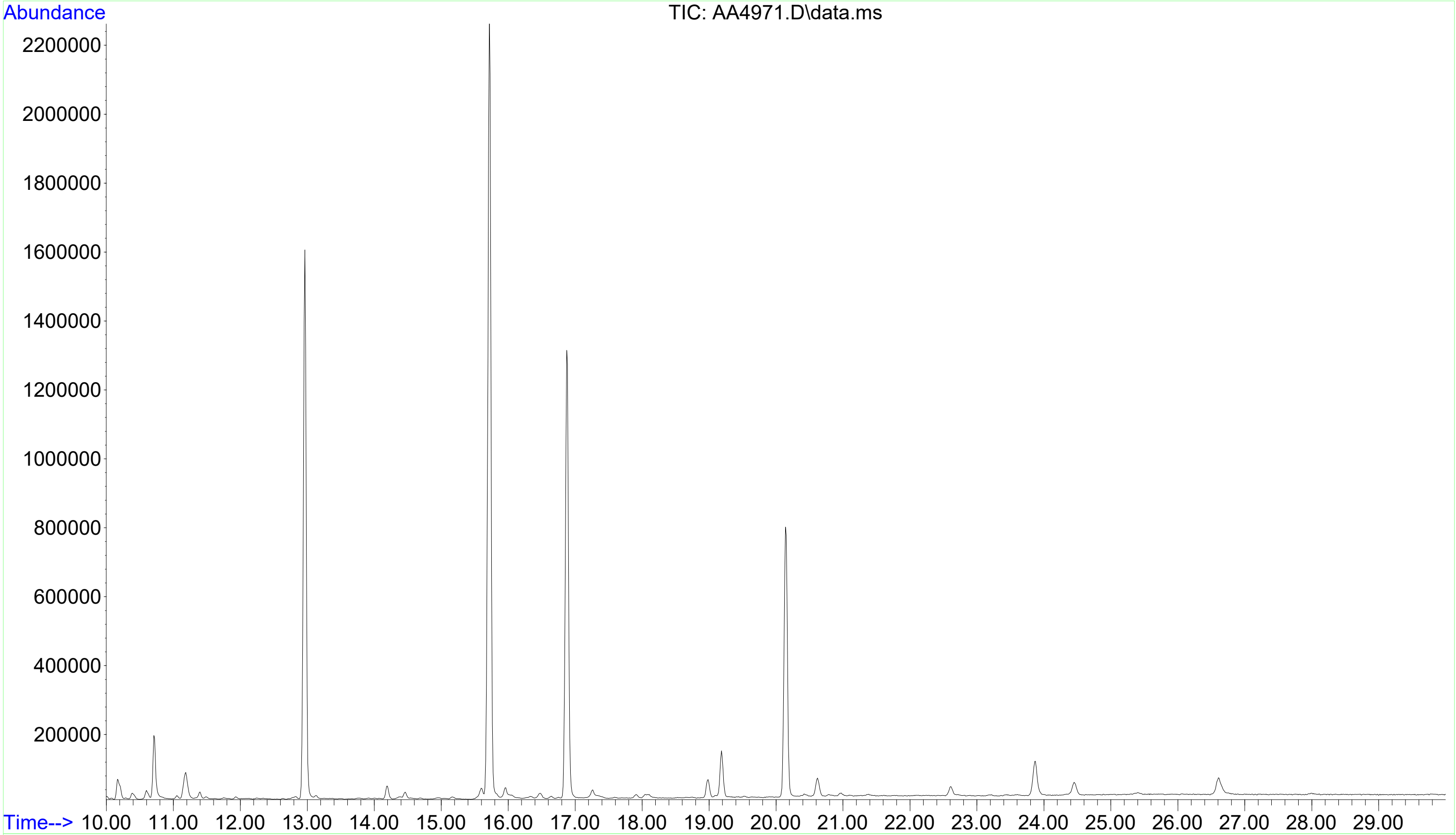

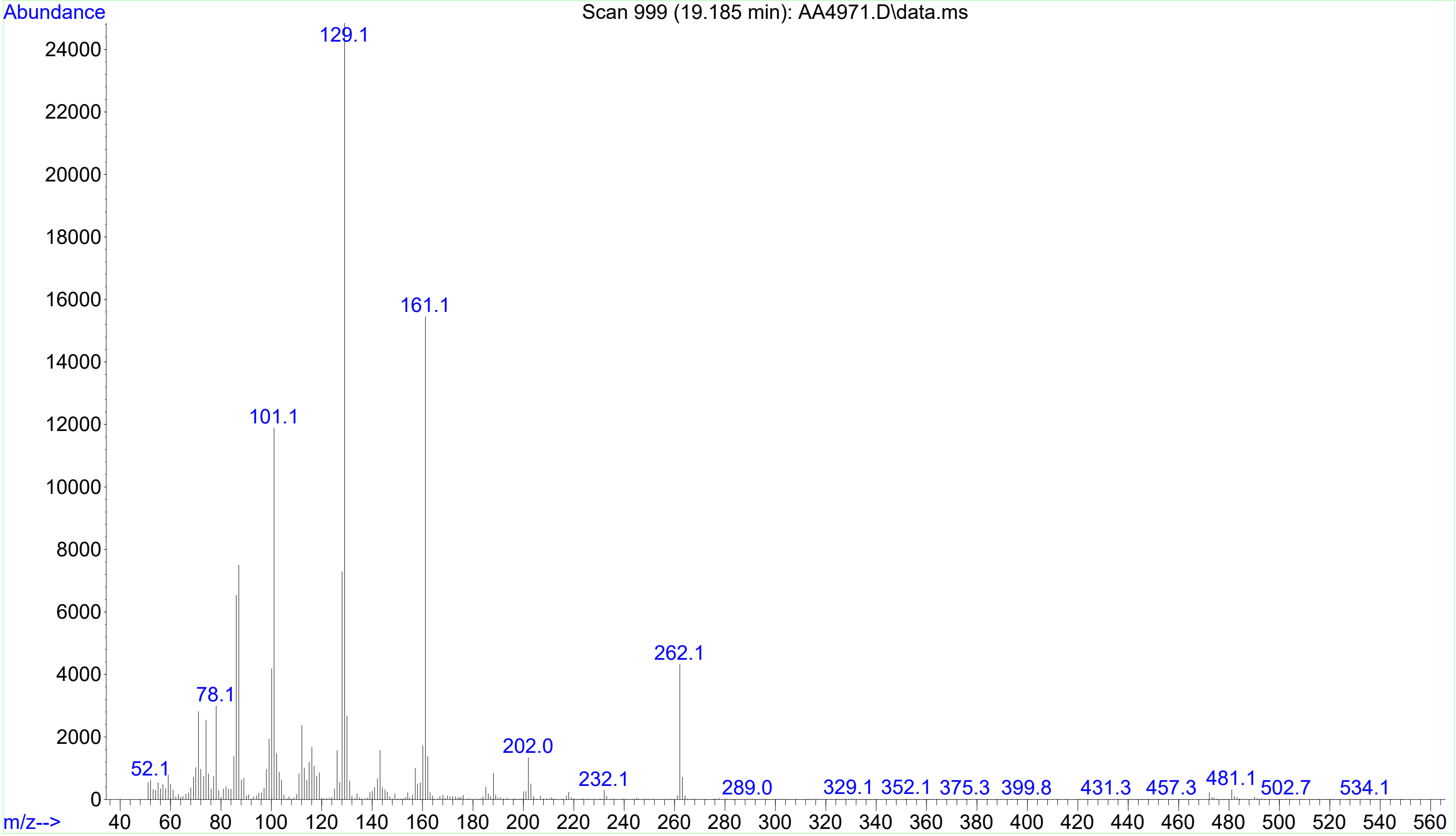

File :E:\AA4971.D
Operator : Artur
Acquired : 17 Apr 2021 15:03 using AcqMethod SERVLABPMAA10TO1.M
Instrument : GC-MS AA
Sample Name: E. bicyclis Laminarin
Misc Info :
Vial Number: 45

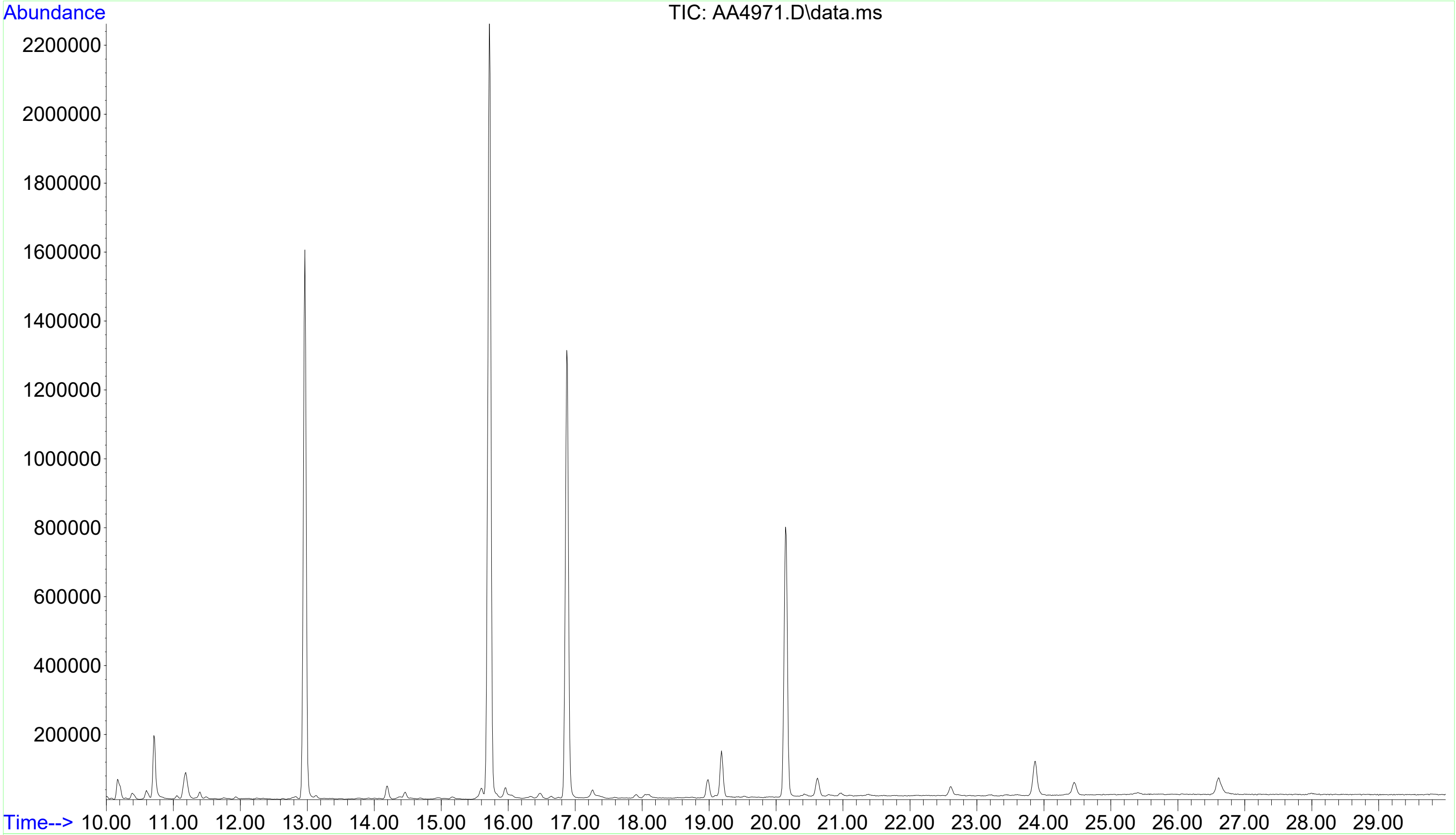

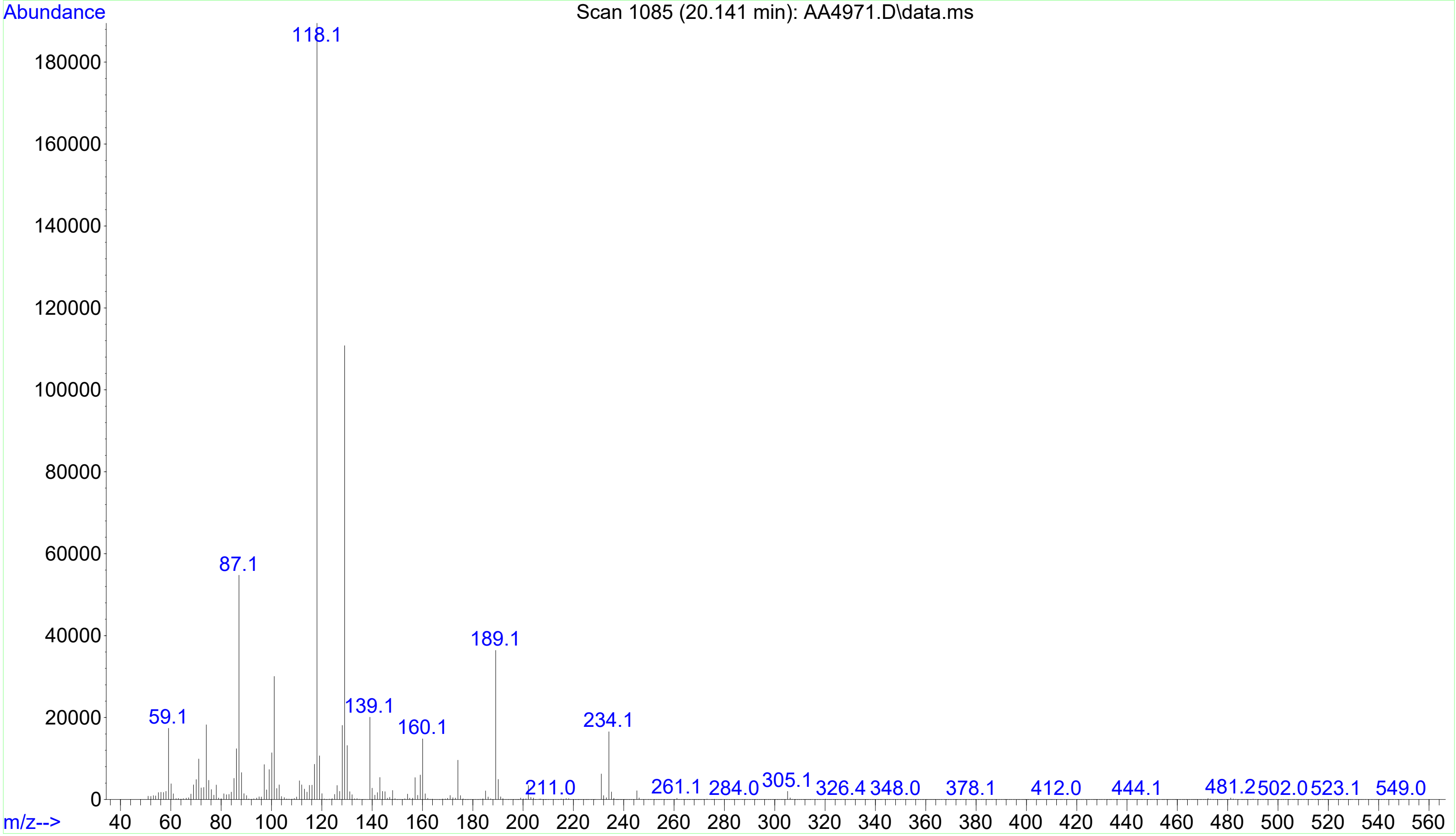

File :E:\AA4971.D
Operator : Artur
Acquired : 17 Apr 2021 15:03 using AcqMethod SERVLABPMAA10TO1.M
Instrument : GC-MS AA
Sample Name: E. bicyclis Laminarin
Misc Info :
Vial Number: 45

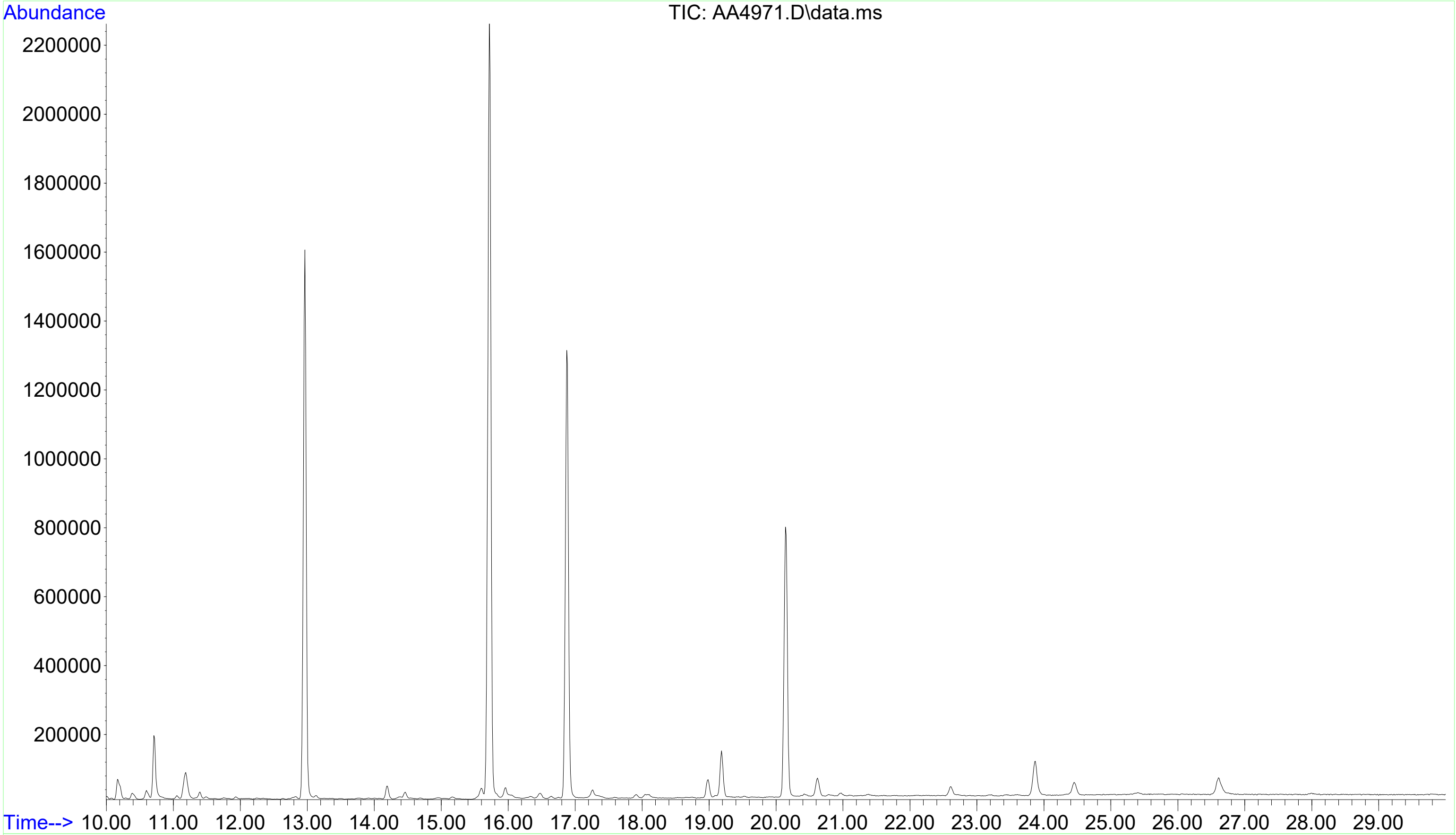

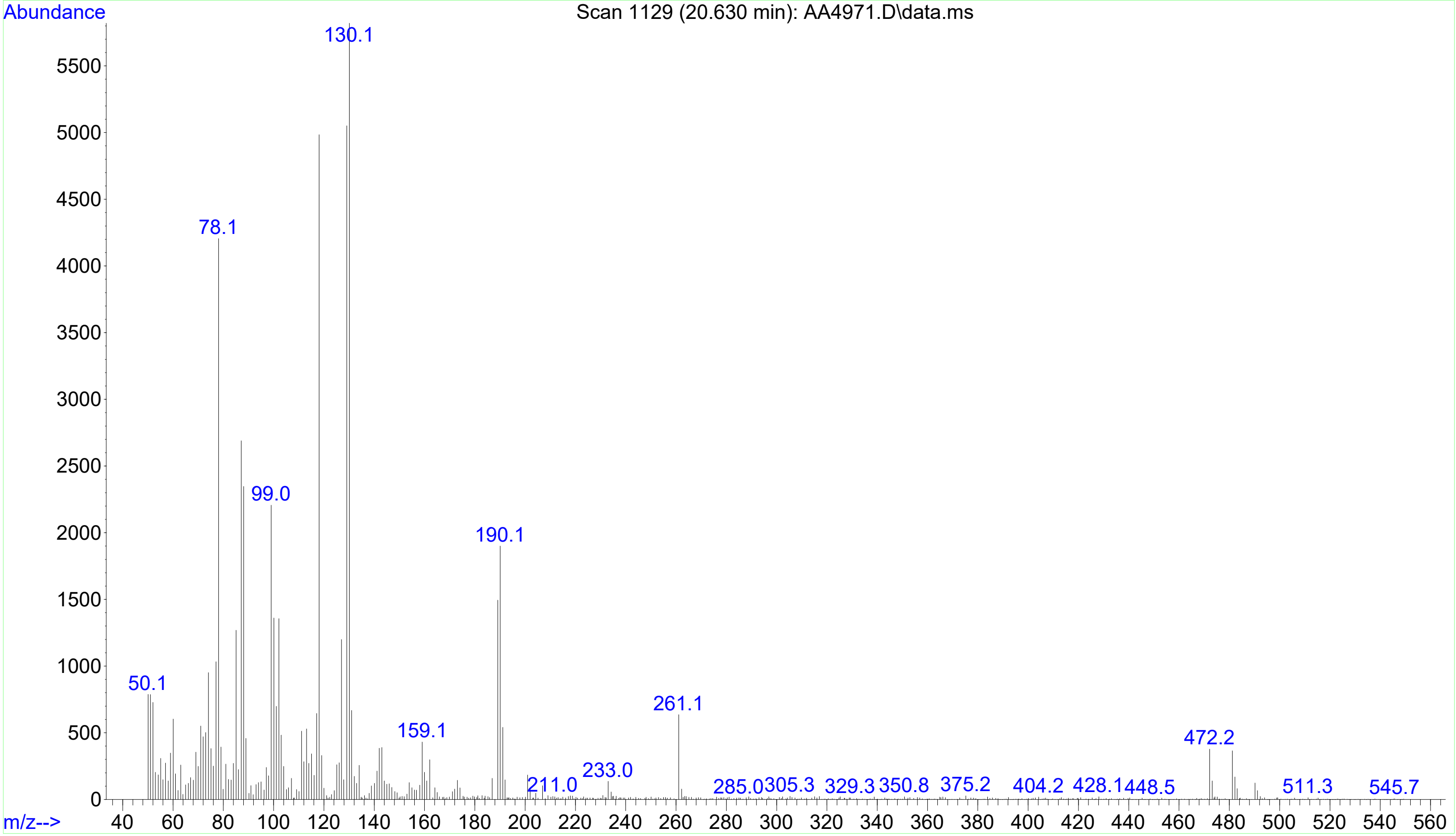

File   :E:\AA4971.D
Operator   : Artur
Acquired   : 17 Apr 2021   15:03   using AcqMethod SERVLABPMAA10TO1.M
Instrument :   GC-MS AA
Sample Name: E. bicyclis Laminarin
Misc Info   :
Vial Number: 45

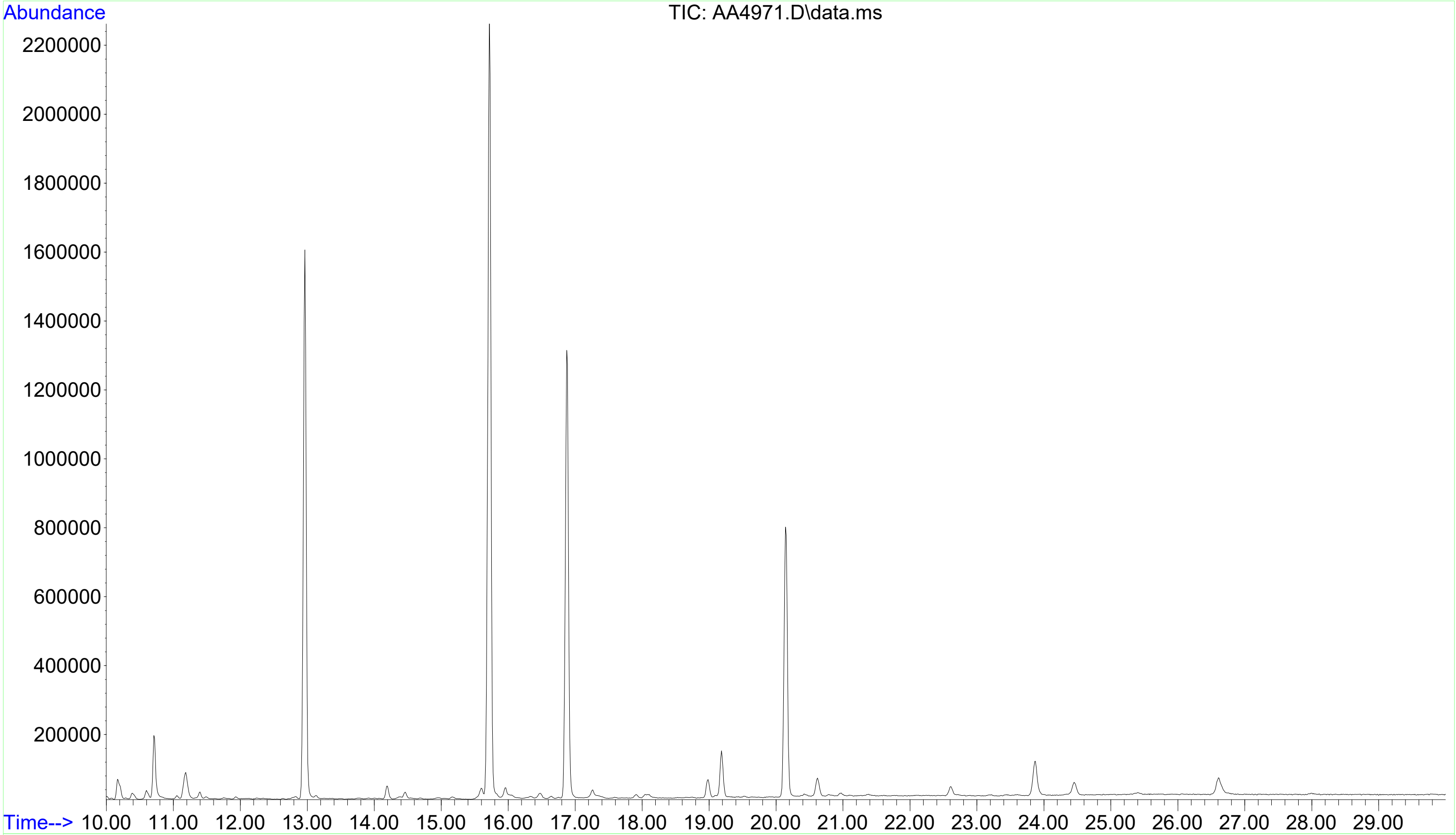

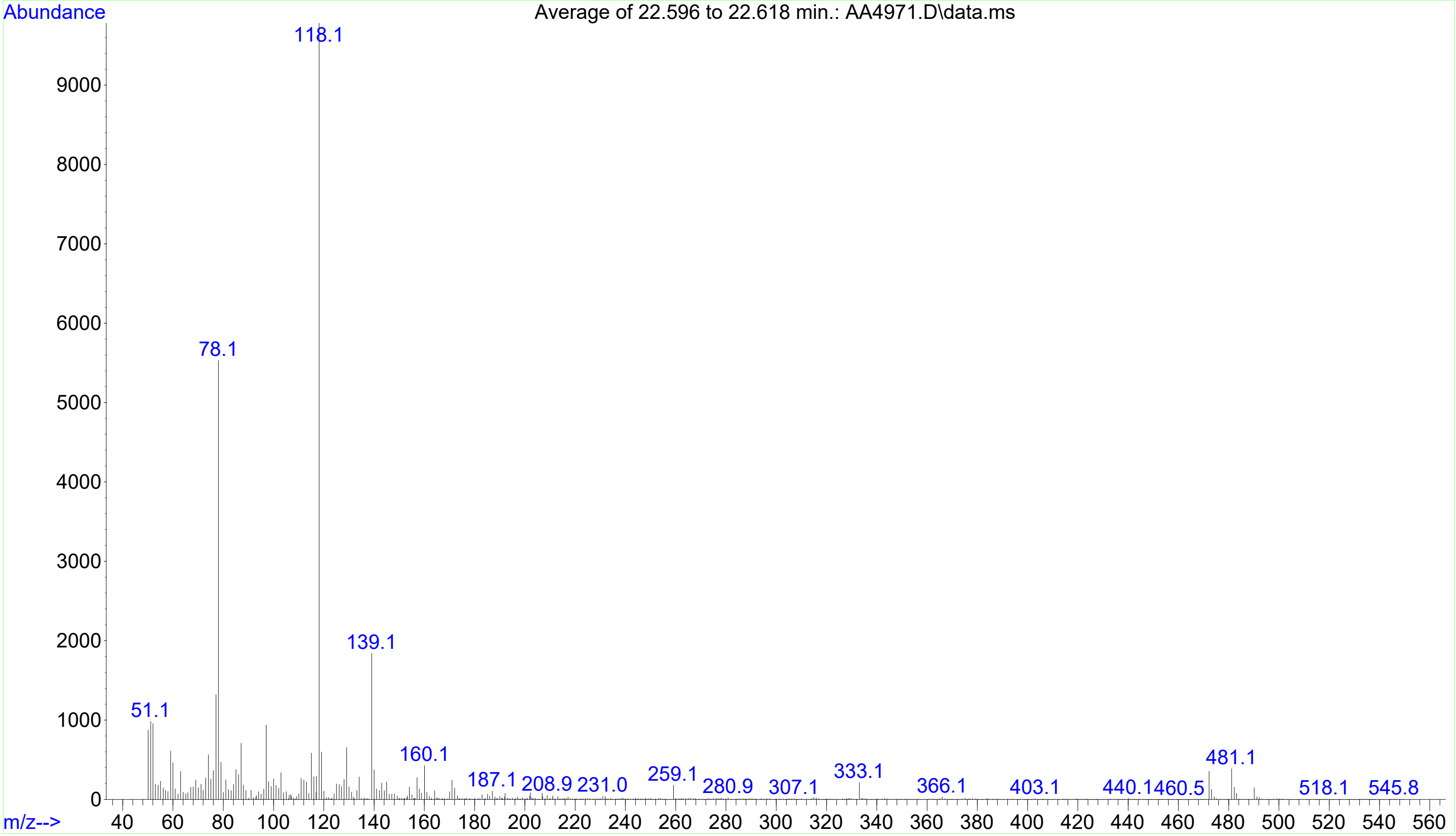

```
File        :E:\AA4971.D
Operator    : Artur
Acquired    : 17 Apr 2021   15:03     using AcqMethod SERVLABPMAA10TO1.M
Instrument  :     GC-MS AA
Sample Name: E. bicyclis Laminarin
Misc Info   :
Vial Number: 45
```

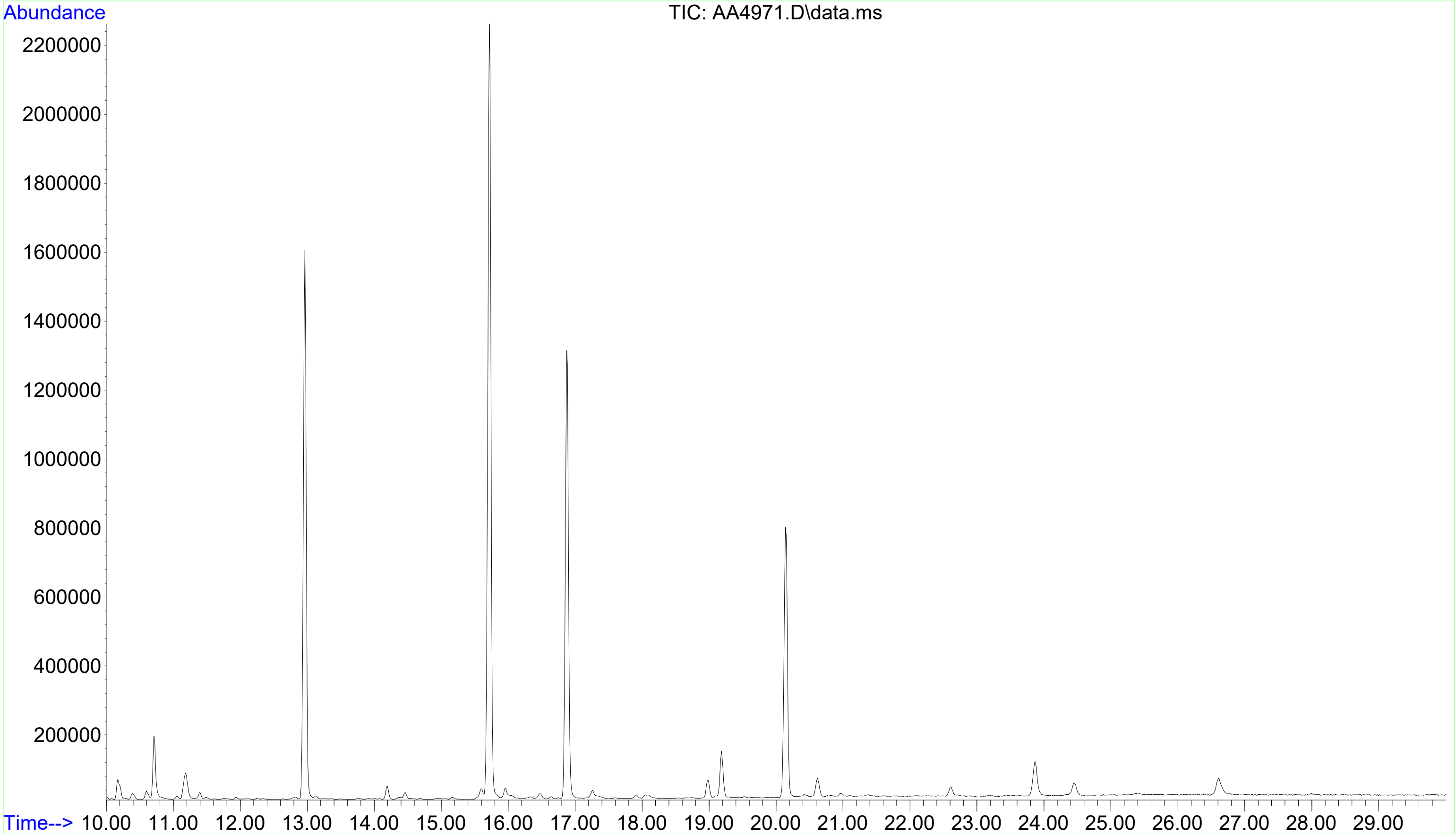

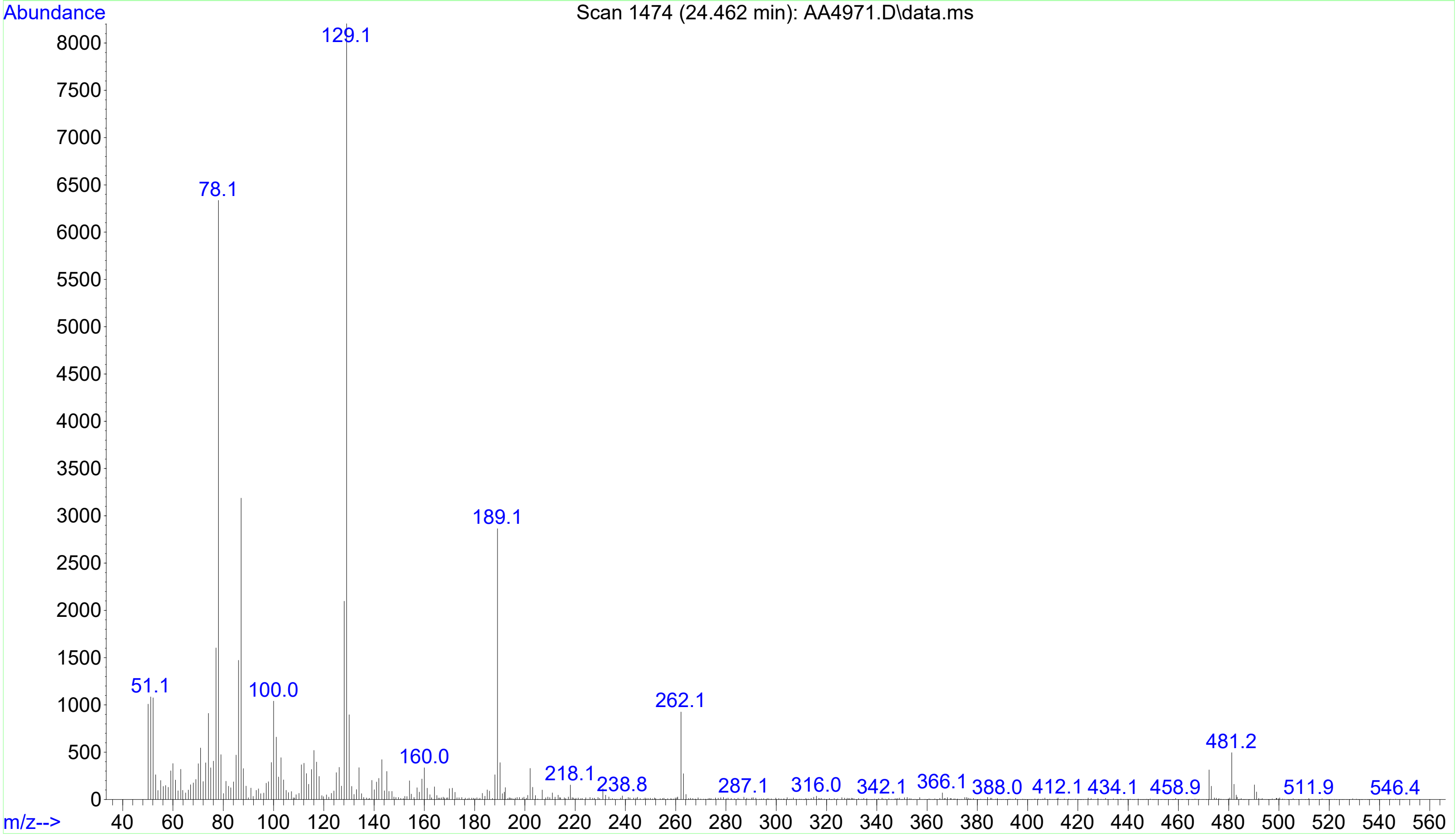

```
File        :E:\AA4971.D
Operator    : Artur
Acquired    : 17 Apr 2021   15:03      using AcqMethod SERVLABPMAA10TO1.M
Instrument  :     GC-MS AA
Sample Name: E. bicyclis Laminarin
Misc Info   :
Vial Number: 45
```

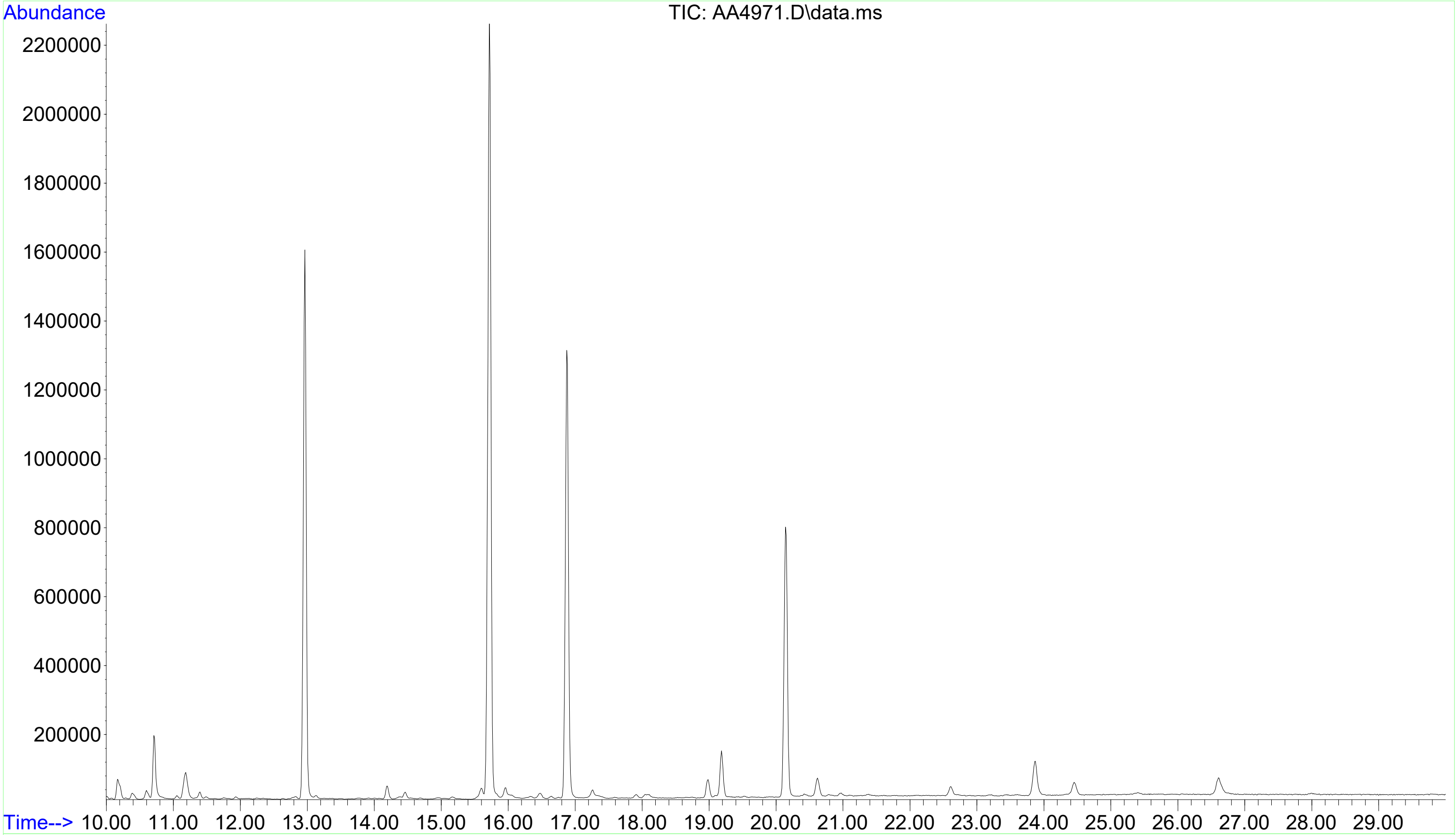

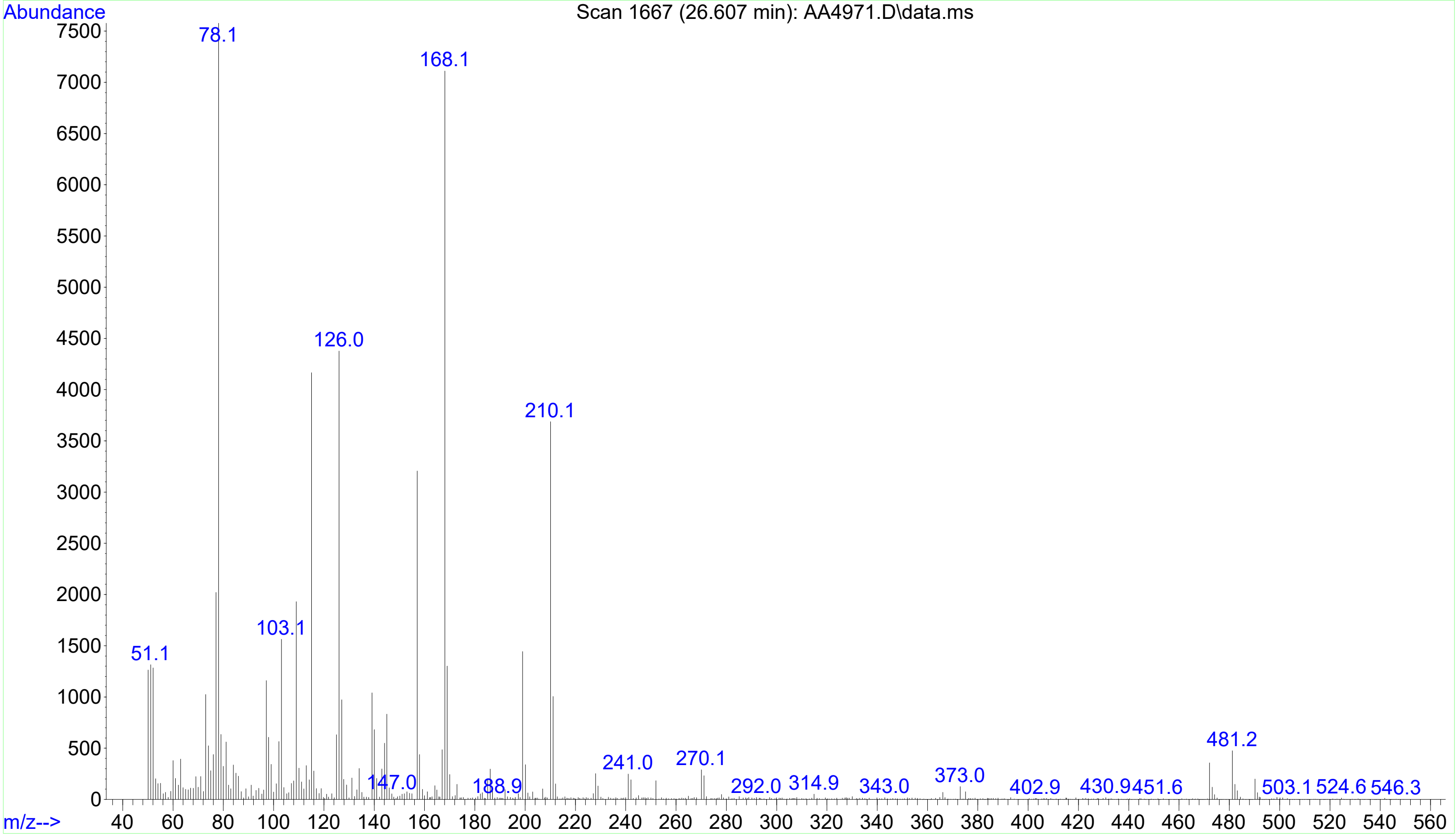

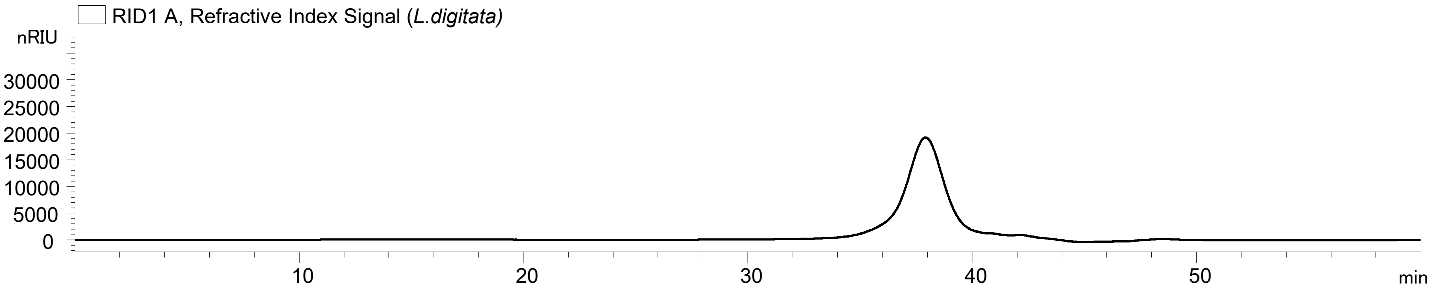
RID1 A, Refractive Index Signal (*L.digitata)*
nRIU
30000
25000
20000
15000
10000
5000
0
10
20
30
40
50
min

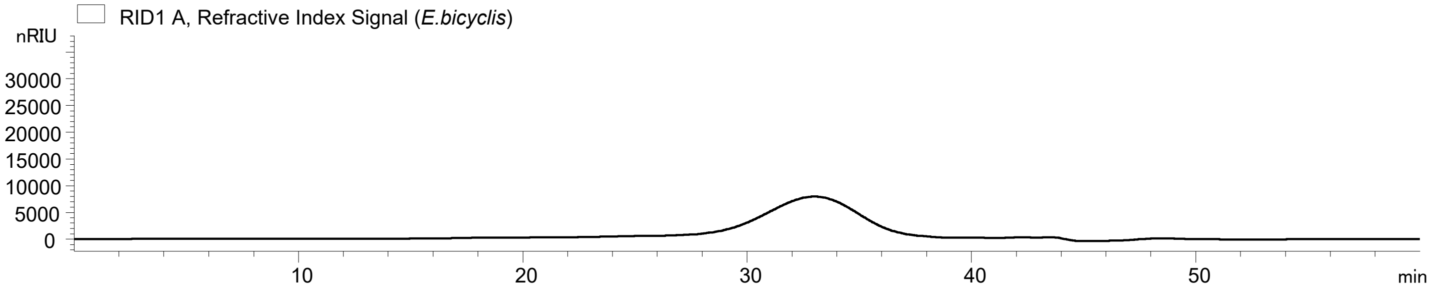
RID1 A, Refractive Index Signal (*E.bicyclis*)
nRIU
30000
25000
20000
15000
10000
5000
0
10
20
30
40
50
min
