# Supplementary material for: A glycan receptor kinase facilitates intracellular accommodation of arbuscular mycorrhiza and symbiotic rhizobia in the legume Lotus japonicus
Source: PLoS Biol. 2023 May 18;21(5):e3002127. doi: 10.1371/journal.pbio.3002127 (PMC10231839; doi:10.1371/journal.pbio.3002127)
Supplement: S5 Information — (DOCX) [file pbio.3002127.s021.docx]

**Suppl. Information.** NMR spectroscopy

*E.bicyclis* laminarin (11.4 mg) and *L. digitata* laminarin (11.3 mg) were dissolved in 90% DMSO (d6-99.96%, Sigma-Aldrich) and 10% D_2_O (d-99.9%, Sigma-Aldrich) and heated at 70°C for 5–10 min before being transferred to a 5-mm NMR tube (LabScape Stream, Bruker BioSpin AG, Switzerland). No visible aggregates were observed in the samples.
All homo- and heteronuclear experiments for *E. bicyclis* laminarin were recorded at 50°C on a Bruker 800 MHz Avance III HD equipped with a 5-mm cryogenic CP-TCI-z-gradient probe at the NV-NMR-Centre/NNP (Norwegian NMR Platform) at NTNU Norwegian University of Science and Technology, Trondheim, Norway. The following experiments were collected: 1D proton (zg30) acquired with a fid size of 65535, spectral width of 10 ppm, 16 scans and d1 = 1 s, ^1^H-^13^C HSQC (heteronuclear single quantum coherence) spectrum with multiplicity editing (hsqcedetgpsisp2.3) acquired with a fid size of 2048/512 (F2/F1), spectral width of 10/120 ppm (F2/F1), 16 scans and d1= 1.5 s, ^1^H-^13^C H2BC (heteronuclear two bond correlation) spectrum (h2bcetgpl3pr) acquired with a fid size of 2048/1024 (F2/F1), spectral width of 10/120 ppm (F2/F1), 32 scans and d1 = 1.5 s, ^1^H-^13^C HMBC (heteronuclear multiple bond coherence) spectrum with suppression of one-bond correlations (hmbcetgpl3nd) acquired with a fid size 2048/512 (F2/F1), spectral width of 10/120 ppm (F2/F1), 16 scans and d1 = 1.5 s, phase sensitive ^1^H-^13^C HSQC-TOCSY (total coherence spectroscopy) spectrum with DIPSI-2 using PEP and adiabatic inversion and refocusing pulses (hsqcdietgpsisp.2) acquired with a fid size 2048/400 (F2/F1), spectral width of 10/120 ppm (F2/F1), 16 scans and d1 = 1.5 s, d9 = 70 ms (mixing time for ^1^H-^1^H mixing), ^1^H-^1^H-IP-COSY (in-phase correlation spectroscopy) spectrum (ipcosyesgp-tr) acquired with a fid size 4096/256 (F2/F1), spectral width of 12/12 ppm (F2/F1), 16 scans and d1 = 1.5 s.

*L. digitata* laminarin NMR spectra were recorded at 60°C on a Bruker 800 MHz Avance Neo equipped with a 5-mm CPTXO Cryoprobe C/N-H-D at the Copenhagen Center for Open NMR Spectroscopy, Copenhagen, Denmark. The following experiments were collected: 1D proton (zg30) acquired with a fid size of 96774, spectral width of 20 ppm, 32 scans and d1 = 1 s, ^1^H-^13^C HSQC (heteronuclear single quantum coherence) spectrum with multiplicity editing (hsqcedetgpsisp2.3) acquired with a fid size of 1024/512 (F2/F1), spectral width of 15/80 ppm (F2/F1), 8 scans and d1 = 1 s, ^1^H-^13^C H2BC (heteronuclear two bond correlation) spectrum (h2bcetgpl3pr) acquired with a fid size of 2048/1024 (F2/F1), spectral width of 10/80 ppm (F2/F1), 32 scans and d1 = 1.5 s, ^1^H-^13^C HMBC (heteronuclear multiple bond coherence) spectrum with (hmbcgplpndqf) acquired with a fid size 2048/512 (F2/F1), spectral width of 13/80 ppm (F2/F1), 16 scans and d1 = 1.5 s, phase sensitive ^1^H-^13^C HSQC-TOCSY (total coherence spectroscopy) spectrum with DIPSI-2 using PEP and adiabatic inversion and refocusing pulses (hsqcdietgpsisp.2) acquired with a fid size 1024/512 (F2/F1), spectral width of 15/80 ppm (F2/F1), 16 scans and d1 = 1.5 s, ^1^H-^1^H-TOCSY spectrum (dipsi2etgpsil9) acquired with fid size 2048/512 (F2/F1), spectral width of 14/14 ppm (F2/F1), 8 scans, d1 = 2 s and d9 = 80 ms (mixing time), 1D selective TOCSY spectrum (seldigpzs) with a fid size 15624, spectral width of 9.76 ppm, 256 scans, O1 4.067 ppm, d1 = 2 s and d9 = 140 ms (mixing time), 1D selective NOESY spectrum (selnogpzs.2) with a fid size 15624, spectral width of 9.76 ppm, 256 scans, O1 4.067 ppm, d1 = 2 s and d8 = 300 ms (NOE mixing time).

Residual DMSO signal was used for chemical shift reference (^1^H 2.50 ppm, ^13^C 39.5ppm). The spectra were recorded using TopSpin 3.5 pL7 software (Bruker BioSpin) and processed and analysed with Topspin 4.1.1 software (Bruker BioSpin). The β-glucans were assigned by starting at the anomeric signal and then following the proton-proton connectivity using ^1^H-^1^H-TOCSY, 1D selective TOCSY, ^1^H-^1^H-IP-COSY, ^1^H-^13^C H2BC, ^1^H-^13^C HSQC-TOCSY for individual sugar units. ^1^H-^13^C HSQC was used for assigning the carbon chemical shifts, and ^1^H-^13^C HMBC and 1D selective NOESY provided information about the glycosidic linkages between the individual sugar units.

Proton and carbon chemical shifts (δ^1^H/ δ^13^C) assignment of *E. bicyclis* laminarin β-glucan

|  | δ^1^H/ δ^13^C (ppm) | | | | | |
| --- | --- | --- | --- | --- | --- | --- |
| Linkage (from 1) | 1 | 2 | 3 | 4 | 5 | 6, 6’ |
| (1,6) | 4.20/ 102.80 | 3.08/ 73.54 | 3.18/ 75.87 | 3.07/ 69.93 | 3.11/ 76.53 | 3.44, 3.66/ 60.90 |
| (1,6) | 4.22/ 103.20 | 2.99/ 73.41 | 3.17/ 76.30 | 3.07/ 69.91 | 3.13/ 76. 42 | 3.44, 3.66/ 60.90 |
| (1,6) | 4.23/ 103.15 | 3.00/ 73.41 | 3.13/ 76.50 | 3.13/ 69.75 | 3.31/ 75.39 | 3.58. 3.97/ 68.38 |
| (1,6) | 4.34/ 102.40 | 3.21/ 72.44 | 3.45/ 86.60 | 3.22/ 68.30 | 3.27/ 75.93 | 3,45, 3.67/ 60.60 |
| (1,3) | 4.36/ 103.80 | 3.09/ 73.52 | 3.23/ 75.80 | 3.10/ 70.10 | 3.43/ 75.06 | 3.48, 4.08/ 68.64 |
| (1,3) | 4.38/ 103.70 | 3.10/ 73.51 | 3.22/ 75.84 | 3.10/ 70.04 | 3.41/ 75.10 | 3.54, 4.01/ 68.46 |
| (1,3) | 4.40/ 103.70 | 3.08/ 73.62 | 3.23/ 75.83 | 3.14/ 70.06 | 3.32/ 75.36 | 3.58, 3.97/ 68.38 |
| (1,3) | 4.48/ 103.10 | 3.28/ 72.70 | 3.47/ 86.41 | 3.22/ 68.28 | 3.18/ 75.93 | 3.45, 3.65/ 60.90 |
| (1,3) | 4.50/ 102.90 | 3.28/ 72.48 | 3.46/ 87.05 | 3.27/ 68.40 | 3.19/ 75.89 | 3.45, 3.65/ 60.90 |
| (1,3) | 4.52/ 102.80 | 3.29/ 72.70 | 3.47/ 85.50 | 3.25/ 68.34 | 3.23/ 75.90 | 3.46, 3.66/ 60.60 |

Proton and carbon chemical shifts (δ^1^H/ δ^13^C) assignment of *L. digitata* laminarin β-glucan

|  | δ^1^H/ δ^13^C (ppm) | | | | | |
| --- | --- | --- | --- | --- | --- | --- |
| Linkage (from 1) | 1 | 2 | 3 | 4 | 5 | 6, 6’ |
| (1,6) | 4.22/ 103.37 | 3.01/ 74.00 | 3.21/ 76.55 | 3.09/ 70.51 | 3.14/ 76.99 | 3.45, 3.69/ 61.43 |
| (1,3) | 4.39/ 104.10 | 3.09/ 74.19 | 3.22/ 76.58 | 3.09/ 70.52 | 3.21/ 77.21 | 3.45, 3.69/ 61.43 |
| (1,3) | 4.52/ 103.40 | 3.30/ 73.31 | 3.49/ 86.40 | 3.23/ 68.65 | 3.27/ 76.58 | 3.45, 3.69/ 61.43 |
| (1,3) | 4.53/ 103.40 | 3.33/ 73.06 | 3.52/ 86.10 | 3.30/ 69.10 | 3-50/ 75.35 | 3.54, 4.07/ 68.88 |
