# Supplementary material for: A glycan receptor kinase facilitates intracellular accommodation of arbuscular mycorrhiza and symbiotic rhizobia in the legume Lotus japonicus
Source: PLoS Biol. 2023 May 18;21(5):e3002127. doi: 10.1371/journal.pbio.3002127 (PMC10231839; doi:10.1371/journal.pbio.3002127)
Supplement: Attachment — Submitted filename: Rebuttal letter_13.02.23.pdf [file pbio.3002127.s023.pdf]

Reviewer #1: In the manuscript entitled "A Lotus japonicus glycan receptor kinase facilitates intracellular accommodation of arbuscular mycorrhiza and symbiotic rhizobia" Kelly et al. identified a novel LysM-RLK protein EPR3a from L. japonicus. They performed biochemical, molecular, and functional characterization of EPR3a in arbuscular and legume-rhizobia symbioses. In this manuscript, the authors investigated the binding of EPR3a to branched glycans and the perception of rhizobial exopolysaccharide, which is one of the determinants of the specificity of plant-microbe interaction. The authors used LORE1 mutant lines of EPR3a for functional characterization of this protein in arbuscular mycorrhiza association. Finally, the authors used kinase assays to validate in vitro kinase activity of EPR3 and EPR3a. In my opinion, this manuscript suffers from significant flaws.
Response: We thank the reviewer for constructive comments and suggestions

1- Many essential controls and critical experiments are missing. This story is highly biased towards symbiosis while it seems likely that EPR3 is a general fungal MAMP receptor:
Response: ROS induction is a hallmark of MAMP defense recognition and both *L. digitata* and *E. bicyclis* laminarin induces ROS production in wild-type *Lotus*. We did not observe any significant change in laminarin ROS elicitation of *epr* mutants compared to wild-type and have included this data in a new Suppl Fig 11. In addition we tested the *S. indica* β-1,3/β-1,6 decasaccharide, which did not induce any ROS production at all in wild-type or *epr3a-2/epr3-11* plants (Suppl Fig 11C) and is consistent with Chandrasekar et al 2022. We have broadened our discussion to clarify that EPR3a functions as glycan surveillance receptor including glycan signal molecules from pathogens and infer that the EPR3 receptors are not defense MAMP receptors. Supporting this notion changes in defense response gene expression between wild-type, *epr3*- and *epr3a*- mutants was not detected in the RNAseq data set now included, see Suppl Fig 13. All the evidence available on Epr3 and Epr3a suggest a function as surveillance receptors distinguishing compatible and incompatible microbes by a mechanism differing from the typical MAMP defense response.

1a* The authors performed binding assays between EPR3a ectodomain and glycans obtained from algae (E. bicyclis and L. digitata)... and a fungus irrelevant to the biology of L. japonicus. Since they try to make a case for the role of EPR3a protein in AM symbiosis, the authors should have used fungal extracts from AM fungi and possibly pathogenic fungi relevant to L. japonicus. Using biologically relevant fungal cell wall extracts, the authors would have addressed whether the EPR3a activity is conserved across fungi, involving symbiotic and pathogenic interactions and whether symbiotic or pathogenic interaction dominates in virtue of EPR3a activity.
Response: AM fungi are obligate biotrophs and as appreciated by reviewer 2, purification of cell wall extracts has not been achieved by anybody and an approach to avoid substantial contamination from plant cell wall material is not available or easily conceived. We have now included results showing presence of β-1,6 glucan in AM extracellular matrix using the fluorescent labelled β-glucan-binding *Si*FGB1 lectin specific for β-1,6 glucan, see Fig 4. β-1,3 glucan has previously been shown to be present in the AM cell wall (Balestrini et al 1994). Together, this supports the relevance of binding assays performed using β-1,3/β-1,6 laminarins and the *S. indica* β-1,3/β-1,6 decasaccharide. We also want to emphasize that β-1,3/β-1,6 decasaccharide was isolated from EPS of both endophytic fungi *Serendipita indica* and pathogenic *Bipolaris sorokiniana* as mentioned and referenced in the manuscript.

1b* The authors checked the nodule number and arbuscular formation, the mature stages of root nodule, and AM symbiosis. Since the protein acts downstream of Nod factor perception, they should have checked the change in expression of genes involved in the SYM pathway, such as NIN and nuclear calcium spiking. Do glycans activate calcium spiking in L. japonicus? Do glycans activate or repress defense signaling in L. japonicus?
Response: Se our response to reviewer 2 comment 4

1c* Since the binding seems conserved with algal glycans, it seems likely that glycans from pathogenic fungi will bind EPR3a too. The authors should not frame their story so narrowly around symbiosis and test phenotypes of L. japonicus epr3a mutants with pathogenic fungi too.
Response: See response to comment 1 and notice that we have highlighted that the β-1,3/β-1,6 decasaccharide has been isolated from *S. indica* and the root pathogen *Bipolaris sorokiniana* (see Chandrasekar et al 2022)

1d* Since the authors used LORE1 mutants in this study which possess several insertions in the genome, it is not sure if the phenotype obtained is due to non-functional EPR3 and EPR3a only. It is necessary to perform some complementation assays to determine that the observed phenotypes are due to mutations in EPR3a or EPRw, not mutations in other genes.
Response: The LORE1 insertion sites in the EPR3 and EPR3a mutants were extracted from the LORE1 insertion sequence database and apart from the epr3a insertions no insertions are shared between the two epr3a alleles (Mun et al Sci. Rep. 6:39447 and Malolepszy et al Plant J 88, 306-317). We have now added this data in Suppl Table 1

1e* The authors performed an RT-qPCR in the root, nodules, and arbuscular mycorrhizal symbiosis. However, they have not shown any data on the expression of EPR3a in other plant tissues or response to fungal pathogens. While missing the expression analysis in other tissues, the authors neglected its role in different plant tissues or biotic interactions.
Response: We have added EPR3a expression data for other plant tissue, see Suppl Fig 4A and added a reference to the Lotus expression Atlas.

2- Inconsistencies between the data presented in figures and text.
2a* Line 102: "In this study, we report on the identification and characterization of a novel glycan receptor kinase in Lotus that we have designated EPR3a." The authors identified a novel receptor kinase; however, Line 125 says, "A wider search in the genome databases shows that this class of LysM-RLKs is widely conserved in the plant kingdom (22), with either EPR3a or EPR3 or both present in genomes of most plants forming endosymbiosis with mycorrhiza and/or rhizobia (26)." The authors have not presented any data to show the conservation of EPR3a in other plant genomes, and the same is not present in the references cited.
Response: We have now included structural analysis to support this point, see Suppl Fig 3.

2b* In the glycans binding assays in Fig. 4, the authors have not used the EPR3 binding ligand. EPR3 binding ligand would have ensured that the EPR3 protein used in the assay was functionally active. Additionally, there are two bands in the EPR3 lanes; can the authors give a plausible reason behind the two bands in the case of EPR3.
Response: EPR3 ectodomain preparations were tested using MST showing expected binding to *M. loti* EPS (Suppl Fig 10) and confirming functionality of protein preparations also used in gel affinity electrophoresis. Gel affinity assays using *M. loti* EPS is not feasible due to the electrostatic charge of extracted EPS, which destroys the acrylamide gel matrix during electrophoresis. We have not been able to conceive a way to optimize this without heavily chemically altering the EPS.
The two bands in the EPR3 lane of affinity gels are due to heterogeneous N-glycosylations. Two bands can also be seen for CERK1 and glycosylation is generally observed for all the ectodomains purified from insect cells (see also SDS-PAGE analysis in Suppl Fig 6).

2c* In Supplementary Fig. 6, the authors showed that EPR3 could bind with a low affinity to E. bicyclis glycan; however, the gel in Fig. 4 does not correlate with this statement.

Response: The affinity of EPR3 is not strong enough to affect the mobility and show a difference in affinity PAGE. There are differences between different assays, we have therefore where possible used more than one binding assay to validate results.

2d* EPR3a binds with a stronger affinity to S. indica decasaccharide (Kd:~51.2 µM) compared to E. bicyclis glycan (Kd:~247.7 µM), which again indicates that more assays are required to determine the binding affinity of EPR3a with pathogenic or symbiotic signals.
Response: As suggested by reviewer 2 we have included additional binding assays with well-defined linear glucans to support the higher affinity for 1-6 branched glucans like the decasaccharide and bacterial EPS. See additional data in the updated Fig 5 and Suppl Fig 7.

2e* As per Fig. 5, EPR3a shows higher binding affinity with R. leguminosarum EPS (Kd: ~12.5 µM) compared to M. loti EPS (Kd: ~44.4 µM). This result is contradictory as M. loti is a host for L. japonicus.
Response: The EPR3 and EPR3a are glycan surveillance receptors differentiating between compatible and incompatible glycans in a second step after Nod factor perception. *R. leguminosarum* and *S. meliloti* can infect and nodulate *Lotus japonicus* if equipped with the correct primary Nod factor signal molecules secreted (Pacios-Bras et al 2000 Mol Plant Microbe Interact 13: 475–479, Radutoiu et al 2007 EMBO J 26, 3923-3935) and thus EPS from *R. leguminosarum* appears to be compatible**.** See also Kawaharada et al 2015, 523, 308**.**

3- Some statements are inaccurate:
3a* Line 135: "In contrast, Epr3 expression did not differ between mock and AM treatments." however, the expression of EPR3 differed between mock and AM treatments at 2 DPI.
Response: We have rephrased this statement to take the 2DPI into account.

3b* In Line 171 authors state, "Arbuscule development in epr3a mutants appears indistinguishable from arbuscule development in wild-type plants, suggesting a role for EPR3a in fungal entry into cortical cells rather than in arbuscule accommodation." however, in line 182 authors make a statement that, "Localisation of Epr3a expression in arbusculated cells and the reduced frequency of arbuscule development in epr3a-1 and epr3a-2 mutants." These two statements are contradictory; maybe the authors wanted to make a statement about arbuscular structure.
Response: The reviewer is correct. We have replaced "development" in line 171 with "structure" to remove this contradiction.

Reviewer #2: The article by Kelly et al., describes the identification and characterization of a glycan receptor kinase, EPR3a, closely related to the exopolysaccharide (EPS) receptor EPR3 previously described. EPR3a seems to bind β-1,3 glucans with different β-1,6 branching patterns, which have been described to be characteristic of surface-exposed fungal glucans. EPR3a plays a function in the colonization of Arbuscular Mycorrhiza (AM), since epr3a mutants are defective in different steps of the colonization process. Since cell walls/surface of AM has been suggested to contain β-1,3/β-1,6 glucan, the authors conclude that EPR3a is involved in the perception of this type of glucans from AM. However, purification of these β-1,3/β-1,6 glucans from AM cell walls has not been either performed previously nor in the current article. The demonstration of β-1,3/β-1,6 glucans binding by the extracellular domains (EC), with LysM structures, of EPR2a and EPR has been demonstrated using two approaches: i) affinity gel electrophoresis assays, that is not a very accurate approach, and ii) MST, that is a more precise technology, though clearly has some limitations in EC-glucan binding assays due to precipitation of glucans at high concentration. Based on MST data, the authors conclude that EPR3a binds different types of β-1,3/β-1,6 glucans, but also rhizobial exopolysaccharide (EPS) that is mainly enriched in β-1,6 glucans. Notably, the affinities of EPR3a for

the complex mixtures of glucans tested are comparable to those previously described for EPS-EC-EPR3. Though the article lacks the characterization of the specific β-1,3/β-1,6 glucan structure/s recognized by EPR3a and EPR3, the authors show that their ECs bind β-GD, a well-defined β-1,3/β-1,6 glucan present in different fungi. Based on these findings they propose that the activity of EPR3-type receptors in plants is to monitor glycans produced by microbes during their colonization and to regulate
intracellular accommodation of microbes and the adaptative response of AM. The results of the article suggest contrasting expression patterns and the authors suggest divergent ligand affinities of EPR3a and EPR3 that explain their distinct functions in AM colonization and rhizobial infection in Lotus japonicus, though these differences in specificity for some ligands tested are quite low and accordingly some conclusion should be weakened. The article contains very interesting new data, and clearly contributes to increase the number of plants RLKs that have been shown to bind glycans, expanding our knowledge in this field of RLK-ligan (glycan) recognition.
Response: We thank the reviewer for constructive comments and suggestions and for sharing insight into the challenges for extracting relevant glucans and performing binding assays.

Major Points:
1. Purified glucans from AM fungal cell walls are not available, therefore, the binding capacity of EPR3a ectodomains was tested in native affinity gel electrophoresis and MST using β-glucans that might or not be present in AM cell walls. The laminarins used in the experiments, that are β-1,3 glucans with β-1,6 branched found in fungal cell wall, are quite complexes in their composition. Though the results of MST seem to be clear, the fact that these very complex mixtures of glucans were used for the binding assays do not contribute to clarify the minimal structure that is perceived by these ECs. Though the article shows that β-GD, a well-defined β-1,3/β-1,6 glucan is bound by both EC receptors, it is not clear if this minimal structure is present in AM cell walls. It would be very interesting to test the binding of short structures (Degree of Polymerizayion of 6 to 12) of pure linear β-1,3 and linear β-1,6 glucan in MST assays or ITC . Some of these pure oligosaccharides are commercially available. These analyses will clarify if β-1,6 branching is really required or not for ECs binding of β-glucans. It is hard to understand at the structural level that ECs of EPR3a and EPR could bind laminarin and EPS.
Response: We have followed the advice from reviewer 2 and performed MST binding studies with well-defined short linear β-1,3 glucan fragments to assess the contribution of β-1,6 branches on a β-1,3 core. Both EPR3a and EPR3 ectodomains show low affinity for linear β-1,3 oligosaccharides, laminaripentaose and laminarihexaose (600-800 µM range). This corresponds to at least a 10-fold lower affinity compared to the β-1,3/β-1,6 decasaccharide isolated from *S. indica* and *M. loti* EPS. Taken together this further indicates high affinity for β-glucans is gained through β-1,6 branches of a β-1,3 core saccharide. These results are shown in Fig 5, Suppl. Fig 7 and Suppl. Fig 10. None of the commercial suppliers were able to provide β-1,6 oligosaccharides and very low mM affinity was observed for the linear β-1,6 disaccharide, Gentiobiose that they could provide. This result is not included in the revised manuscript as LysM domains opposed to other lectins require at least tetrasaccharides for binding.

2. The use of glucan preparations (e.g. scleroglucan, pustulan) enriched in some β-glucans as negative controls might not be appropriate since, as the authors indicate, to work with these polymeric preparations is kind of risky because they contained a diversity of unknown structures and some binding/not binding results could be due to changes in solution viscosity (gelation) and precipitation, which is a well-known property of β-glucans at high concentrations. This is clearly a limitation of working with glucans, but some additional experiments (see 1) to further support authors conclusion could be performed.

Response: We appreciate the reviewers understanding of the challenges working with glucans and have followed the advice to perform binding assays with the currently available well-defined β-1,3 oligosaccharides.

3. Interestingly, EPR3a ectodomains can also bind rhizobial EPS from M. loti, S. meliloti, and R. leguminosarum with μM affinity, and the affinity for M. loti EPS is comparable to that of S. indica decasaccharide β-glucan. As indicated in 1, this is quite surprising and interesting, but also indicates that the binding specificity for some glucans is low or the glucan structures present in EPS and laminarin which are bound by ECs are more similar than anticipated.
Response: See our response to reviewer 1 comment 2e.

4. Surprisingly, epr3a epr3 double mutants were less severely impaired than epr3a single mutants, forming IT numbers comparable to epr3-11, This result suggests direct interaction between the EPR3a and EPR3 receptors or convergence of downstream signal transduction pathway. Based on these results, the authors suggests that EPR3 in the absence of EPR3a is a negative regulator and EPR3a normally acts to counter this negative regulation. However, this hypothesis has not been validated testing the regulation by EPR3a and EPR3 of downstream components of AM and rhizobia colonization pathways. The characterization of downstream events is in general poor in this article.
Response: We have now included our RNAseq expression analysis of downstream symbiosis and defense genes, see Suppl Fig 13. This data set was not included in the original submission since no change was detected. At first glance this is surprising. However, our phenotypic characterization presented here and in Kawaharada et al 2015 clearly show that the most drastic phenotypic effect is on root hair infection thread formation in *epr3*, *epr3a* and *epr3/epr3a* double mutants. It is well known that only very few root hair cells are responsive/infected and since these few responsive/infected cells can only be isolated together with an excessive amount of non-responsive cells, any transcriptional change would be diluted. We are therefore now moving towards a single cell analysis approach that we expect will reveal the downstream responses although we can´t exclude that a phosphorylation cascade acting on protein-level alone mediates the downstream response. Single cell sequencing and detailed phosphoproteomics are studies in themselves and outside the scope of this manuscript.

Minor Points
1. Legends of Figures with MST data are incomplete. Some concepts like n, different time points, etc. are not well explained in the legend to figures. The values in the Y axe is not explained either.
Response: Additional information now included

2. The authors used to ecotypes of Lotus japonica Gifu and Lotus Base (mutants background). Are those genotypes the same? This must be clarified.
Response: Indeed, we only work with the Gifu ecotype. We have clarified in text and Materials and Methods where appropriate.

3. Reference 27 is incomplete
Response: Corrected

4. Perception of β-GD, a well-defined β-1,3/β-1,6 glucan present in different fungi was found to be perceived by EPR3 and EPR3a receptors, but it has been described to be released by fungi for ROS scavenging beta-glucan to subvert immune responses. How this function of β-GD match with the binding data and functions of EPR3a and EPR3? Is Ros production altered in EPR3a and EPR3 mutants treated with β-GD?
Response: See our response to reviewer 1 comment 1

5. The Figure of the expression of EC proteins is not clear explained in Legends to Figures.
Response: We have improved explanations in the legends

COMMENTS FROM THE ACADEMIC EDITOR:

In particular, I would like to see the following major points addressed:
1) the limitations of using of crude preps from algae rather than relevant fungi or purified glucans was brought up by both reviewers and calls into question the specificity of the receptor. This should be addressed with either commercially available purified molecules or crude preps from AM fungi with relevant controls.
Response: Done. See our response to reviewer 1 comment 1a, our response to reviewer 2 comment 1 and Fig 4, Fig 5 and Suppl Fig 7.

2) I also agree that testing how EPR3a and EPR3 affect downstream regulation of symbiosis signaling is important to put these receptors into the context of the extensive literature.
Response: Done. See our response to reviewer 2 comment 4 and Suppl Fig 13

3) I do agree that the findings are biased towards AM fungi/symbiosis and it is entirely possible that the proteins also play a role in general immunity against fungi. I think addressing this point from reviewer 1 would strengthen the paper. However, I don't think their fundings or conclusions preclude this idea and so I think this could also be addressed by modifying the conclusions.
Response: We have included results showing ROS induction independent of EPR3 and EPR3a, see response to reviewer 1 point 1. All the evidence available on Epr3 and Epr3a suggest a function as surveillance receptors distinguishing compatible and incompatible microbes by a mechanism differing from the typical MAMP defense response.

4) a point-by-point response to all other concerns brought up by reviewers.
Response: Done
