# Supplementary material for: A glycan receptor kinase facilitates intracellular accommodation of arbuscular mycorrhiza and symbiotic rhizobia in the legume Lotus japonicus
Source: PLoS Biol. 2023 May 18;21(5):e3002127. doi: 10.1371/journal.pbio.3002127 (PMC10231839; doi:10.1371/journal.pbio.3002127)
Supplement: Attachment — Submitted filename: Rebuttal letter_07.04.23.pdf [file pbio.3002127.s024.pdf]

Editor: Based on the reviews and our Academic Editor's assessment of your revision, we are likely to accept this manuscript for publication, provided you satisfactorily address the remaining points raised by the reviewers. You should respond to the reviewer's concerns and we consider that the issues can be addressed by text modifications.

Response: We are pleased about the positive evaluation by editors and reviewers and have addressed the remaining points of concern below.

Regarding the issue about "pure structures should be tested", we think you should clarify earlier in the manuscript that "laminarin, which has the beta-1,6 branched beta-1,3 glucans also found in fungal cell walls" is a mix of different beta-1,6 branched beta 1,3 glucans as you resolve later by NMR.

Response: We have now rephrased this sentence on page 8, line 193-194, with the introduction of laminarins to clarify this point. "… using laminarin, which contains a mix of β-1,6 branched β-1,3 glucans also found in fungal cell walls (see NMR characterization below)."

Regarding EPR3 having similar binding affinity as EPR3a (page 27, line 238-240), you show that EPR3a uniquely binds to beta-1,6 branched beta-1,3 glucans while both bind to beta-1,3 glucans with similar affinity, and we think that this does not undermine the central conclusions of the manuscript.

Response: We agree and refer to our response to reviewer 2 on ligand binding below.

Regarding the ROS data, we consider that pathogen assays are beyond the scope of the manuscript.

Response: We agree and refer to our observations suggesting EPR3a and EPR3 do not function in typical defence responses elicited by β-glucans.

Please also make sure to address the following data and other policy-related requests.

Response: We have provided the additional data files.

Reviewer #2: This is a clear improved version of the previous submitted article. Authors have clarified the majority of questions addressed, and have completed the majority of the information that was lacking in the previous article.

Response: We thank the reviewer for suggestions and comments on improving our manuscript.

The articles now shows that EPR3a binds β-1,3 glucans (Lam5 and Lam6) though with lower affinity than β-glucans with β-1,6-branches. However, this analysis is incomplete (additional pure structures should be tested) but based on the β-glucans available for comparison we might accept the experiments

performed as those that were feasible. Surprisingly, the difference in specificity of EPR and EPR3a for the decasaccharide β-glucan is very low (it is almost identical).

Response: We have not been able to obtain additional pure oligosaccharides from commercial suppliers or experts in the field, other than the linear β-1,3 oligosaccharides tested (LAM5 and LAM6) and the β-1,6 disaccharide gentiobiose, as explained in the previous rebuttal letter. The laminarins represent the only available β-1,6-branched β-1,3 carbohydrates and our binding analysis of the pure *S. indica* β-1,6/β-1,3 decasaccharide complements the experimental pitfalls of working only with laminarins. However, we believe that our comparative binding assays sufficiently address and show how β-1,6-branched β-1,3 carbohydrates are specifically recognized by EPR-type receptors. We indeed show EPR3 and EPR3a bind the *S. indica* decasaccharide equally well, although we show a divergence in ligand specificity between EPR3 and EPR3a for the *E. bicyclis* laminarin. Together with our MS and NMR analysis of the laminarins, this implies EPR3a is specific towards one or more β-1,6/β-1,3 patterns found in the *E. bicyclis* laminarin. Elucidation of this specificity is not trivial and would require complex chemical synthesis of various β-1,6/β-1,3 glucans and validation that microorganisms produce these structures, which is beyond the scope of this study. Together with the clearly distinct expression profiles, we believe this does not undermine our conclusions on the distinct roles for EPR3a and EPR3 in rhizobial and arbuscular mycorrhizal symbioses.

The RNaseq data clarify some of the questions regarding the transcriptional responses activated. It is a shame that the authors did not include in the RNaseq analyses the beta-glucan that they used for binding experiments for comparison since this had been provided cleaner information about the responses triggered by the ligand and not just by the whole microorganism.

Response: Our data shows the expression of both *Epr3a* and *Epr3* is very low in uninoculated plants. In both cases, expression is only induced downstream of the initial perception of the symbionts and only in the very few cells that are infected or targeted for infection. In light of the included RNA-seq experiment and ROS assay addressing the immunity aspects, it is therefore very likely that any differential responses related to EPR functions when challenging mutant plants purely with β-glucans would be undetectable. Our data using whole organisms clearly show that single cell analysis, outside the scope of this manuscript, would be the realistic approach.

Last, the ROS response observed upon treatment with the β-glucan provides important information. However, since the decasaccharide is not triggering ROS, as described previously, it seems that ROS production observed upon L. digitata laminarin treatment, that is not reduced in EPR and EPR3a mutants, should be triggered by other β-glucan structures distinct from the decasaccharide. The interpretation of the authors might not be correct. I agree with reviewer 2 that the infection of mutants with a Lotus pathogen will contribute to clarify some key aspects of the function of the EPR3a receptor, but these experiments could be the matter of a different history.

Response: Our data shows laminarin induced ROS production is independent of EPR3 and EPR3a and we therefore conclude that EPR3 and EPR3a do not function in triggering typical MAMP immunity responses. Our RNA-seq data further supports this notion. We agree that it is likely other β-glucan structures in laminarin distinct from the *S. indica* decasaccharide which triggers ROS production. We

refer to references 35-37 that implicate linear β-1,3 glucans, which EPR3a and EPR3 have low affinity toward, induce immunity in plants.
